# High-quality genome assembly enables prediction of allele-specific gene expression in hybrid poplar

**DOI:** 10.1093/plphys/kiae078

**Published:** 2024-02-27

**Authors:** Tian-Le Shi, Kai-Hua Jia, Yu-Tao Bao, Shuai Nie, Xue-Chan Tian, Xue-Mei Yan, Zhao-Yang Chen, Zhi-Chao Li, Shi-Wei Zhao, Hai-Yao Ma, Ye Zhao, Xiang Li, Ren-Gang Zhang, Jing Guo, Wei Zhao, Yousry Aly El-Kassaby, Niels Müller, Yves Van de Peer, Xiao-Ru Wang, Nathaniel Robert Street, Ilga Porth, Xinmin An, Jian-Feng Mao

**Affiliations:** State Key Laboratory of Tree Genetics and Breeding, National Engineering Research Center of Tree Breeding and Ecological Restoration, Beijing Advanced Innovation Center for Tree Breeding by Molecular Design, National Engineering Laboratory for Tree Breeding, Key Laboratory of Genetics and Breeding in Forest Trees and Ornamental Plants, Ministry of Education, College of Biological Sciences and Technology, Beijing Forestry University, Beijing 100083, China; State Key Laboratory of Tree Genetics and Breeding, National Engineering Research Center of Tree Breeding and Ecological Restoration, Beijing Advanced Innovation Center for Tree Breeding by Molecular Design, National Engineering Laboratory for Tree Breeding, Key Laboratory of Genetics and Breeding in Forest Trees and Ornamental Plants, Ministry of Education, College of Biological Sciences and Technology, Beijing Forestry University, Beijing 100083, China; Key Laboratory of Crop Genetic Improvement & Ecology and Physiology, Institute of Crop Germplasm Resources, Shandong Academy of Agricultural Sciences, Ji’nan 250100, China; State Key Laboratory of Tree Genetics and Breeding, National Engineering Research Center of Tree Breeding and Ecological Restoration, Beijing Advanced Innovation Center for Tree Breeding by Molecular Design, National Engineering Laboratory for Tree Breeding, Key Laboratory of Genetics and Breeding in Forest Trees and Ornamental Plants, Ministry of Education, College of Biological Sciences and Technology, Beijing Forestry University, Beijing 100083, China; Rice Research Institute, Guangdong Academy of Agricultural Sciences & Key Laboratory of Genetics and Breeding of High Quality Rice in Southern China (Co-construction by Ministry and Province), Ministry of Agriculture and Rural Affairs & Guangdong Key Laboratory of New Technology in Rice Breeding, Guangzhou 510640, China; State Key Laboratory of Tree Genetics and Breeding, National Engineering Research Center of Tree Breeding and Ecological Restoration, Beijing Advanced Innovation Center for Tree Breeding by Molecular Design, National Engineering Laboratory for Tree Breeding, Key Laboratory of Genetics and Breeding in Forest Trees and Ornamental Plants, Ministry of Education, College of Biological Sciences and Technology, Beijing Forestry University, Beijing 100083, China; State Key Laboratory of Tree Genetics and Breeding, National Engineering Research Center of Tree Breeding and Ecological Restoration, Beijing Advanced Innovation Center for Tree Breeding by Molecular Design, National Engineering Laboratory for Tree Breeding, Key Laboratory of Genetics and Breeding in Forest Trees and Ornamental Plants, Ministry of Education, College of Biological Sciences and Technology, Beijing Forestry University, Beijing 100083, China; State Key Laboratory of Tree Genetics and Breeding, National Engineering Research Center of Tree Breeding and Ecological Restoration, Beijing Advanced Innovation Center for Tree Breeding by Molecular Design, National Engineering Laboratory for Tree Breeding, Key Laboratory of Genetics and Breeding in Forest Trees and Ornamental Plants, Ministry of Education, College of Biological Sciences and Technology, Beijing Forestry University, Beijing 100083, China; State Key Laboratory of Tree Genetics and Breeding, National Engineering Research Center of Tree Breeding and Ecological Restoration, Beijing Advanced Innovation Center for Tree Breeding by Molecular Design, National Engineering Laboratory for Tree Breeding, Key Laboratory of Genetics and Breeding in Forest Trees and Ornamental Plants, Ministry of Education, College of Biological Sciences and Technology, Beijing Forestry University, Beijing 100083, China; State Key Laboratory of Tree Genetics and Breeding, National Engineering Research Center of Tree Breeding and Ecological Restoration, Beijing Advanced Innovation Center for Tree Breeding by Molecular Design, National Engineering Laboratory for Tree Breeding, Key Laboratory of Genetics and Breeding in Forest Trees and Ornamental Plants, Ministry of Education, College of Biological Sciences and Technology, Beijing Forestry University, Beijing 100083, China; State Key Laboratory of Tree Genetics and Breeding, National Engineering Research Center of Tree Breeding and Ecological Restoration, Beijing Advanced Innovation Center for Tree Breeding by Molecular Design, National Engineering Laboratory for Tree Breeding, Key Laboratory of Genetics and Breeding in Forest Trees and Ornamental Plants, Ministry of Education, College of Biological Sciences and Technology, Beijing Forestry University, Beijing 100083, China; State Key Laboratory of Tree Genetics and Breeding, National Engineering Research Center of Tree Breeding and Ecological Restoration, Beijing Advanced Innovation Center for Tree Breeding by Molecular Design, National Engineering Laboratory for Tree Breeding, Key Laboratory of Genetics and Breeding in Forest Trees and Ornamental Plants, Ministry of Education, College of Biological Sciences and Technology, Beijing Forestry University, Beijing 100083, China; School of Agriculture, Ningxia University, Yinchuan 750021, China; Yunnan Key Laboratory for Integrative Conservation of Plant Species with Extremely Small Populations, Key Laboratory for Plant Diversity and Biogeography of East Asia, Kunming Institute of Botany, Chinese Academy of Sciences, Kunming 650201, Yunnan, China; College of Forestry, Shandong Agricultural University, Tai’an 271000, China; Umeå Plant Science Centre, Department of Ecology and Environmental Science, Umeå University, SE-901 87 Umeå, Sweden; Department of Forest and Conservation Sciences, Faculty of Forestry, University of British Columbia, Vancouver, Bc, V6T 1Z4, Canada; Thünen-Institute of Forest Genetics, 22927 Grosshansdorf, Germany; Department of Plant Biotechnology and Bioinformatics, Ghent University, 9052 Ghent, Belgium; VIB Center for Plant Systems Biology, 9052 Ghent, Belgium; Centre for Microbial Ecology and Genomics, Department of Biochemistry, Genetics and Microbiology, University of Pretoria, Pretoria 0028, South Africa; College of Horticulture, Academy for Advanced Interdisciplinary Studies, Nanjing Agricultural University, Nanjing 210095, China; Umeå Plant Science Centre, Department of Ecology and Environmental Science, Umeå University, SE-901 87 Umeå, Sweden; Umeå Plant Science Centre, Department of Plant Physiology, Umeå University, SE-901 87 Umeå, Sweden; Départment des Sciences du Bois et de la Forêt, Faculté de Foresterie, de Géographie et Géomatique, Université Laval, Québec, QC G1V 0A6, Canada; State Key Laboratory of Tree Genetics and Breeding, National Engineering Research Center of Tree Breeding and Ecological Restoration, Beijing Advanced Innovation Center for Tree Breeding by Molecular Design, National Engineering Laboratory for Tree Breeding, Key Laboratory of Genetics and Breeding in Forest Trees and Ornamental Plants, Ministry of Education, College of Biological Sciences and Technology, Beijing Forestry University, Beijing 100083, China; State Key Laboratory of Tree Genetics and Breeding, National Engineering Research Center of Tree Breeding and Ecological Restoration, Beijing Advanced Innovation Center for Tree Breeding by Molecular Design, National Engineering Laboratory for Tree Breeding, Key Laboratory of Genetics and Breeding in Forest Trees and Ornamental Plants, Ministry of Education, College of Biological Sciences and Technology, Beijing Forestry University, Beijing 100083, China; Umeå Plant Science Centre, Department of Plant Physiology, Umeå University, SE-901 87 Umeå, Sweden

## Abstract

Poplar (*Populus*) is a well-established model system for tree genomics and molecular breeding, and hybrid poplar is widely used in forest plantations. However, distinguishing its diploid homologous chromosomes is difficult, complicating advanced functional studies on specific alleles. In this study, we applied a trio-binning design and PacBio high-fidelity long-read sequencing to obtain haplotype-phased telomere-to-telomere genome assemblies for the 2 parents of the well-studied F_1_ hybrid “84K” (*Populus alba* × *Populus tremula* var. *glandulosa*). Almost all chromosomes, including the telomeres and centromeres, were completely assembled for each haplotype subgenome apart from 2 small gaps on one chromosome. By incorporating information from these haplotype assemblies and extensive RNA-seq data, we analyzed gene expression patterns between the 2 subgenomes and alleles. Transcription bias at the subgenome level was not uncovered, but extensive-expression differences were detected between alleles. We developed machine-learning (ML) models to predict allele-specific expression (ASE) with high accuracy and identified underlying genome features most highly influencing ASE. One of our models with 15 predictor variables achieved 77% accuracy on the training set and 74% accuracy on the testing set. ML models identified gene body CHG methylation, sequence divergence, and transposon occupancy both upstream and downstream of alleles as important factors for ASE. Our haplotype-phased genome assemblies and ML strategy highlight an avenue for functional studies in *Populus* and provide additional tools for studying ASE and heterosis in hybrids.

## Introduction

Hybridization can lead to heterosis or hybrid vigor in which hybrids exhibit superior performance compared to either parent (Sprague 1983; Stuber and Janick 1994; Liu et al. 2021b). Hybrid vigor has been widely explored in agriculture and plant breeding, for example, in rice (*Oryza sativa*) (Huang et al. 2016; Cui et al. 2020; Zhang et al. 2021a), maize (*Zea mays*) (Sprague 1983; Duvick 1999; Xiao et al. 2021), vegetables (Yu et al. 2021), and some perennials (Chen et al. 2020b). In forestry, poplars (*Populus* spp.) (Eckenwalder 1984a, 1984b, 1996; McKown and Guy 2018) and willows (*Salix* spp.) (Serapiglia et al. 2015; Carlson et al. 2017, 2022; Fabio et al. 2017; Carlson and Smart 2022) have long been recognized for producing transgressive phenotypes. For example, hybrid shrub willow bioenergy crops often display heterosis for yield (Carlson and Smart 2022). The interspecific hybrid “84K” (*Populus alba ×Populus tremula* var. *glandulosa*) that we used in the current study exhibits faster growth rates and better adaptability to different environments than either of the parent species (Qiu et al. 2019).

The genetics of heterosis is still not fully understood (Chen 2010; Blum 2013; Schnable and Springer 2013). One mechanism is the interactions among parental alleles in the hybrid genome that result in allele-specific expression (ASE), which refers to the preferential expression of a specific parental allele in hybrids (Gaur et al. 2013). ASE has been demonstrated in mammals (Knight 2004) and plants (e.g. rice (*O. sativa*) (Chodavarapu et al. 2012), barley (*Hordeum vulgare*) (von Korff et al. 2009), *Salvia splendens* (Jia et al. 2021), and apple (*Malus domestica*) (Tian et al. 2022)). Several studies suggest that ASE plays a key role in heterosis (Springer and Stupar 2007; Paschold et al. 2012; Goff and Zhang 2013; Shao et al. 2019). However, the specific mechanisms regulating ASE are not well-known (Paschold et al. 2014; Shao et al. 2019).

The differential expression of alleles is influenced by several factors and is currently under investigation. *Cis/trans*-regulation is an important form of pre-transcriptional regulation in which the *cis*-regulatory element and *trans*-acting factor interact to alter gene expression levels and influence trait regulation (Bao et al. 2019). In plants, ASE analyses have shown numerous *cis* regulatory divergences within species (Zhang and Borevitz 2009; Bell et al. 2013) and between species (Lemmon et al. 2014; Combes et al. 2015), and in response to abiotic stress (Lovell et al. 2016; Waters et al. 2017). Furthermore, genetic variation and epigenetic changes resulting from “genomic shock” in the hybrid also affect ASE (Bird et al. 2018; Vohra et al. 2020). The insertion of transposable elements (TEs) in and around genes can terminate or modulate gene expression by disrupting normal gene structure or regulatory motifs, thereby resulting in ASE. In apple (“Royal Gala”), TE insertion regulates ASE and is responsible for flower color variation (Tian et al. 2022) and fruit size increase (Yao et al. 2001). ASE in hybrid rice is associated with DNA methylation differences, and is negatively related to allele-specific methylation of CHG (Ma et al. 2021). These studies showed the factors that can alter allelic expression in hybrids under different conditions that can lead to heterosis (Shao et al. 2019), but accurate identification of specific alleles and structural variants (SV) as well as their associated impact on gene expression are only possible when high-quality genome/subgenome assemblies are available.

*P. alba* and *P. tremula* are sympatric and often form natural hybrids. *P. alba*, silver poplar, is widely distributed in Europe and Central Asia (Brundu et al. 2008) and is resistant to pests, fungi, and bacterial pathogens and can withstand various environmental stresses such as drought, wind, salt, and low temperatures (Brundu et al. 2008). In comparison, *P. tremula*, Eurasian aspen, is native to cool temperate regions of Europe and Asia. In 1984, the poplar clone “84K” (*P. alba* × *P. tremula* var. *glandulosa*) was first introduced to China from South Korea and became widely planted and used due to its fast growth rates and high wood quality (Wang et al. 2005). In addition, “84K” has become an important model system for *Populus* studies due to its ease of vegetative propagation and tissue culture, suitability for efficient genetic transformation, and wide adaptation to different environments (Zhang et al. 2011; Ke et al. 2017; Yoon et al. 2018). Understanding the genetic mechanisms behind the elite allelic combination in “84K” is valuable in studying heterosis and augmenting future breeding progress.

Here, we used Pacific bioscience high-fidelity (PacBio HiFi) long-read sequencing, ultra-long Oxford nanopore technology sequencing (ONT), and high-throughput chromosome conformation capture (Hi-C) to assemble the 2 haplotype genomes of the interspecific F_1_ hybrid of the poplar clone “84K” using a trio-binning design, and recovered 2 gap-free haplotype parental genomes (with only 2 small gaps in 1 chromosome). Local and structural variations between genomes were revealed by whole genome alignment. We used large-scale RNA-sequencing data from 156 biological samples of 9 tissues under various conditions for gene identification annotation, and to examine ASE. In addition, 46 genetic and epigenetic features were incorporated into machine-learning (ML) modeling of ASE to identify key factors potentially affecting ASE. We show that ASE can be predicted with high accuracy by ML models that incorporate DNA methylation, sequence divergence, and TE occupancy. This study (1) provides high-quality genomic data for poplar functional genomics, (2) demonstrates the value of ML modeling in complex biological data analysis, and (3) contributes to further functional genomics work on ASE and heterosis.

## Results

### High-quality haplotype-phased genome assemblies

The haploid genome size of the poplar clone “84K” is 427.2 Mb according to *k*-mer estimates (Qiu et al. 2019) and 474.43 Mb (Huang et al. 2021), and 470.155 ± 5.94 Mb according to flow cytometry estimates (Qiu et al. 2019) (Supplementary Fig. S1). High heterozygosity (∼2.1%) already suggested that differentiation exists between parental genomes (Qiu et al. 2019; Huang et al. 2021). We produced 70 Gbp (∼80× coverage, N50 10 kb) Pacific Bioscience HiFi sequences, 80 Gbp (∼90×, N50 28 kb) ONT sequences, and 100 Gbp Illumina Hi-C paired-end reads (Table 1 and Supplementary Table S1). It is worth noting that our ONT sequencing data contained 36 Gbp ultra-long reads (N50 40 kb, 627 kb of maximum length). We also collected short-reads of Illumina sequencing for both parents and their “84K” hybrid clone, including 31 Gbp (∼60×) of the female parent *P. alba* (Muller et al. 2020), 47 Gbp (∼90×) of the male parent *P. tremula* var. *glandulosa* (Huang et al. 2021), and 50 Gbp (∼50×) of “84K” (Qiu et al. 2019) (Supplementary Table S2).

**Table 1.** Comparison of the genome in this study to previously published assemblies of the poplar clone “84K”

Species	Subgenome A (*P. alba*)	Subgenome G *(P. glandulosa)*	84K	Qiu et al. (2019)	Huang et al. (2021)
Genome size (Mb)	400.19	416.76	816.95	753.82	781.36
Number of scaffolds	23	27	50	–	–
N50 of scaffolds (Mp)	21.76	23.41	22.36	–	–
Number of contigs	25	27	52	1,384	2,109
N50 of contigs (Mp)	21.76	23.41	22.36	2.24	3.66
Number of Gap	2	0	2	505	–
Complete BUSCOs (%)	96.67	96.67	96.50	96.30	97.00
GC content of the genome (%)	34.69	35.05	34.87	-	34.12
Number of predicted genes	36,746	37,853	74,599	85,755	-
Number of predicted protein-coding genes	33,170	33,166	66,336	72,574	77,150
Raw bases of WGS-HiFi Sequel (Gb)	–	–	70.66 (80×)	–	–
Raw bases of WGS-ONT Sequel (Gb)	–	–	80.82 (90×)	–	–
Raw bases of Hi-C (Gb)	–	–	101.97 (120×)	95.27 (202.7×)	–
Raw bases of WGS-Illumina HiSeq (Gb)	–	–	48.58 (50×)	–	30 (∼ 63.2×)
Raw bases of WGS-Illumina Novaseq (Gb)	–	–	–	56.3 (119.79×)	46.5
Raw bases of WGS-PacBio (Gb)	–	–	–	48.44 (103.07×)	59.72 (∼ 125.9×)

Subgenomes A and G represent the two haplotype genome assemblies. Subgenome A represents the assembly of one parent, i.e. *P. alba*, and subgenome G represents that of the other parent, i.e. *P.tremula* var. *glandulosa*. The “84K” genome represents the genome assembly of the heterozygous diploid. –: data not available.

**Figure 1.**
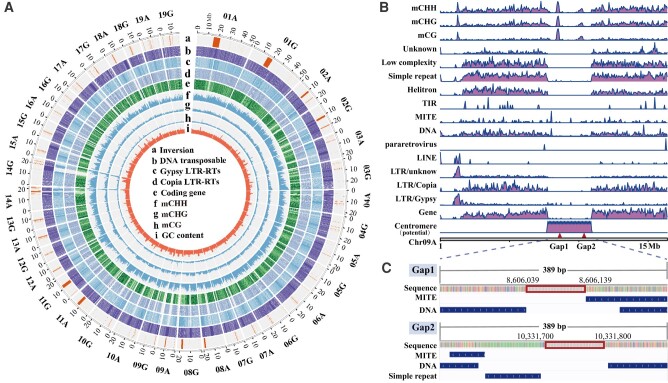
Genomic characterization. **A)** Synteny and distribution of genomic features of the poplar clone “84K”. (a) density of inversions (or inverted regions), (b) distribution of DNA-like transposons, (c) istribution of *Gypsy* LTR-RTs, (d) distribution of *Copia* LTR-RTs, (e) distribution of coding genes, (f)–(h) average methylation levels of CHH, CHG, and CG, respectively, and (i) histogram of GC content in 50 kb nonoverlap sliding windows. **B)** Distribution of DNA methylations, repeat elements around the 2 remaining gaps in the assembly. Two triangles indicate the location of the 2 gaps on chromosome 9A (chr09A). LINE, long interspersed repetitive element; LTR-RT, long terminal repeat retrotransposon; MITE, miniature inverted repeat transposable element; TIR, terminal inverted repeats. **C)** The detailed location of the gaps and the adjacent repeat elements. The rectangular boxes represent the gaps. The length of each gap was set to 100 bp arbitrarily.

Based on these data, we assembled the “84K” genome stepwise to finally obtain the haplotype-phased assembly (Fig. 1A and Supplementary Tables S3 and S4). First, the PacBio HiFi reads were directly assembled with hifiasm v0.13-r308 (Cheng et al. 2021), and the Illumina short-reads from both parents were added for trio-binning. Second, we mapped the Hi-C sequencing reads against the assembly to obtain a normalized contact matrix. The matrix was then used for scaffolding, followed by manual adjustment (e.g. adjusting boundaries, removing incorrect insertions, and realigning). Gaps were then filled using ONT ultra-long reads and the assembled ONT contigs. Finally, 2 rounds of polishing were performed using NextPolish v1.1.0 based on HiFi reads and Illumina short-reads (Supplementary Fig. S2). After a rigorous assembly process and extensive manual curation, we obtained a haplotype-phased genome assembly, with a gap-free haplotype assembly of *P. tremula* var. *glandulosa* (416.8 Mb, subgenome G) and a haplotype assembly of *P. alba* containing only 2 gaps (400.2 Mb, subgenome A) (Table 1 and Supplementary Table S4). Compared to the 2 previous versions of an “84K” genome assembly, the overall contiguity increased by 8.98-fold (Qiu et al. 2019) and by 5.11-fold (Huang et al. 2021), respectively (Table 1). In addition, we assembled the complete cyclic mitochondrial and chloroplast genomes with genome sizes of 839 and 157 kb, respectively.

### Centromere and telomere annotation

Since centromeric regions may have abundant tandem repeats, we first identified regions with a high occurrence of tandem repeats. Then putative centromeres were determined based on a tandem repeat with the highest abundance. Potential centromeric regions were found for 28 chromosomes (Supplementary Fig. S3). Furthermore, we identified all 38 telomeres through the mapping of telomere repeats. Notably, short telomere repeats were observed at both ends of chr02 and chr04 in the “G” haplotype genome. (Supplementary Fig. S4).

As mentioned earlier, the *P. alba* assembly (subgenome A) contained only 2 small gaps located within the potential centromeric region of chromosome 9 (chr09A) (Figs. 1, B and C and Supplementary Fig. S5). DNA transposons [miniature inverted repeat transposable element (MITE) and DNA subfamilies] and simple repeats were found to be enriched in this centromeric region (Fig. 1B), indicating that these assembly gaps are potentially associated with DNA transposons.

### Validation of the “84K” genome assembly

We performed a series of analyses to assess the quality of the genome assembly. First, we mapped the Hi-C reads to the assembled haplotype-phased genome and found that all chromosomes had correct arrangement and alignment (Supplementary Fig. S6). Interestingly, the 5′ end of chr11G of *P. tremula* var. *glandulosa* showed a clear chromatin contact signal in the Hi-C data, which was also observed in a previous “84K” assembly (Qiu et al. 2019) (Supplementary Fig. S6). In addition, we mapped the Illumina paired-end, HiFi, and ONT reads to our genome assembly, and found that 96.6%, 97.8%, and 99.6% of reads, respectively, were successfully mapped, with >98% of the assembly having >10-fold coverage (Supplementary Table S5 and Supplementary Fig. S7). In addition, benchmarking universal single-copy orthologs (BUSCO) (Simao et al. 2015) scores of both subgenomes A and G equaled 96.7% (Table 1 and Supplementary Table S6). Finally, we found that the 2 subgenomes showed strong collinearity with *Populus trichocarpa* (v4.1) (Tuskan et al. 2006) and the previously published “84K” genome (Qiu et al. 2019), with no major inter- and intra-chromosomal translocations detected (Supplementary Figs. S8 and S9). These results demonstrate the high completeness and accuracy of our genome assembly.

### Genome annotation

To generate a high-resolution gene annotation for the “84K” clone, we collected 156 RNA-seq samples from 9 different tissues (bark, leaf-derived callus, defoliated stems from apex to fourth internodes, internode, leaf, leafy stems from 3-week-old plants, root, stem, and xylem) under 8 different experimental conditions (Supplementary Data Sets 1 and 2). In addition, to avoid missing genes with transient expression or genes with extremely low expression that are difficult to detect with RNA-seq data, we combined evidence based on protein homology and ab initio prediction to predict protein-coding genes (PCGs) in the repeat-masked genomes. In total, the “84K” genome contained 66,336 (subgenome A: 33,166; subgenome G: 33,170) high-confidence PCGs (Supplementary Table S7), covering 1,409 (96.4%) of the complete core eukaryotic BUSCO genes (Supplementary Table S6). Of the PCGs, 92.6% could be annotated by at least one of the protein-related databases (Supplementary Table S8). In addition, 1,243 tRNAs, 2,591 rRNAs, and 4,429 other non-coding RNAs were predicted (Supplementary Table S9).

We identified 598,516 repeat elements (198.8 Mb, 49.7%) in subgenome A (*P. alba*) and 603,658 repeat elements (211.6 Mb, 50.8%) in subgenome G (*P. tremula* var. *glandulosa*) based on a strategy that integrates homology and structural evidence (Supplementary Table S10). TEs accounted for the majority of repeat elements, approximately 89.4%. Class I (retrotransposons) and Class II (DNA transposons) TEs accounted for 23.5% and 21.5% of the total, respectively (Supplementary Table S10). The *Gypsy* superfamily accounted for the highest portion (∼48.8%) of TE (Supplementary Table S10).

In addition, transcription factor (TF) genes (subgenome A: 2,017; subgenome G: 1,992) were predicted and classified into 58 gene families (Supplementary Table S11). Among them, MYB (v-Myb avian myeloblastosis viral oncogene homolog) (subgenome A: 172; subgenome G: 169), bHLH (basic-helix-loop-helix proteins) (subgenome A: 163; subgenome G: 162), and ERF (ethylene responsive factor) (subgenome A: 155; subgenome G: 156) were the most abundant.

### Distribution of rDNA and conserved rDNA cluster

We identified a total of 2,591 rDNAs in the “84K” genome, including 2,133 of 5S, 37 of 5.8S, 181 of 18S, and 240 of 28S type. Among them, 5S rDNAs were the most abundant and distributed across 4 chromosomes (chr02G, chr03G, chr17A, and chr17G) forming highly repetitive tandem clusters (Supplementary Data Set 3), with particularly pronounced long clusters within homologous pairs of chromosomes 17 (chr17A and chr17G) (Supplementary Figs. S10 and S11). The 2 homologous chromosomes of chr14 contained 45S (including 5.8S, 18S, and 28S) rDNA clusters concentrated within the telomeric regions (Supplementary Fig. S11). To further investigate the distribution of 5S and 45S rDNAs in Salicaceae, we also identified the rDNAs of 4 willows (*Salix brachista*, *S. dunnii*, *S. purpurea*, and *S. suchowensis*) and 4 poplars (*P. ilicifolia*, *P. simonii*, *P. deltoides*, and *P. trichocarpa*). Interestingly, 5S rDNA was found to be distributed mainly on chromosome 17 in the poplars and on chromosomes 13 and 19 in the willows except for *S. dunnii* (also mainly on chromosome 17 as in poplars) (Supplementary Fig. S11 and Supplementary Data Set 3).

### Phylogenetics and genome comparison

A phylogenetic tree was constructed for 12 Salicaceae species genomes and our subgenomes A and G, using 2,106 single-copy putative orthologs (Supplementary Fig. S12A and Supplementary Table S12). A total of 34,111 gene families were identified, of which 937 and 1,043 were found expanded and 3,315 and 3,491 contracted in subgenomes A and G, respectively (Supplementary Fig. S12A and Supplementary Table S12). When we compared the 2 subgenomes, we found evidence of functional differences in the expanded genes. For example, jasmonic acid and phototransduction associated gene ontology (GO) terms were enriched in subgenome A (Supplementary Fig. S12B and Supplementary Data Set 4), whereas cGMP signaling pathways were enriched in subgenome G (Supplementary Fig. S12C and Supplementary Data Set 5).

**Figure 2.**
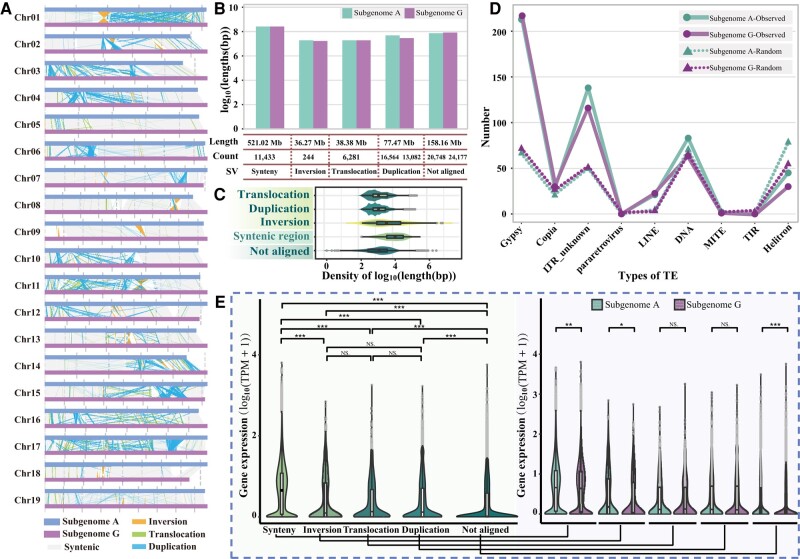
SVs and their effects on gene expression. **A)** SVs between the parental genomes (“G” for the gap-free assembly of *P. tremula* var. *glandulosa* and “A” for the assembly of *P. alba*) with subgenome A as the reference. **B)** Length and count statistics of SVs between parental genomes. Length represents the sum of the lengths of different types of structural variation between the 2 parental genomes. Counts indicate the number of different types of SV on each of the 2 parental genomes. **C)** Length distributions of SVs between the parental genomes. **D)** Statistics on the number of inversion breakpoints (150 bp of each breakpoint site) overlapping with TE in both parental genomes. The solid line represents the observed pattern, and the dashed line represents the pattern from randomization. In boxplots, the center line in the box indicates the median value, and the box height indicates the 25th to 75th percentiles of the total data. Whiskers indicate the 1.5× interquartile range. Points outside the whiskers indicate outliers. **E)** Different types of SV and gene expression. Difference of gene expression for genes of different SV regions is shown on the left, and the comparison between 2 parental genomes on each SV category is shown on the right. *Y*-axis indicates the gene expression levels of genes that overlap with the structural variants. Mann-Whitney-Wilcoxon test. *P < 0.05; **P < 0.01; ***P < 0.001; NS, no significant difference. Error bar type is the standard error (SE). The width of each violin represents the density of the data. In boxplots, the center line in the box indicates the median value, and the box height indicates the 25th to 75th percentiles of the total data. Whiskers indicate the 1.5× interquartile range. Points outside the whiskers indicate outliers.

Synteny analysis revealed extensive collinearity and conserved gene order among Salicaceae genomes (Supplementary Fig. S12A). Genome alignment of the 2 subgenomes revealed collinearity of 521 Mb (∼64% of both subgenomes A and G) (Fig. 2, A and B and Supplementary Table S13). Nevertheless, structural and small local genomic variants were revealed between the 2 subgenomes, totaling 152.11 Mb of rearranged regions and 8,224,933 SNPs (Single Nucleotide Polymorphism) (Fig. 2B; Supplementary Fig. S13 and Supplementary Table S13). Inversions were detected ranging in length from a few hundred bp to several Mb, such as the 5.99 Mb inversion on chromosome 1 (between chr01A and chr01G) (Fig. 2, A and C). Large-scale structural variations were well-supported by Hi-C chromatin contacts, indicating that both genome assemblies and identification of SVs were of high accuracy (Supplementary Fig. S6).

*Gypsy*-type TEs were found associated with genomic inversions. By calculating the proximity of inversion regions and their associated breakpoints (150 bp upstream and downstream of the breakpoint) to the nearest TE, we found that *Gypsy* elements substantially overlapped with such regions (Supplementary Fig. S14). A randomization test revealed that breakpoint regions of inversions were more likely to overlap with *Gypsy* and unknown long terminal repeats (LTRs) in both A and G subgenomes (Fig. 2D), indicating the differential association of TE types with inversions.

SVs may affect gene expression and subgenome dominance in gene expression. We found that gene expression was significantly lower in regions of SVs than in the highly syntenic regions (Fig. 2E). In fact, expression differences between subgenomes (subgenome dominance) were found only in regions of inversion among 3 types of SVs (inversion, translocation, and duplication), indicating differential effects of SVs (Fig. 2E).

### DNA methylation

The global mean methylation levels for mCG, mCHG, and mCHH were 48.8%, 21.7%, and 3.3% in subgenome A, and 50.2%, 23.4%, and 3.4% in subgenome G, respectively (Supplementary Table S14). No substantial differences were observed in the distribution of the 3 DNA methylation contexts along homologous chromosomes of the 2 subgenomes (Supplementary Fig. S15, A and B). DNA methylation patterns in the gene body and upstream/downstream regions showed that the methylation levels of mCG, mCHG, and mCHH were not substantial different between the two parental genomes (Supplementary Fig. S15, C to E). However, methylation levels were generally higher in the upstream and downstream regions of genes than in the gene body regions (Supplementary Fig. S15, C to E).

### Allele-specific expression

**Figure 3.**
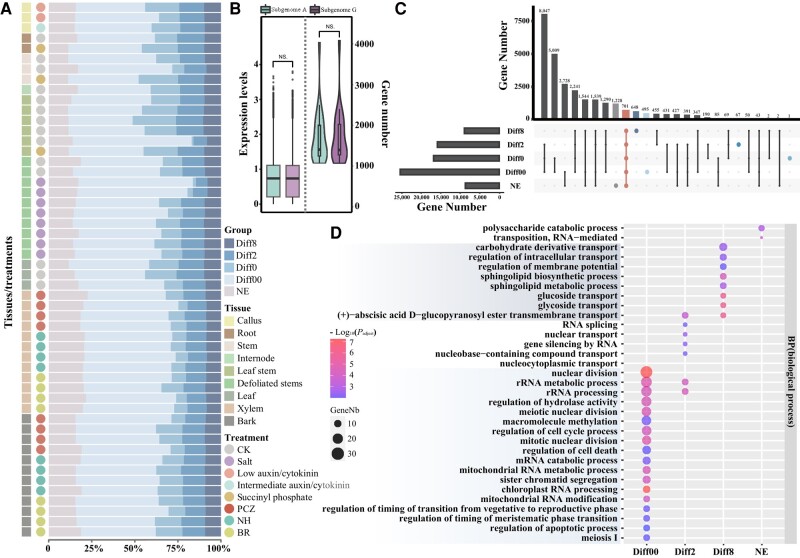
ASE. **A)** Grouping of ASE among samples from different tissues and treatments. Internode (botany), a portion of a plant stem between nodes. BR, brassinosteroid treatment. NH, no hormone treatment. PCZ, propiconazole treatment. **B)** Comparison on gene expression (the left panel) and gene number (the right panel) between 2 parental genomes (“G” for the gap-free assembly of *P. tremula* var. *glandulosa* and “A” for the assembly of *P. alba*). Mann-Whitney-Wilcoxon test. *P < 0.05; **P < 0.01; ***P < 0.001; NS, no significant difference. In boxplots, the center line in the box indicates the median value, and the box height indicates the 25th to 75th percentiles of the total data. Whiskers indicate the 1.5× interquartile range. **C)** UpSet plot for 5 categories of ASE (both alleles were not expressed); Diff00: non-significant difference between a pair of alleles with *P-*adjust > 0.05; Diff0: significant difference between a pair of alleles with *P-*adjust ≤ 0.05 and FC ≤ |2|; Diff2: significant difference between a pair of alleles with *P-*adjust ≤ 0.05 and |2| < FC < |8|; Diff8: significant difference between a pair of alleles with *P-*adjust ≤ 0.05 and FC ≥ |8|. **D)** GO enrichment test of 5 categories of allelic gene expression. The enriched GO terms with corrected *P*-value < 0.05 are presented. The color of circles represents the statistical significance of the enriched GO terms. The size of the circles represents the number of genes in a GO term. “*P*-adjust” is the Benjamini–Hochberg FDR adjusted *P*-value.

A total of 28,030 allele pairs (75.2% of the total number of predicted genes) were identified based on collinearity and homology (Supplementary Fig. S16). The merging of divergent genomes by hybridization or allopolyploidization often biases subgenome expression (Bird et al. 2018; Vohra et al. 2020). In general, we found no evidence of subgenome- or chromosome-level bias in either gene content or gene expression, except for some inverted regions (see above) (Figs. 2E and3B). On the other hand, although the expression of both subgenomes was generally balanced, we observed significant ASE. Based on the expression patterns in different tissues or treatments, we defined the following categories of allelic expression bias: (1) no expression (both alleles were not expressed) (NE); (2) no significant difference between a pair of alleles with *P-*adjust > 0.05 (Diff00), and (3) significant difference between a pair of alleles with *P-*adjust ≤ 0.05, ASE. The ASE group was further divided into different classes based on the fold change (FC) in expression: (1) Diff0, when FC ≤ |2| (Diff0); (2) Diff2, when |2| < FC < |8| (Diff2); and (3) Diff8, when FC ≥ |8| (Diff8) (Fig. 3A and Supplementary Table S15).

Many allele pairs (37.2% to 69.1%) did not show significantly differential expression patterns within the same tissue/treatment, and the proportions of allele pairs with the highest ASE (Diff8) were relatively stable among 8% to 10% of allele pairs (Fig. 3A). Alleles from the lower allelic difference (Diff0) category had significantly higher absolute transcript abundance than those from the medium allelic difference (Diff2) and the highest allelic difference (Diff8) categories (Supplementary Fig. S17). We noted that a given allele pair may be present in different categories among different tissues/treatments but their expression differences were usually found in the immediate allele difference categories (Fig. 3C). We found respectively 1,228, 495, 1, 67, and 648 allele pairs specifically present in categories NE, Diff00, Diff0, Diff2, and Diff8 (Fig. 3C). Such group-specific genes were enriched in various GO terms. In particular, the Diff00 group affiliated genes were significantly enriched (false discovery rate (FDR) < 0.001) in several GO terms of primary functional processes such as cell division and meiosis, whereas the Diff8 group genes were enriched in GO terms related to plant resistance such as those of secondary metabolic biosynthesis and transport (Fig. 3D and Supplementary Fig. S18).

### ASE modeling and key features

**Figure 4.**
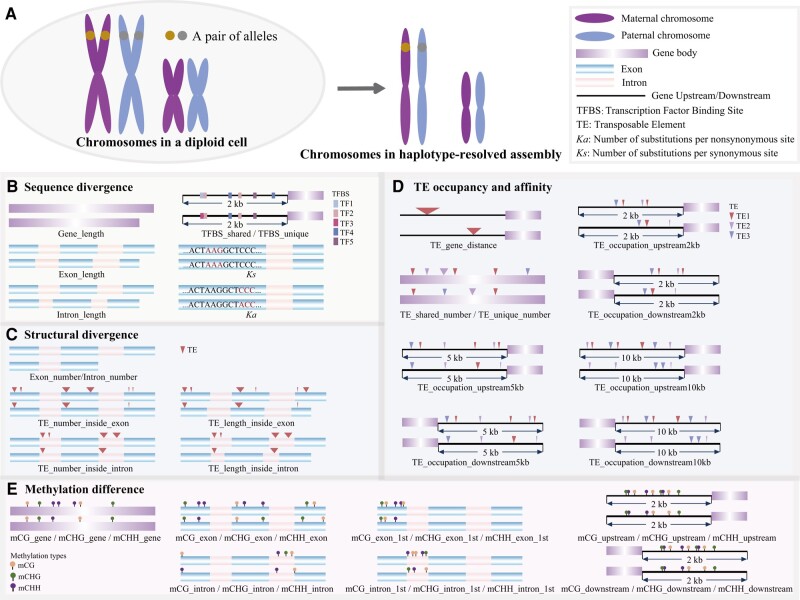
Alleles in haplotype-resolved genome assembly and origins of (epi-)genetic features used from machine-learning modeling. **A)** Schematic chromosomes showing a pair of alleles in a diploid cell and the haplotype-resolved genome assembly. **B)** Features of sequence divergence between a pair of alleles. **C)** Features of structural divergence between a pair of alleles. **D)** Features of difference in TE occupancy and affinity of gene upstream and downstream regions between a pair of alleles. **E)** Features of methylation difference in upstream, downstream, gene body, exon, and intron regions between a pair of alleles.

**Figure 5.**
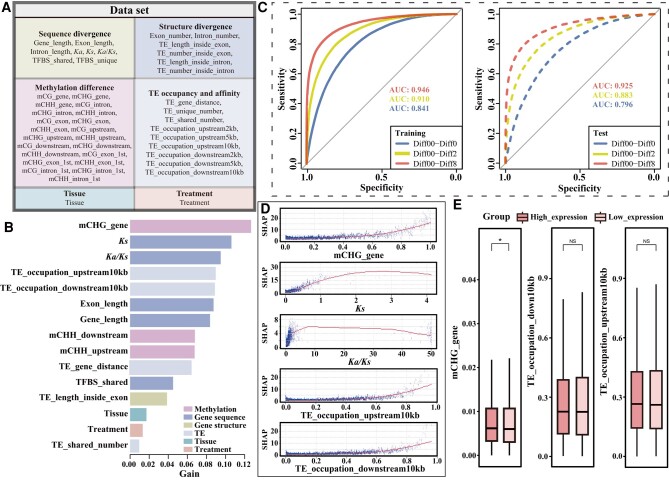
Machine-learning modeling of ASE and the key factors. **A)** All features used in machine-learning modeling. **B)** Ranking of the 15 top features in the XGBoost model (Model 1). Model 1: A XGBoost classification model with 15 predictors (features) and 1 response with 4 groups (Diff00, Diff0, Diff2, and Diff8). Diff00: non-significant difference between a pair of alleles with *P-*adjust > 0.05; Diff0: significant difference between a pair of alleles with *P-*adjust ≤ 0.05 and FC ≤ |2|; Diff2: significant difference between a pair of alleles with *P-*adjust ≤ 0.05 and |2| < FC < |8|; Diff8: significant difference between a pair of alleles with *P-*adjust ≤ 0.05 and FC ≥ |8|. **C)** ROC curves and AUC values of the XGBoost model (Model 1). **D)** SHAP summary plots of the top 5 features in the XGBoost model (Model 1). Each blue dot represents an observation. SHAP: SHapley Additive exPlanations. **E)** Absolute TPM expression abundance for high and low expression allelic genes in an allele pair. Mann-Whitney-Wilcoxon test. *P < 0.05; **P < 0.01; ***P < 0.001. NS, no significant difference. In boxplots, the center line in the box indicates the median value, and the box height indicates the 25th to 75th percentiles of the total data. Whiskers indicate the 1.5× interquartile range.

The eXtreme gradient boosting (XGBoost) approach was developed as a supervised machine-learning method (Chen and Guestrin 2016). The XGBoost model not only has high accuracy in classification models, but also performs excellently when dealing with tabular data problems (Zamani Joharestani et al. 2019). We calculated 46 genetic and epigenetic features of the 6 categories including methylation difference, sequence divergence, structural divergence of an allele pair, TE occupancy and affinity, tissue and treatment expression (see Materials and Methods, Figs. 4 and 5A and Supplementary Note S1), and integrated them into XGBoost modeling to predict ASE groups and to determine which of the input features were most informative for ASE prediction.

First, we created an XGBoost model (Model 0) with all 46 features and obtained the importance ranking of features (Supplementary Fig. S19 and Supplementary Tables S16 and S17). To avoid potential overfitting from correlation among features, we performed pairwise correlation analysis among all features (Supplementary Fig. S20) and filtered out those that were highly correlated. After this thinning step, we retained 15 features for further XGBoost modeling (Fig. 5B). The XGBoost model “Model 1” was built to predict the 4 ASE groups (Diff00, Diff0, Diff2, and Diff8) with the selected 15 features: y ∼mCHG_gene + *Ks* + *Ka/Ks* + TE_occupation_upstream10kb +TE_occupation_downstream10kb + Exon_length + Gene_length + mCHH_downstream +mCHH_upstream + TE_gene_distance + TFBS_shared + TE _length_inside_exon + Tissue +Treatment + TE_shared_number. This model revealed that the most important features predictive of ASE were related to DNA methylation, with CHG gene body methylation being the most important feature, followed by gene sequence divergence (i.e. *Ks*, *Ka*/*Ks*) (Fig. 5B and Supplementary Table S18). TE occupancy (TE_occupation_upstream10kb, TE_occupation_downstream10kb) was ranked next after sequence divergence features *(Ks*, *Ka*/*Ks*) in Model 1 (Fig. 5B). Some features of the structural divergence categories, i.e. length of exons and genes, were also important (Fig. 5B). The model also showed that CHH methylation within the 2 kb upstream (promoter region) and 2 kb downstream regions of genes also played an important role in the categories of DNA methylation (Fig. 5B). TE affinity (TE_gene_distance, distance between an allele to adjacent TE) was ranked following the methylation divergence features (Fig. 5B). The transcription factor binding site (TFBS) composition feature (TFBS_shared) of the promoter region was ranked lower following TE affinity. The effects of different tissues and treatments were ranked following the structure divergence features (Fig. 5B).

To further clarify the relationship between different potentially influencing factors (various genetic and epigenetic features) and ASE, SHapley Additive exPlanations (SHAP) was used for model interpretation. The SHAP dependency analysis was performed to obtain and define the 5 most important features (mCHG_gene, *Ks*, *Ka*/*Ks*, TE_occupation_upstream10kb, and TE_occupation_downstream10kb) influencing ASE (Fig. 5D). This method allowed us to evaluate the importance of the features for the model output, and to extract the complex nonlinear effects between the response (ASE) and the predictors (features), which in turn indicated the complexity of the ASE dependency. Examining the SHAP results, we found that the top 5 features all showed a trend of gradually increasing SHAP values as the value of each feature increased (Fig. 5D); briefly, the greater the expression fold difference between the alleles, the greater the difference in methylation frequency of the gene body CHG between that pair of alleles; similar results were obtained between a pair of alleles for the difference in the occupancy by TE in regions 10 kb upstream and 10 kb downstream of the gene (Fig. 5D). We also found that the greater the allelic *Ka/Ks* values, the greater the difference in allelic expression (Fig. 5D). Regarding the relationship between a specific gene and ASE, we found that in 1 allelic pair that the allele with an on average high methylation pattern of that gene body had higher expression, although the overall differences were minimal (Fig. 5E). However, the occupancy by TE in the upstream 10 kb region showed no significant difference for the allele pair with high or low expression, respectively (Fig. 5E), indicating a possible complex relationship.

To demonstrate the predictability of ASE and the reliability of the results, we constructed 2 additional XGBoost models, including a classification model with 1 response of 2 groups (group 1 with: Diff0, Diff2, and Diff8; group 2 with: Diff00) and 15 predictors (features) (Model 2) and a regression model with 1 response of the expression difference [in transcripts per million (TPM)] and 15 predictors (Model 3). Remarkably, the importance rankings for the 15 features used in all 3 models (Models 1, 2, and 3) did not differ substantially, and the models exhibited low overfitting and consistently high accuracy, indicating good predictability of ASE (Supplementary Figs. S21 and S22 and Supplementary Tables S18 to S20). Both, receiver operating characteristic curves (ROC) and area under the curve (AUC) values confirmed the good performance of Models 1 and 2 (Fig. 5C and Supplementary Fig. S21B). The accuracies of Models 1 and 2 were 77% and 81% on the training set, and 74% and 78% on the test set, respectively (Supplementary Table S19). Moreover, especially in Model 2, the values of mean F1 and mean sensitivity were 77% and 78% on the training set and 74% and 75% on the test set, respectively (Supplementary Table S19). In addition, the ranking of feature importance in the XGBoost regression model (Model 3) was essentially the same as for the classification models (Models 1 and 2), and no overfitting was observed (Supplementary Fig. S22B and Supplementary Table S20).

## Discussion

A better understanding of ASE in hybrid genomes is insightful for comprehending genomic interactions that lead to hybrid vigor or hybrid depression. In the present study, we obtained a telomere-to-telomere (T2T) gapless assembly for one and a nearly gapless assembly for the other parental genome of the F1 poplar hybrid clone “84K” using a combination of long-reads sequencing (HiFi and ONT) and a trio-binning design. The final size of the assembled genome was 817 Mb (subgenome A: 400.2 Mb and subgenome G: 416.8 Mb), which is larger than the 2 previously published assemblies (Qiu et al. 2019; Huang et al. 2021) and also larger than a recently published genome assembly for another hybrid poplar clone (INRA 717-1B4) (Pt HAP1: 394.3 Mb, Pa HAP2: 402.6 Mb) (Zhou et al. 2023). Compared with the 2 previous versions, the total contiguity (22.4 Mb of contig N50) of the present assembly increased by 9-fold (Qiu et al. 2019; Huang et al. 2021) and 5-fold (Qiu et al. 2019; Huang et al. 2021), respectively. The total number of predicted protein-coding genes (66,336) was lower compared to the previous 2 genome assemblies (72,574 and 77,150), likely due to genes that were fragmented across scaffolds in the previous assemblies. Additionally, TEs accounted for 44.9% of the present genome assembly compared with 24.4% (184 Mb) and 40.8% (320 Mb) in the 2 previous reports, suggesting that TEs were better resolved in the present assembly. There were only 2 gaps in the whole genome assembly, both located in the repeat-rich flanking regions of the centromere region of chromosome 9 of subgenome A. This assembled genome allowed for the accurate identification of alleles, their divergence in sequence and methylation, and clearer partitioning of haploid-specific reads from RNA sequencing, thus enabling for extraction of multiple genetic and epigenetic features and the machine-learning modeling of ASE.

Based on our high-quality genome assembly, we detected regions of collinearity between the 2 parental genomes spanning almost the entire genome. Extensive synteny between the 22 haplotypes was also shown in the recently published genome of hybrid aspen *P. tremula* × *P. alba* INRA 717-1B4, in which the syntenic regions make up about 79.7% to 83.0% of each haplotype (Zhou et al. 2023). Low *Ka* and *Ks* values (<0.05) were observed for most alleles, indicating strong identity between alleles of genes. Large inversions between the two parental genomes were observed and occurred frequently around the centromeric regions of chromosomes (Fig. 1A), then the presence of this structural variation was also verified in the Hi-C chromatic contact map (Supplementary Fig. S6). This pattern was also observed in patchouli (*Pogostemon cablin*) (Shen et al. 2022). We found that structural variation can potentially affect gene expression in such structurally variable regions, and gene expression was substantially lower than in syntenic regions. Subgenome bias of gene expression was found only in inversion regions among the different types of SV, suggesting differential effects of SVs on gene expression. TEs were scattered among homologous regions at distant genomic locations, resulting in large-scale deletions, duplications, and inversions (Bourque et al. 2018). Therefore, SVs detected in this study were likely associated with the insertion or removal of TEs. In addition, we found a signal for a close association between the inversion region and methylation, with relatively high levels of CHG methylation in the inversion region. We hypothesize that the accumulation of TEs may lead to chromosome inversions, and that these regions promoted methylation modifications, which in turn affected gene expression levels to some extent.

A pattern of allelic expression bias was observed in oilseed rape (*Brassica napus*) (Yoo et al. 2013; Chalhoub et al. 2014), furthermore a pattern of local allelic expression bias was also observed in cotton (*Gossypium* spp.) (Yoo et al. 2013), whereby dominance of global subgenome expression was not biased toward a specific subgenome. Similarly, we did not find evidence of global subgenomic dominance in the hybrid poplar “84K.” From our analysis, most alleles were coordinately expressed, but 34.7% of the alleles exhibited expression bias (>2-fold change in expression) in different tissues or treatments. Similar results were observed in Tieguanyin tea (*Camellia synensis*) leaves, where 30.1% of genes exhibited substantial ASE (Zhang et al. 2021b), indicating local expression bias in allele expression patterns. Additionally, genes exhibiting greater expression variance identified in willow hybrids were found to be enriched for GO terms such as “response to stimulus.” These genes may play a crucial role in heterosis (Carlson et al. 2022). Accordingly, we found that genes of the same functional category also showed different expression levels between alleles across different tissues and treatments (Fig. 3C). It is reasonable to assume that hybrids may utilize 1 of the alleles more under different conditions due to their differential regulation, so the accumulation of such differential beneficial effects of the 2 alleles in hybrids is likely an important cause of heterosis (Shao et al. 2019). Specific functions are often attributed to allele-specific expression of genes (ASEGs). In this study, for example, alleles from the highest allele difference category (Diff8, see Results) were more enriched in GO terms related to resistance, such as secondary metabolic biosynthesis and ion transport. Genes belonging to the Diff00 (without significant difference in expression) group, were significantly enriched in several GO terms for primary functional processes such as cell division and meiosis. These ASEGs provide a list of genes that should be pursued in the future to advance our understanding of the genetic and molecular mechanisms underlying heterosis.

ASE can be influenced by numerous factors, which act in concert in an intricate regulatory network to regulate ASE, but identification of these factors and their impact has been challenging. Here, we utilized an ML approach to identify the contributing factors to ASE. We found that methylation, TE occupancy, and allele sequence differences were important factors that were highly predictive of ASE. In our ML models, DNA methylation showed the largest contribution (Fig. 5B), especially mCHG. Highly conserved epigenetic modification is an important means of regulating gene expression, contributing to hybrid vigor (Matsunaga et al. 2022). DNA methylation normally suppresses gene expression (Mao et al. 2015). However, a few studies have also found a positive role of DNA methylation in gene expression, e.g. in *Arabidopsis* (Lei et al. 2015), rice (*O. sativa*) (Xu et al. 2020a), and sunflower (*Helianthus annuus*) (Niu et al. 2022). In this study, we found that alleles with high methylation did not always have low expression, which is consistent with what has already been shown for methylation-regulated gene expression. We also found that both upstream and downstream TE occupation could affect the level of gene expression, and they were of similar importance (Fig. 5B). The true relationship between TE occupancy and ASE is more complex than had anticipated. TEs can carry regulatory sequences, such as promoters or enhancers, which can influence the expression of nearby genes. TEs can cause insertions, deletions, inversions, and other structural variations in the genome. These events can lead to changes in gene copy number or disrupt regulatory elements, potentially causing ASE. The exact mechanism by which TEs influence ASE can be highly context-dependent, varying with the type of TE, its location in the genome, the gene in question, and the broader genomic and cellular environment. As a result of this complexity, it is often challenging to predict precisely how TEs will affect ASE without detailed empirical studies. All in all, in hybrids, this phenomenon (ASE) appears to allow for better utilization of the favorable copies of the parental genes by specifically expressing them while limiting the expression levels of less or unfavorable copies, consistent with the genetic definition of dominance. Similarly, by using regression and machine-learning methods, it has been verified in willow trees that the differential expression genes of parents can effectively predict heterosis. This provides theoretical support for hybrid breeding based on genetic expression (Carlson et al. 2022).

Overall, the high-quality genome assembly of the hybrid poplar “84K” provides an important reference and framework for further development of hypotheses on hybrid performance and molecular tools for breeding and precise manipulations with genome editing for functional studies. We illustrate that ASE in a genome can be studied using machine-learning modeling, identifying the key contributor to ASE. This approach enriches the toolkit of functional genomics and is expected to promote further applications of machine-learning in solving complex biological problems.

## Materials and methods

### Plant material and data collection

Fresh leaves for DNA extraction from the hybrid poplar clone “84K” (*P. alba* × *P. tremula* var. *glandulosa*) were collected from a 1-month-old plantlet growing under greenhouse conditions at 20 °C.

Illumina whole genome sequencing data were obtained from the SRA database, which included “84K” (SRR9831374, SRR9831375, SRR9831376, and SRR9831377) and its 2 parents [*P. alba* (SRR11587873) and *P. tremula* var*. glandulosa* (SRR11853653)]. These data, “84K”, *P. alba*, and *P. tremula* var. *glandulosa* had 50, 31, and 47 Gbp of raw reads, respectively. We obtained 374 Gbp of RNA-sequencing data for the “84K” clone from the SRA database, representing 156 samples from different tissues and different treatments (Supplementary Table S2 and Supplementary Data Set 1).

### Library construction and sequencing

For HiFi sequencing, single-molecule real-time circular consensus sequencing (CCS) library preparation was performed. Briefly, high-quality genomic DNA was extracted from the “84K” leaves, and sheared and purified. Sequencing libraries were prepared and subjected to fragment size selection (fragment molecules ≥ 11 kb) prior to HiFi sequencing on the PacBio Sequel II platform. The generated libraries were sequenced on 3 SMRT (Single Molecule Real-Time Sequencing) cells.

For ONT sequencing, leaves were collected to extract high-quality genomic DNA using the cetyltrimethylammonium bromide method (Doyle and Doyle 1987). After purification and quantification, adaptors were ligated. Then, sequencing libraries with ∼20 kb DNA inserts were prepared and sequenced on 2 flow cells on the Nanopore PromethION platform.

For Hi-C library preparation, we followed a standard procedure (Xie et al. 2015). The cross-linked DNA was digested, linked, purified, cut, and biotinylated with MboI restriction enzyme. Subsequently, the resulting DNA fragments were subjected to end-repair, adaptor ligation, and polymerase chain reaction, and paired-end sequencing libraries were constructed. These libraries were then sequenced using an Illumina Novaseq 6000 machine with 350 bp fragment.

Additional details are provided in Supplementary Note S2.

### Genome assembly and quality assessment

De novo genome assembly involved the following: primary assembly, Hi-C scaffolding, gap-filling, and optimization (Supplementary Fig. S2). First, HiFi reads obtained by sequencing in PacBio CCS mode were directly assembled with hifiasm v0.13-r308 (Cheng et al. 2021). We used a haplotype-switching resolution method, trio-binning which used short-reads generated from parents to bin long reads generated from offspring prior to assembly (Koren et al. 2018). The method has been recommended for development of telomere-to-telomere (T2T) or gapless genome assembly efforts (Nurk et al. 2022). We then used purge_dups v1.2.5 (Guan et al. 2020) and performed manual controls. The assembly described above yielded the v0.1 version of the “84K” genome (Supplementary Table S3). The Hi-C reads were mapped to the v0.1 genome assembly using Juicer v1.7.6 (Durand et al. 2016).

We used an automated process to correct and order orientation errors by using the 3D-DNA scaffolding pipeline (Dudchenko et al. 2017). Juicebox v11.08 (Durand et al. 2016) was used to fine-tune the assembled scaffolds in a graphical and inter-active fashion through manual adjustments. To further improve the accuracy of the assembly, each chromosome was individually re-scaffolded with 3D-DNA and manually adjusted using Juicebox. Gaps were identified and their length was set to 100 bp. Then, the gaps were filled with ONT contigs or ONT ultra-long reads. The ONT ultra-long reads were further used for the gap filling using tgs-gapcloser v1.1.1 (Xu et al. 2020b), and the 38 chromosomes with only 2 gaps were obtained. Finally, we further optimized the genome assembly using NextPolish v1.1.0 (Hu et al. 2020) and Redundans v0.14a (Pryszcz and Gabaldon 2016). In addition, the chloroplast genome (Pt) was assembled using GetOrganelle v1.6.0 (Jin et al. 2020), and the complete cyclic molecule was assembled with SMARTdenovo (Liu et al. 2021a).

The accuracy and structural completeness of the genome assembly were assessed by the ratio of genome collinearity with other *Populus* species using minimap2. BUSCO v2.0.1 (Simao et al. 2015) was used to assess genome completeness. In addition, PacBio long reads and Illumina reads were mapped to the genome assembly using Minimap2 v2.17 (Li 2018) and bwa v0.7.17 (Li 2013), respectively. Additionally, the transcriptome assembled in the current study was also mapped to the genome assembly using HiSat2 v2.1.0 (Kim et al. 2015).

Additional details are provided in Supplementary Note S3.

### Centromere and telomere identification

As centromeric regions are rich in repeats, *k*-mers (*k* = 17) were counted from the Illumina reads using jellyfish, and then all *k*-mers with frequency ≥5,000 were assembled into *k*-mer graphs. The loop (*k*-mer graphs) in them was then searched and disentangled into a linear sequence, which were assumed to be motifs for high-frequency tandem repeat. Subsequently, all these tandem repeat motifs were compared to the genome using RepeatMasker v4.0.7 to identify genomic regions with a high occurrence of such motifs and to infer possible centromere regions.

We locally re-assembled and patched telomeric sequences for the 38 chromosomes of “84K.” First, we aligned all HiFi reads to the genome using minimap2 (v2.17, minimap2 -reference genome -reads -ax map-hifi) (Li 2018) and for each telomere, we collected all reads within 100,000 bp of the chromosome terminus. Then, these reads were assembled and the assembled contigs were mapped to the genome using hifiasm v0.13-r308 (Cheng et al. 2021) (unimap -x asm5). If the contigs could provide extensions for the chromosome, then the extensions were included in the assembled chromosome. A total of 7 telomere regions were extended using this strategy, with extension lengths ranging from 100 to 2,700 bp. Finally, 38 chromosome termini were analyzed for the presence of telomeric signature sequences (TTTAGGG)n.

### Transcriptome assembly

Using the obtained Illumina RNA-Seq data, we used 3 strategies to assemble the transcriptomes, including de novo assembly using Trinity v2.13.2 (Grabherr et al. 2011), reference genome-guided assembly using Trinity and HiSat2 v2.1.0 (Kim et al. 2015), and genome-guided assembly using StingTie v2.2.0 (Pertea et al. 2015) with read mapping performed using HISAT2 v2.1.0. The 3 sets of transcriptomes were combined and redundancy was removed using CD-HIT v4.6 (Fu et al. 2012) with 95% identity and 95% coverage (Supplementary Table S21).

### Gene prediction and functional annotation

First, protein sequences extracted from *Arabidopsis thaliana* and 17 Salicaceae species were merged, followed by redundancy removal using CD-HIT v4.6. Subsequently, the assembled transcriptome assembly and the protein sequences were aligned to the repeat-masked reference genome assembly using BLAST v2.2.28+ (Boratyn et al. 2012), and we further optimized the alignment using Exonerate v2.4.0 (Slater and Birney 2005). The complete genes identified by BUSCO were used for ab initio gene prediction using AUGUSTUS v3.2.3 (Stanke et al. 2008). The gene model prediction using the MAKER v2.31.9 (Cantarel et al. 2008) was finalized using AUGUSTUS v3.2.3 (Stanke et al. 2008). Non-coding RNA (ncRNA) prediction was performed using specific databases and packages, i.e. barrnap v0.9, tRNAscan-SE v2.0 (Lowe and Eddy 1997), and Rfam database (version 9.1). In addition, the genomes of the 2 parent genomes (subgenomes A and G) and 8 Salicaceae species were used for rDNA annotation with barrnap. The distribution of rDNAs was mapped using TBtools v1.098769 (Chen et al. 2020a).

Functional annotation of protein-coding genes was performed using 3 strategies. First, annotation was performed using eggNOG-mapper v2.0.5 (Huerta-Cepas et al. 2017). Second, a sequence similarity search was performed for functional annotation. Third, domain similarity searches were also performed.

Additional details are provided in Supplementary Note S4.

### Repeat annotation

Whole genome repeat annotation was performed using EDTA v1.9.3 (–overwrite 1 –sensitive 1 –anno 1 –evaluate 1) (Ou et al. 2019), a TE annotator that integrates homology- and structure-based approaches to identify TE.

### Phylogenetics and gene collinearity in the Salicaceae

The protein-coding sequences of the 2 parental genomes (subgenomes A and G) and Salicaceae species were used for comparative genomics (Supplementary Table S12). OrthoFinder2 v2.3.12 was used to construct the orthogroups (Emms and Kelly 2019). Based on 2,106 single-copy putative orthologs, we used IQTREE v1.6.7 (Nguyen et al. 2015) to construct a maximum likelihood (ML) phylogenetic tree. MAFFT v7.407 (Katoh and Standley 2013) was used to align homologs before converting the aligned protein sequences to codon alignment. Concatenated amino acid sequences were trimmed using trimAL v1.4 (Capella-Gutierrez et al. 2009). The MCMCTree program from PAML v4.9j (Yang 2007) was used to estimate divergence times under independent substitution rate (clock = 1) setting. CAFÉ v4.2.1 (Han et al. 2013) was then used to infer gene family expansion and contraction based on the chronogram of the 14 species analyzed. Gene collinearity analysis on chromosome level in 8 species and the parental subgenomes A and G was performed using MCScanX (Wang et al. 2012).

Additional details are provided in Supplementary Note S5.

### Variation between the 2 parental genomes

The nucmer alignment tool from the MUMmer toolbox v4.0.0 (Marçais et al. 2018; Zhao et al. 2018) was used to perform whole-genome alignment. Alignment results were filtered by identity (>90) and alignment length (>100). Finally, syntenic regions, structural rearrangements, and sequence differences between the 2 parental genomes were identified using SyRI v1.3 (Goel et al. 2019). The genome of the *P*. *alba* parent (subgenome A) was used as the reference and subgenome G (from the other parent, *P. tremula* var. *glandulosa*) as the query.

The distance between the inversion region on the chromosome and the nearest TE was also calculated with BEDtools v2.29.2 (Quinlan 2014). Here, TEs include both randomly generated and observed TEs. We calculated the average expression (in TPM) for each gene in the syntenic and the different SV regions across all samples to represent the gene expression levels. The two-sided Wilcoxon test was used to determine the significant differences in gene expression levels.

Additional details are provided in Supplementary Note S6.

### Identification of allelic genes between parental genomes

We used GeneTribe V1.1.0 (Chen et al. 2020c) with default parameters to identify candidate allele pairs between homologous chromosomes. Further, we manually checked the GeneTribe output and removed allele pair candidates found in noncollinear regions.

### RNA-Seq data and allelic gene expression

We collected a total of 156 RNA-seq samples of the “84K” clone, with 3 independent biological replicates for each treatment. Low-quality reads and adapters were removed and the remaining reads were quantified for gene expression estimation using Salmon v1.6.0 (Patro et al. 2017), through which we obtained count values and normalized TPM values for each sample (Supplementary Data Set 2). If the TPM expression value of a gene/allele in TPM exceeded 0.5 in any sample, we considered it to be expressed. Differentially expressed alleles were identified using the DESeq2 package (Love et al. 2014). The following ranges of FC were used as criteria to determine differential expression: no expression, Diff00, Diff0, Diff2, and Diff8.

Additional details are provided in Supplementary Note S7.

### Identification of TF and TFBS

Protein-coding genes were submitted to PlantRegMap (Tian et al. 2020) to identify TFs with the best match for *Arabidopsis* TFs. The 2 kb sequence upstream of the gene was used to identify TFBSs present in the promoters of the genes. The FIMO tool from the MEME suite v4.12 (Bailey et al. 2009) was used with a position weight matrix obtained from PlantRegMap to predict TFBS based on a set of manually curated, nonredundant, and high-quality TF binding motifs derived from experiments (*P* < 1e^−05^, -motif-pseudo of 1e^−08^, and a -max-stored-scores of 1e^6^). Ultimately, these results were merged as input data needed for machine-learning model construction (see below).

### DNA methylation quantification from ONT long reads

To quantify DNA methylation (CG, CHG, and CHH) of the “84K” clone, we used DeepSignal-plant v.0.1.4 (Ni et al. 2021). In total, we used 2 replicates of 20× raw ONT read data. First, the raw nanopore was preprocessed by conversion to base sequences using Guppy v5.0.16. The signal data (fast5 format) can be successfully converted into base sequences (fastq format). Then, tombo v1.5.1 (Stoiber et al. 2016) was used to manipulate re-squiggle (raw signal genomic alignment). Once the data were processed, methylations contexts were called using DeepSignal-plant under the default reference models. Then, the methylation frequencies of CG, CHG, and CHH sites were generated separately using scripts in the DeepSignal-plant pipeline (Supplementary Table S14).

At least 5 reads covering each cytosine methylation site were retained. To map the distribution of methylation levels along chromosomes, a 500 kb sliding window with a step size of 100 kb was defined using the makewindows function in BEDtools v2.29.2 (Quinlan 2014), and the average methylation level within the window was calculated using methyGff in BatMeth2 (Lim et al. 2012). In addition, the gene body and 2 kb upstream and downstream regions were divided into 100 bins each.

Additional details are provided in Supplementary Note S8.

### Feature extraction for machine-learning modeling

Each column of the feature dataset represented a feature, and each row represented a pair of alleles in comparison. A total of 46 features were created in 6 categories (Supplementary Table S16 and Supplementary Note S1). The feature categories were described as follows: (1) Methylation features, including the difference of a pair of alleles in the average methylation frequency of the gene body, exons, introns, sequences from upstream 2 kb, sequences of downstream 2 kb, first exon, and first intron. (2) TE occupancy and affinity, a factor of great interest in the study of gene expression. This category includes the distance difference of the closest TE insertion to an allele pair, the number of TE insertion shared by an allele pair, the number of unique TE insertion in an allele pair, the difference of TE occupancy in upstream 2/5/10 kb of an allele pair, and the difference of TE occupancy in downstream 2/5/10 kb of an allele pair. (3) Sequence divergence in an allele pair, including that of gene length, exon length, intron length, *Ka*, *Ks*, *Ka*/*Ks*, the number of TFBS shared in the upstream 2 kb between alleles, the number of TFBS unique in the upstream 2 kb between alleles. (4) Structural divergence in a pair of allele, including the number of exon, the intron number, the number of exons with TE insertion, the length of exon with TE insertion, the number of introns with TE insertion, and the length of intron with TE insertion. (5) Tissue, the tissue from which the RNA-seq was done. (6) Treatment, the plant's treatment from which the RNA-seq was done.

Additional details are provided in Supplementary Note S9.

### Model construction

Our XGBoost modeling was implemented with R package, xgboost (Chen et al. 2015). First, XGBoost modeling was performed on a dataset containing 46 features (predictor variables) with the following settings, η = 0.3, γ = 0.001, max_depth = 2, nrounds = 100,000, print_every_*n* = 100, early_stopping_rounds = 200, and default values for other parameters. Our dataset contained 1,220,274 cases, 70% of which were used for training and 30% for testing. Also, we used the same dataset for correlation analysis between features. We performed correlation analysis (Pearson correlation, two-sided test) using the “cor” function in R and kept the features with significant correlation of less than 0.001 (*P <* 0.001, Fisher's Z transform), when we did feature selection for the following Model 1, Model 2, and Model 3. In addition, of the interrelated features, we retained the one that ranked highest in model importance (Supplementary Fig. S19 and Supplementary Table S17).

As a primary step, XGBoost modeling (Model 0) was performed with all 46 features as predictors to predict 4 groups of ASEs (as defined above), which was used to rank the features. After feature selection, an XGBoost model (Model 1) was constructed to predict the 4 groups of ASEs with 15 selected features. In addition, another XGBoost classification model (Model 2) was created to predict 2 ASE groups (no ASE; ASE). Another XGBoost regression model (Model 3) was built to predict the difference in expression (in TPM) of ASE.

To assess the predictability of each classification model, we calculated ROC curves and AUC values (Robin et al. 2011). In addition, the modeling results of this purely data-driven approach could be explained using SHAP to better interpret the model (default values for parameters) (Lundberg and Lee 2017). Here, we used SHAP to explain the influence of the 5 highest-ranking features on the final prediction of ASE.

Additional details are provided in Supplementary Note S10.

### Calculation of *Ka*, *Ks,* and *Ka*/*Ks*

The *Ka* (number of substitutions per nonsynonymous site), *Ks* (number of substitutions per synonymous site), and *Ka*/*Ks* values were estimated for alleles generated based on the Yang–Nielsen model in KaKs_Calculator v2.0 (Wang et al. 2010) after amino acid alignments were converted to the corresponding codon alignments using PAL2NAL v14.

### Gene ontology enrichment analysis

GO enrichment analysis was performed using the R package clusterProfiler (Yu et al. 2012) for genes from extended gene families in the 2 subgenomes or for differentially expressed alleles (Diff0, Diff2, Diff8), with a *P*-value of 0.05 (Fisher's exact test) and a *q* value of 0.05 (using the Benjamini–Hochberg method to control the FDR).

### Accession numbers

Sequence data from this article can be found in the GenBank/EMBL data libraries under accession numbers PRJNA1025943 and PRJNA1025942.

## Supplementary Material

kiae078_Supplementary_Data

## Data Availability

The whole genome sequencing raw data, genome assemblies, and annotations have been deposited in the Genome Sequence Archive in National Genomics Data Center (https://ngdc.cncb.ac.cn/gwh) under the accession number GWHBJXC00000000 (Bio-Project ID: PRJCA010836). The genome assembly and annotation data for subgenomes A and G have also been deposited in the National Center for Biotechnology Information (NCBI, https://www.ncbi.nlm.nih.gov/) under Biological Project accession numbers PRJNA1025943 and PRJNA1025942, respectively. Scripts used for centromere identification are publicly available at: https://github.com/ShuaiNIEgithub/Centromics. Codes and data for allele identification, allele-specific gene expression, and XGBoost model construction are available on Git-hub (https://github.com/shitianle77/84K_genome) and figshare (https://figshare.com/articles/dataset/Gap-free_genome_assembly_of_hybrid_poplar_84K_/24279211). A computational pipeline for allele identification and allele-specific gene expression with haplotype-resolved diploid genome assembly is available at: https://github.com/shitianle77/Allele_auto.

## Supplementary data

The following materials are available in the online version of this article.

**Supplementary Figure S1.** Images of the sequenced individual (the F_1_ hybrid poplar “84K”).

**Supplementary Figure S2.** The schematic diagram illustrates the overall process of the assembly of the poplar “84K” genome and the data required for the assembly process.

**Supplementary Figure S3.** Putative centromeres (green boxes) are determined based on the distribution of the tandem repeat with the highest frequency.

**Supplementary Figure S4.** Telomere sequences assembled in each chromosome.

**Supplementary Figure S5.** Positions of the two gaps located on chromosome 9A (chr09A).

**Supplementary Figure S6.** Genome-wide analysis of chromatin interactions in the genome based on Hi-C data.

**Supplementary Figure S7.** *K-*mer frequency distribution estimated from (A) Illumina, (B) HiFi, and (C) ONT sequences after filtering and correction at *K*-mer size of 17.

**Supplementary Figure S8.** Collinearity of 2 haplotype genomes of the poplar clone “84K” with that of *P. trichocarpa*.

**Supplementary Figure S9.** Collinearity of 2 haplotype genomes of the current (this study) with published genomes of “84K” (Qiu et al. 2019).

**Supplementary Figure S10.** Distribution of rDNA on chromosomes.

**Supplementary Figure S11.** Distribution of rDNA on chromosomes of Salicaceae species.

**Supplementary Figure S12.** Gene family evolution and collinearity analyses among Salicaceae species.

**Supplementary Figure S13.** Length of structural variation and local sequence differences between the subgenomes A and G (subgenome G for the assembly of *P. tremula* var. *glandulosa* and subgenome A for the assembly of *P. alba*).

**Supplementary Figure S14.** Statistics on overlaps between the inversion regions and different TE types (left panel) and between breakpoint region of inversion and different TE types (right panel) in the 2 subgenomes (G for the assembly of *P. tremula* var. *glandulosa* and A for the assembly of *P. alba*).

**Supplementary Figure S15.** DNA methylation patterns.

**Supplementary Figure S16.** Collinearity of a pair of alleles on 2 parental genomes.

**Supplementary Figure S17.** Absolute TPM expression abundance for Diff00, Diff0, Diff2, and Diff8.

**Supplementary Figure S18.** GO enrichment analysis of 5 categories of allelic expression bias.

**Supplementary Figure S19.** Importance ranking and ROC curves of Model 0 (with 46 predictors/features).

**Supplementary Figure S20.** Pair-wise correlation among 46 predictors (features) used in modeling (Model 0).

**Supplementary Figure S21.** Ranking of the 15 features in the XGBoost model (Model 2) and the model assessment.

**Supplementary Figure S22.** Ranking of the 15 features in the XGBoost model (Model 3) and the model assessment.

**Supplementary Table S1.** Statistics of whole genome sequencing data.

**Supplementary Table S2.** Summary of the Illumina reads for the genome assembly of “84K”.

**Supplementary Table S3.** Statistics of the different versions of genome assembly.

**Supplementary Table S4.** Statistics of the genome quality for the final assembly.

**Supplementary Table S5.** Mapping rates of Illumina reads, HiFi reads, and ONT reads to the present genome assembly of “84K”.

**Supplementary Table S6.** Summary of BUSCO evaluation for genome assembly and gene prediction.

**Supplementary Table S7.** Summary statistics of the gene annotation of the “84K” genome.

**Supplementary Table S8.** Summary of functional annotation of predicted genes.

**Supplementary Table S9.** Summary of the annotated RNA genes.

**Supplementary Table S10.** Summary of the repeat elements annotated in the “84K.”

**Supplementary Table S11.** Annotated TF gene families in the “84K” genome.

**Supplementary Table S12.** Summary of gene family expansion and contraction in the “84K” genome.

**Supplementary Table S13.** Summary of identified SVs between 2 parental genomes.

**Supplementary Table S14.** Summary of the percentage of methylation sites of CG, CHG, and CHH in DNA methylation.

**Supplementary Table S15.** Categories and number of allelic expression biases between 2 parental genomes.

**Supplementary Table S16.** 46 features used in the XGBoost machine-learning modeling of ASE.

**Supplementary Table S17.** Ranking of the 46 features in the XGBoost model (Model 0).

**Supplementary Table S18.** Ranking of the 15 features in the XGBoost models (Model 1, Model 2, and Model 3).

**Supplementary Table S19.** Evaluation of the classification XGBoost models (Model 0, Model 1, and Model 2).

**Supplementary Table S20.** Evaluation of the regression XGBoost model (Model 3).

**Supplementary Table S21.** Statistics of transcriptome assembly by different methods.

**Supplementary Data Set 1.** RNA-Seq data used for gene expression analysis.

**Supplementary Data Set 2.** Statistics of mRNA sequencing data for gene annotation.

**Supplementary Data Set 3.** Summary of the amount of rDNA on different chromosomes among the Salicaceae species and 2 subgenomes (Subgenomes A and G).

**Supplementary Data Set 4.** GO enrichment of the significantly expanded gene families in 1 parental genome (*P. alba* genome, the subgenome A).

**Supplementary Data Set 5.** GO enrichment of the significantly expanded gene families in one parental genome (the *P. tremula* var. *glandulosa* genome, the subgenome G).

**Supplementary Note S1.** 46 features used in the XGBoost machine-learning modeling of ASE.

**Supplementary Note S2.** Library construction and sequencing.

**Supplementary Note S3.** Genome assembly and quality assessment.

**Supplementary Note S4.** Gene prediction and functional annotation.

**Supplementary Note S5.** Phylogenetics and gene collinearity in the Salicaceae.

**Supplementary Note S6.** Variation between the 2 parental genomes.

**Supplementary Note S7.** RNA-seq data and allelic gene expression.

**Supplementary Note S8.** DNA methylation quantification from ONT long reads.

**Supplementary Note S9.** Feature extraction for machine-learning modeling.

**Supplementary Note S10.** Model construction.

## Dive Curated Terms

The following phenotypic, genotypic, and functional terms are of significance to the work described in this paper:

ONT Gramene: oxford nanopore technologies
ONT Araport: oxford nanopore technologies

CHG Gramene: In plants and other organisms, DNA methylation is found in three different sequence contexts: CG (or CpG), CHG or CHH (where H correspond to A, T or C).
CHG Araport: In plants and other organisms, DNA methylation is found in three different sequence contexts: CG (or CpG), CHG or CHH (where H correspond to A, T or C).

TFBS Gramene: Transcription Factor Binding Sites (TFBSs)
TFBS Araport: Transcription Factor Binding Sites (TFBSs)

dna Gramene: Deoxyribonucleic acid
dna Araport: Deoxyribonucleic acid

BUSCO Gramene: Benchmarking Universal Single-Copy Orthologs
BUSCO Araport: Benchmarking Universal Single-Copy Orthologs

AUGUSTUS Gramene: AUGUSTUS is a program that predicts genes in eukaryotic genomic sequences.
AUGUSTUS Araport: AUGUSTUS is a program that predicts genes in eukaryotic genomic sequences.

CCS Gramene: circular consensus sequencing
CCS Araport: circular consensus sequencing

SRA Gramene: Sequence Read Archive
SRA Araport: Sequence Read Archive

TEs Gramene: Transposable elements
TEs Araport: Transposable elements
