# Supplementary material for: High-quality genome assembly enables prediction of allele-specific gene expression in hybrid poplar
Source: Plant Physiol. 2024 Feb 27;195(1):652–70. doi: 10.1093/plphys/kiae078 (PMC11060683; doi:10.1093/plphys/kiae078)
Supplement: kiae078_Supplementary_Data [file kiae078_supplementary_data.zip › Supplemental Data.pdf]

High-quality genome assembly enables prediction of allele-specific gene expression in hybrid poplar

Shi *et al.*

Supplemental data

Supplemental Figures

Supplemental Figure S1. Images of the sequenced individual (the F_1 hybrid poplar "84K").

Supplemental Figure S2. The schematic diagram illustrates the overall process of the assembly of the poplar "84K" genome and the data required for the assembly process.

Supplemental Figure S3. Putative centromeres (green boxes) determined based on distribution of the tandem repeat with the highest frequency.

Supplemental Figure S4. Telomere sequences assembled in each chromosome.

Supplemental Figure S5. Positions of the two gaps located on chromosome 9A (chr09A).

Supplemental Figure S6. Genome-wide analysis of chromatin interactions in the genome based on Hi-C data.

Supplemental Figure S7. *K*-mer frequency distribution estimated from Illumina (A), HiFi (B) and ONT sequences (C) after filtering and correction at *K*-mer size of 17.

Supplemental Figure S8. Collinearity of two haplotype genomes of the poplar clone "84K" with that of *Populus trichocarpa*.

Supplemental Figure S9. Collinearity of two haplotype genomes of the current (this study) with published genomes of "84K" (Qiu et al. 2019).

Supplemental Figure S10. Distribution of rDNA on chromosomes.

Supplemental Figure S11. Distribution of rDNA on chromosomes of Salicaceae species.

Supplemental Figure S12. Gene family evolution and collinearity analyses among Salicaceae species.

Supplemental Figure S13. Length of structural variation and local sequence differences between the subgenomes A and G (subgenome G for the assembly of *P. tremula* var. *glandulosa* and subgenome A for the assembly of *P. alba*).

Supplemental Figure S14. Statistics on overlaps between the inversion regions and different TE types (left panel) and between breakpoint region of inversion and different TE types (right panel) in the two subgenomes (G for the assembly of *P. tremula* var. *glandulosa* and A for the assembly of *P. alba*).

Supplemental Figure S15. DNA methylation patterns.

Supplemental Figure S16. Collinearity of a pair of alleles on two parental genomes.

Supplemental Figure S17. Absolute TPM expression abundance for Diff00, Diff0, Diff2, Diff8.

Supplemental Figure S18. Gene ontology (GO) enrichment analysis of five categories of allelic expression bias.

Supplemental Figure S19. Importance ranking and ROC (receiver operating characteristic) curves of Model 0 (with 46 predictors/features).

Supplemental Figure S20. Pair-wise correlation among 46 predictors (features) used in modeling (Model 0).

Supplemental Figure S21. Ranking of the 15 features in the XGBoost model (Model 2) and the model assessment.

Supplemental Figure S22. Ranking of the 15 features in the XGBoost model (Model 3) and the model assessment.

Supplemental Tables

Supplemental Table S1. Statistics of whole genome sequencing data.

Supplemental Table S2. Summary of the Illumina reads for the genome assembly of "84K".

Supplemental Table S3. Statistics of the different versions of genome assembly.

Supplemental Table S4. Statistics of the genome quality for the final assembly.

Supplemental Table S5. Mapping rates of Illumina reads, HiFi reads, and ONT reads to the present genome assembly of "84K".

Supplemental Table S6. Summary of BUSCO evaluation for genome assembly and gene prediction.

Supplemental Table S7. Summary statistics of the gene annotation of the "84K" genome.

Supplemental Table S8. Summary of functional annotation of predicted genes.

Supplemental Table S9. Summary of the annotated RNA genes.

Supplemental Table S10. Summary of the repeat elements annotated in the "84K".

Supplemental Table S11. Annotated transcription factors (TF) gene families in the "84K" genome.

Supplemental Table S12. Summary of gene family expansion and contraction in the "84K" genome.

Supplemental Table S13. Summary of identified structural variations (SVs) between two parental genomes.

Supplemental Table S14. Summary of the percentage of methylation sites of CG, CHG and CHH in DNA methylation.

Supplemental Table S15. Categories and number of allelic expression biases between two parental genomes.
Supplemental Table S16. 46 features used in the XGBoost machine-learning modeling of allele-specific gene expression (ASE).
Supplemental Table S17. Ranking of the 46 features in the XGBoost model (Model 0).
Supplemental Table S18. Ranking of the 15 features in the XGBoost models (Model 1, Model 2 and Model 3).
Supplemental Table S19. Evaluation of the classification XGBoost models (Model 0, Model 1 and Model 2).
Supplemental Table S20. Evaluation of the regression XGBoost model (Model 3).
Supplemental Table S21. Statistics of transcriptome assembly by different methods.

Supplemental Notes

Supplemental Note S1. 46 features used in the XGBoost machine-learning modeling of allele-specific gene expression (ASE).
Supplemental Note S2. Library construction and sequencing.
Supplemental Note S3. Genome assembly and quality assessment.
Supplemental Note S4. Gene prediction and functional annotation.
Supplemental Note S5. Phylogenetics and gene collinearity in the Salicaceae.
Supplemental Note S6. Variation between the two parental genomes.
Supplemental Note S7. RNA-seq data and allelic gene expression.
Supplemental Note S8. DNA methylation quantification from ONT long reads.
Supplemental Note S9. Feature extraction for machine-learning modeling.
Supplemental Note S10. Model construction.

Supplemental Figures

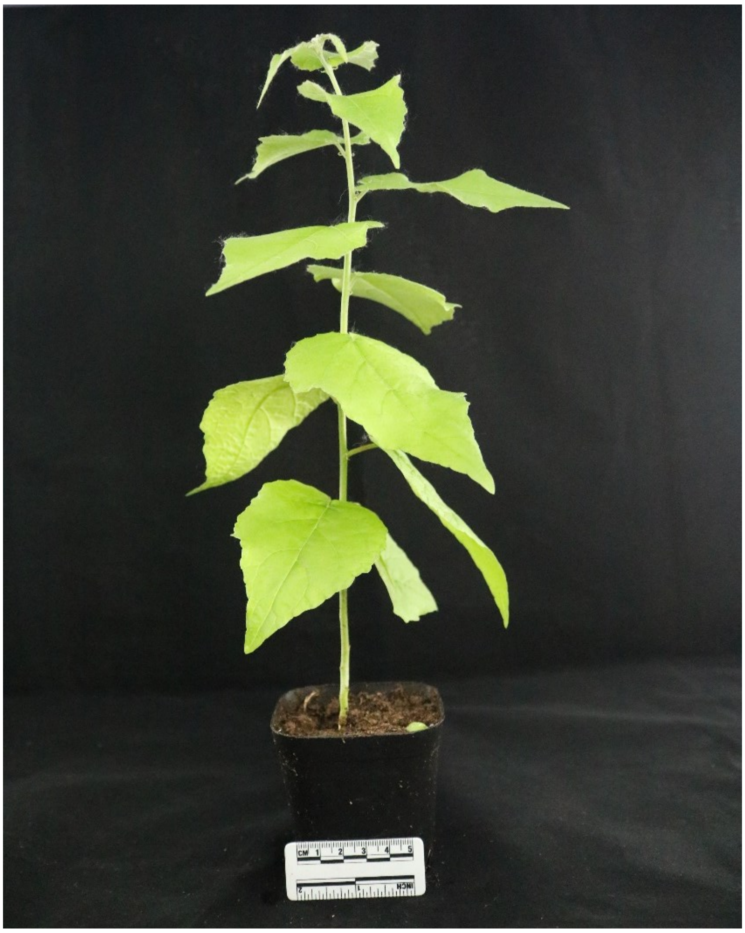

Supplemental Figure S1. Images of the sequenced individual (the F_1 hybrid poplar "84K").

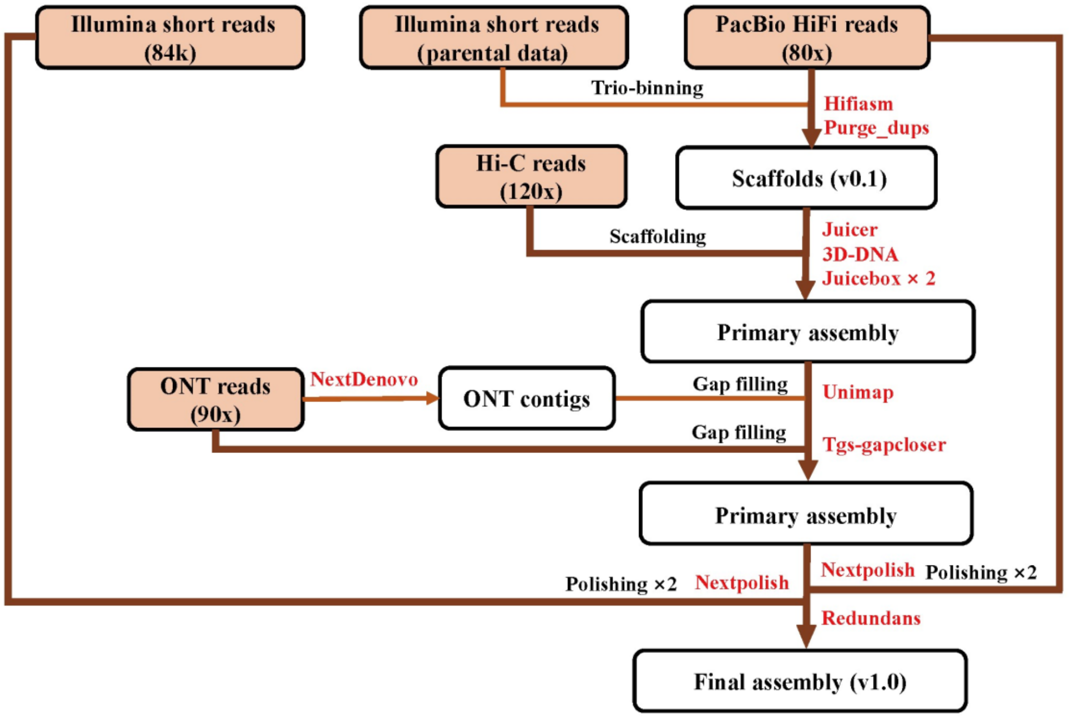

Supplemental Figure S2. The schematic diagram illustrates the overall process of the assembly of the poplar "84K" genome and the data required for the assembly process. Hi-C: High-throughput chromosome conformation capture; ONT: Oxford Nanopore Technology. The red text on the side of the arrow/line in the diagram represents the software, while the black text indicates the purpose of this step. And the orange background represents the input data in this process.

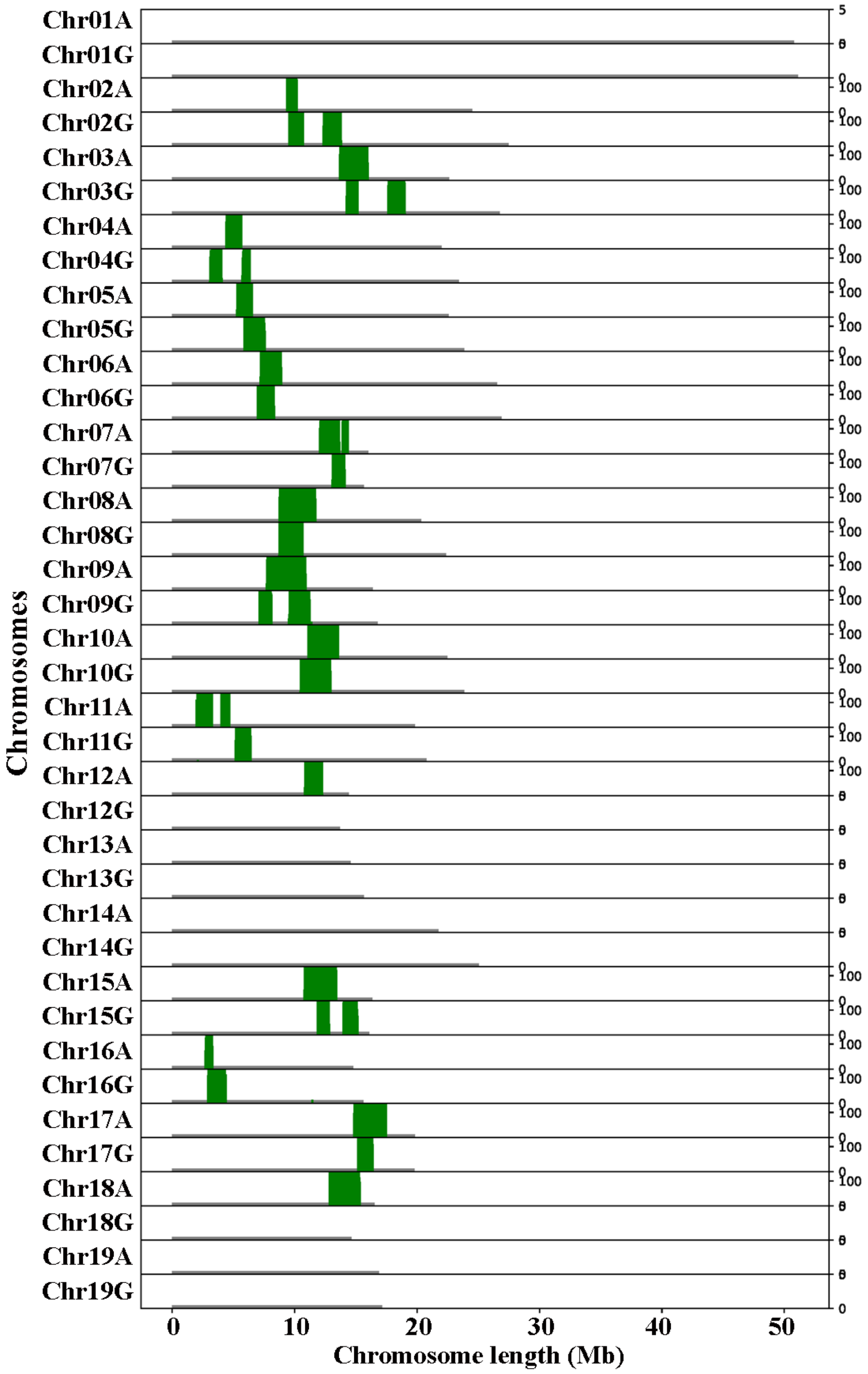

Supplemental Figure S3. Putative centromeres (green boxes) determined based on distribution of the tandem repeat with the highest frequency. The scale on the right indicates the frequency of the tandem repeat with the highest abundance. The length of the black line represents the length of the corresponding chromosome.

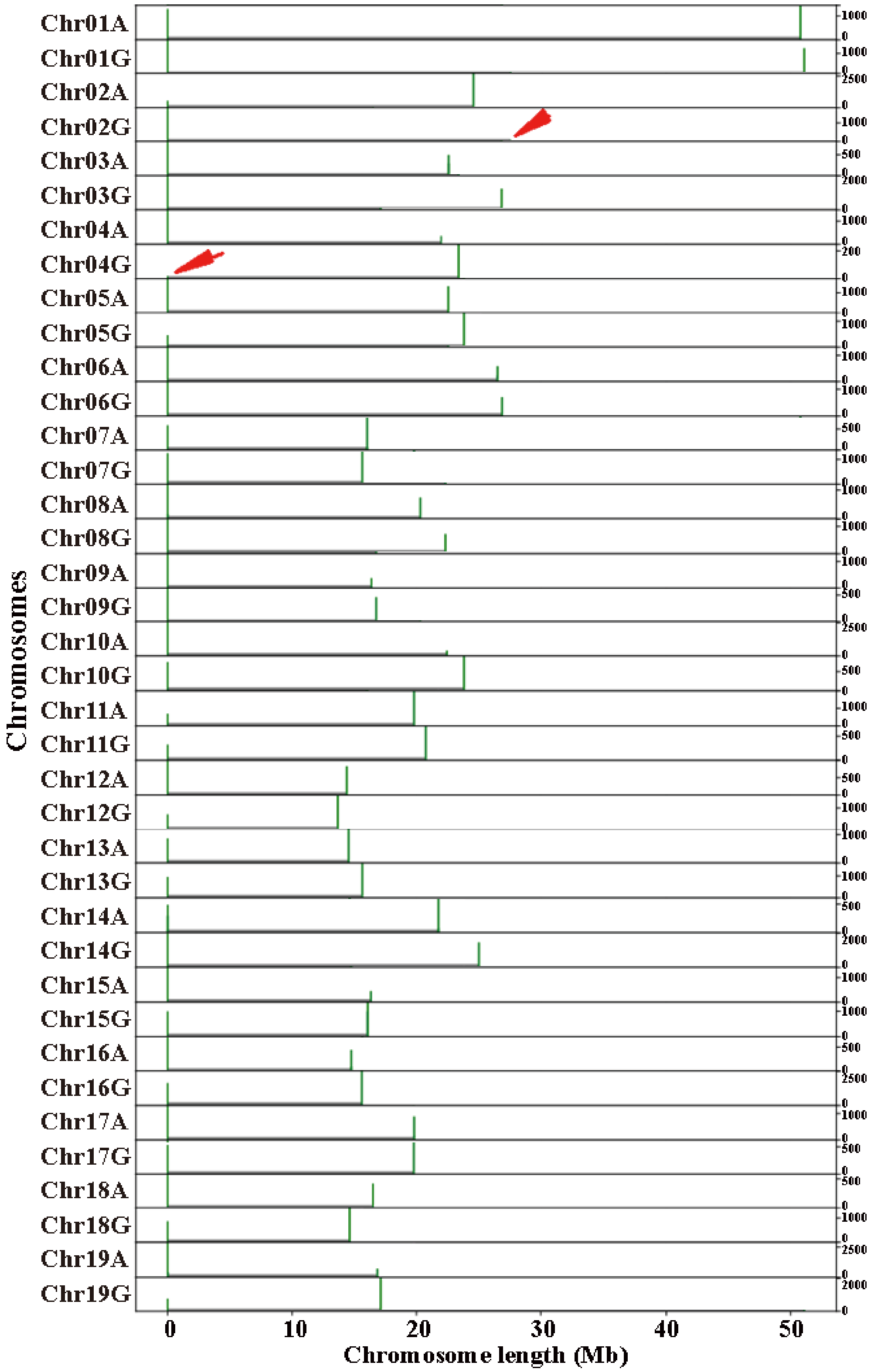

Supplemental Figure S4. Telomere sequences assembled in each chromosome. The green bars are indicating the potential telomeres with the high frequency telomere repeat sequences. Red arrows denote the potential telomeres with the presence of telomere repeats assembled but relatively low frequency of repeats. The scale on the right indicates the frequency of the telomere repeats. The length of the black line represents the length of the corresponding chromosome.

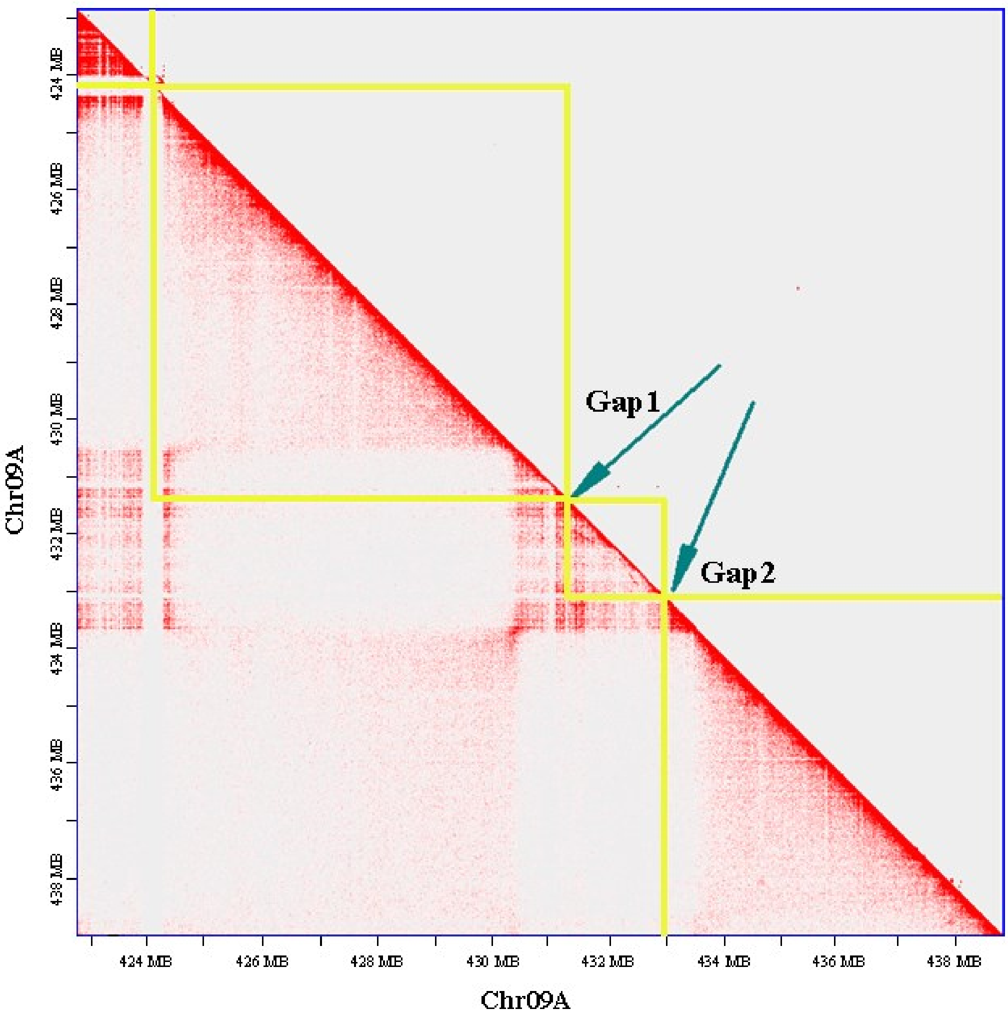

Supplemental Figure S5. Positions of the two gaps located on chromosome 9A (chr09A). The green arrows indicate the location of the gaps. The intensity of pixels represents the count of Hi-C links which indicate the likelihood of loci collocating in the nucleus. Darker red color indicates higher contact probability. The yellow lines are used to identify the position of the gaps.

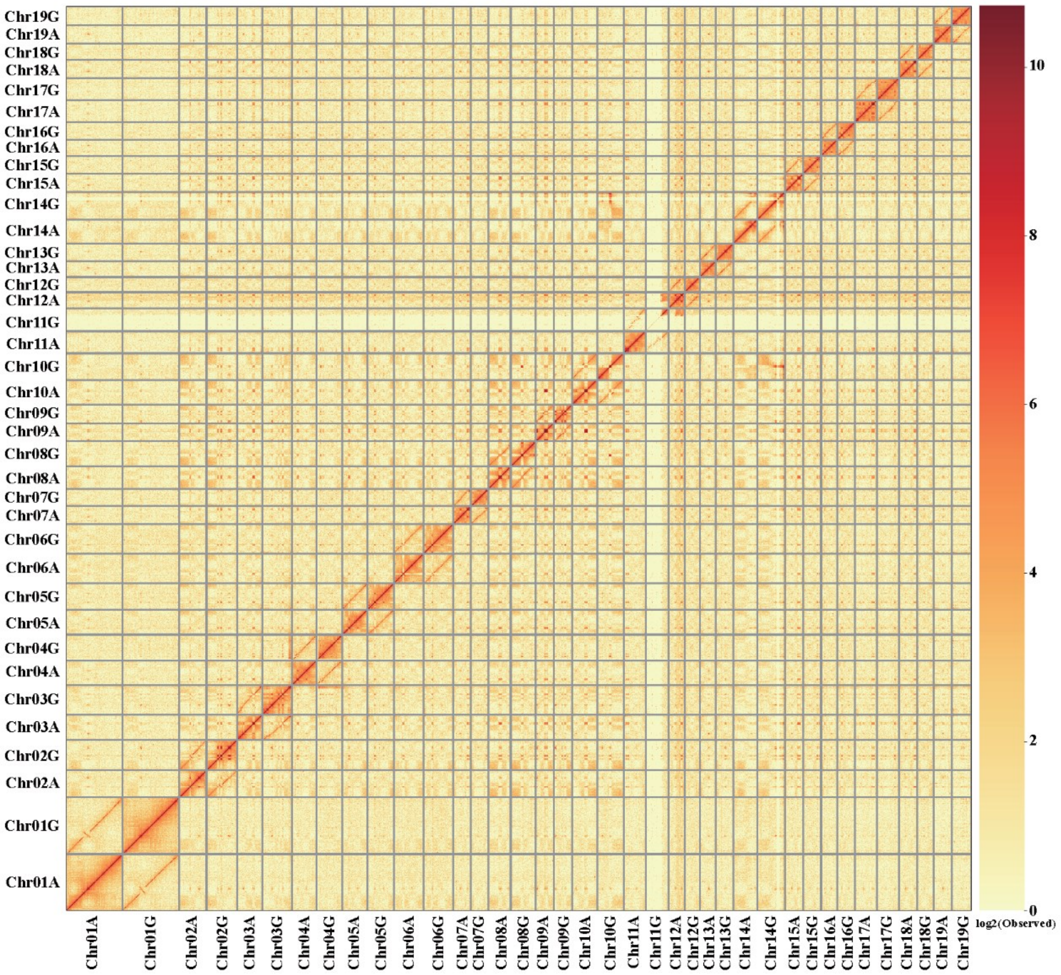

Supplemental Figure S6. Genome-wide analysis of chromatin interactions in the genome based on Hi-C data. The intensity of pixels represents the count of Hi-C links which indicate the likelihood of loci collocating in the nucleus. Darker red color indicates higher contact probability.

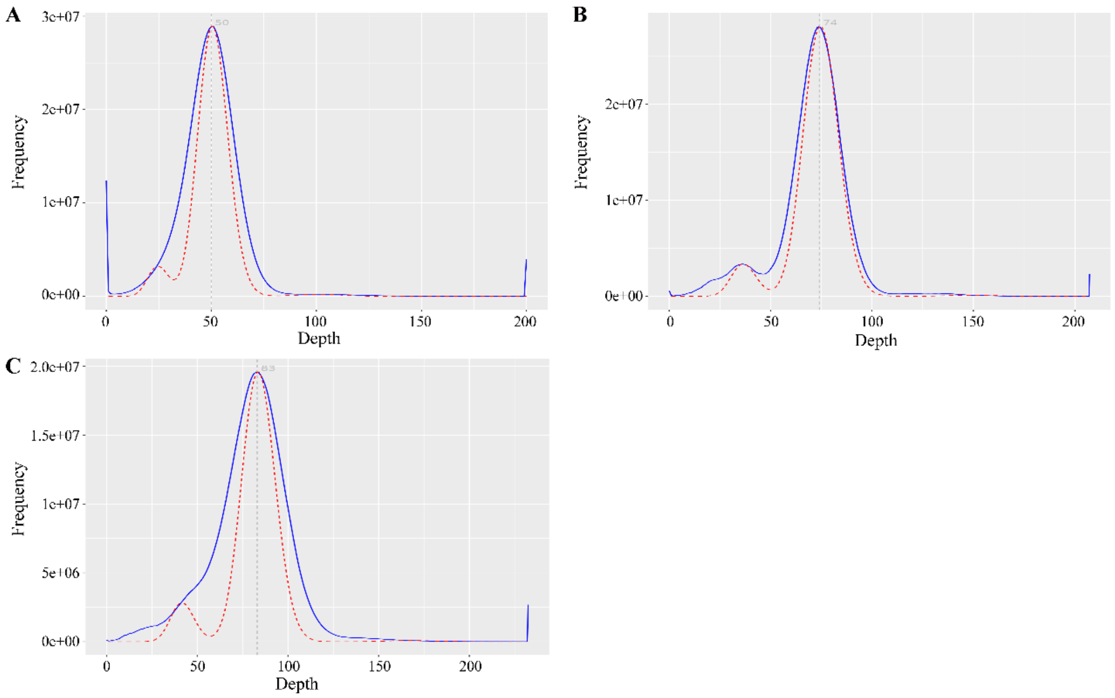

Supplemental Figure S7. *K*-mer frequency distribution estimated from Illumina (A), HiFi (B) and ONT sequences (C) after filtering and correction at *K*-mer size of 17. The x-axis shows the frequency or the number of times a given k-mer (k-mer depth). The y-axis shows the total number of k-mers with a given frequency (a given depth). *k*-mer refers to an artificial sequence division of *K* nucleotides. Genomic characteristics (genome size and repeat structure) can be estimated based on *k*-mer frequencies. Blue solid line for observed *k*-mer frequency distribution, red dash line for fitted model of *k*-mer frequency distribution.

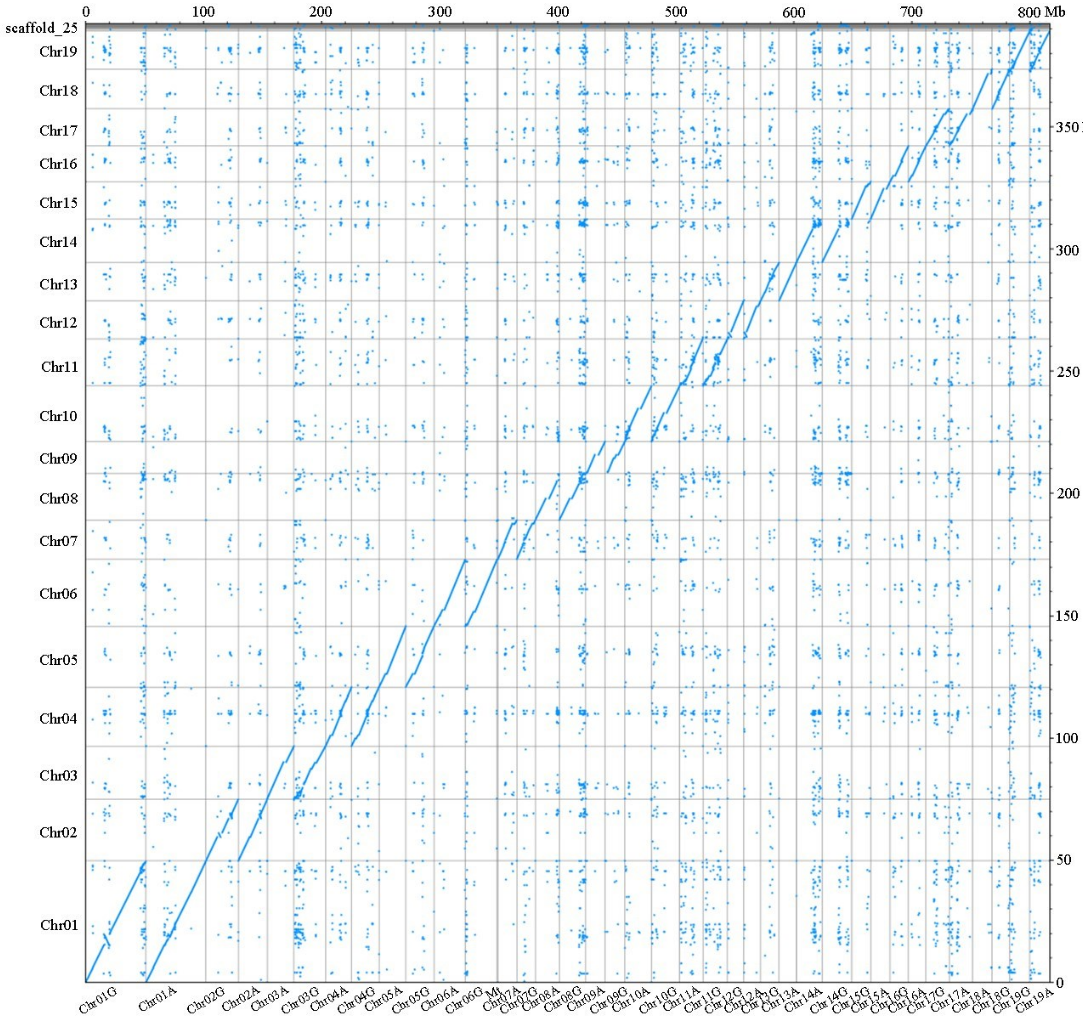

Supplemental Figure S8. Collinearity of two haplotype genomes of the poplar clone "84K" with that of *Populus trichocarpa*. The horizontal axis is the 84K assembly reported in this study. Chromosomes of two haplotype genomes of the poplar "84K" are in X-axis, those of *Populus trichocarpa* are in Y-axis.

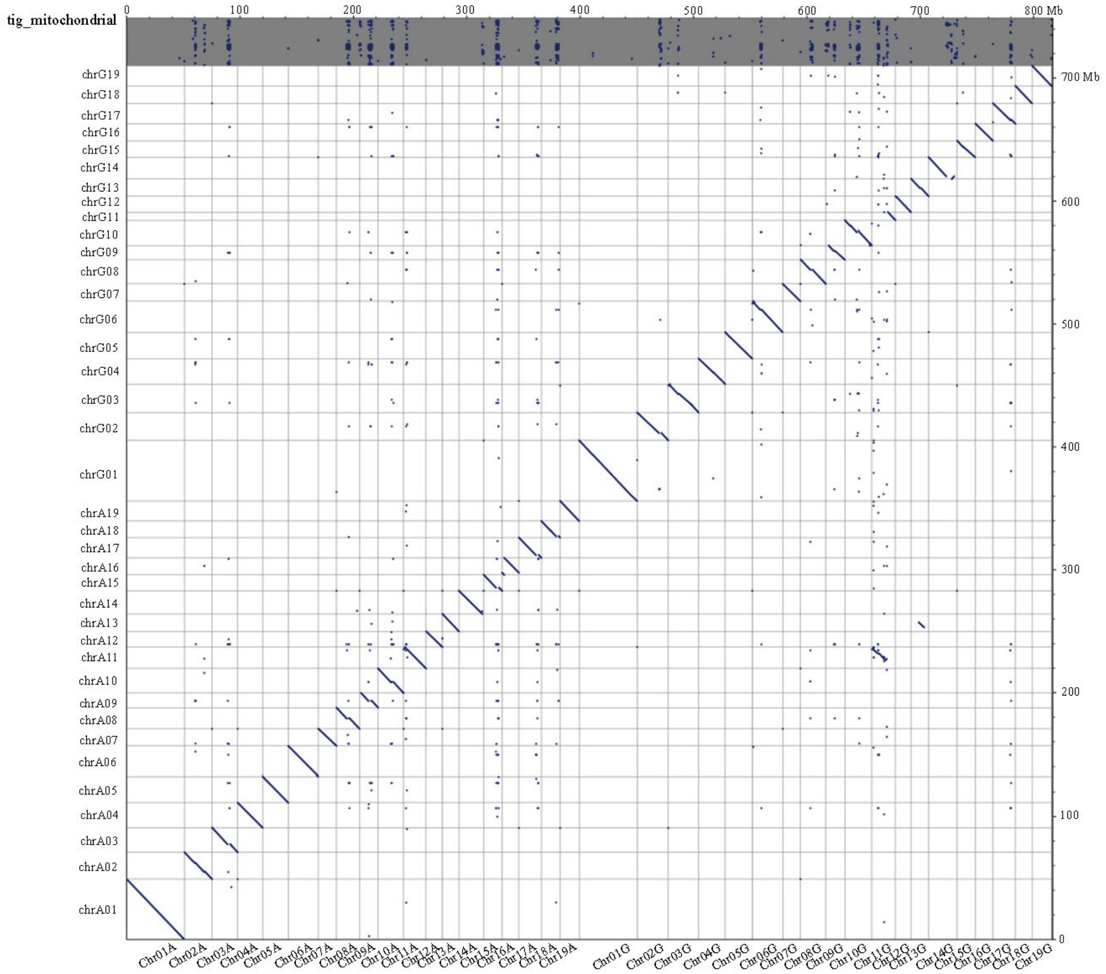

Supplemental Figure S9. Collinearity of two haplotype genomes of the current (this study) with published genomes of "84K" (Qiu et al. 2019). The horizontal axis is for the 84K assembly reported in this study. Chromosomes and contigs of two haplotype genomes of the poplar "84K" from this study are in X-axis, those from Qiu et al. 2019 are in Y-axis.

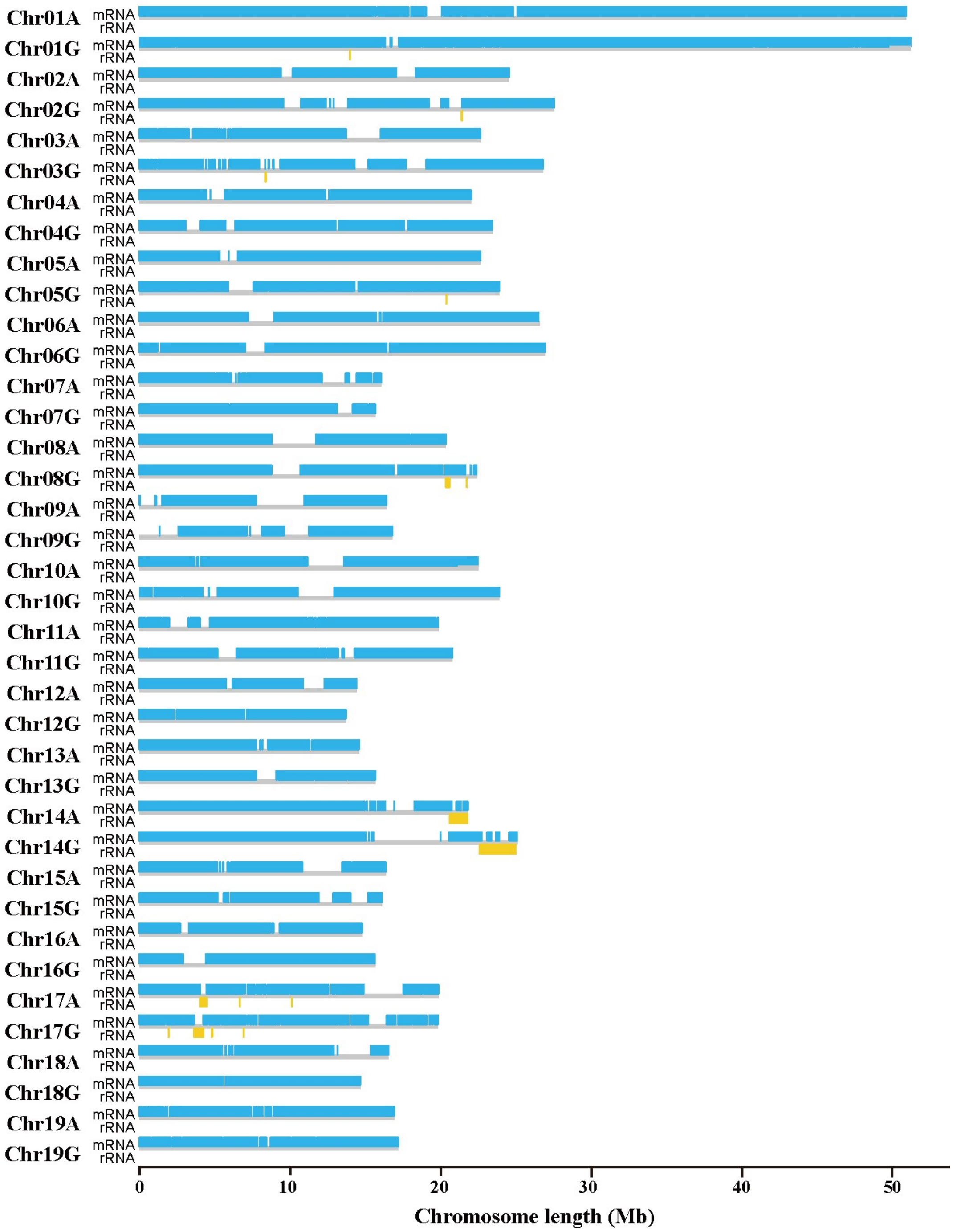

Supplemental Figure S10. Distribution of rDNA on chromosomes. The yellow areas indicate the distribution of the rDNA. The blue area indicates the distribution of mRNA.

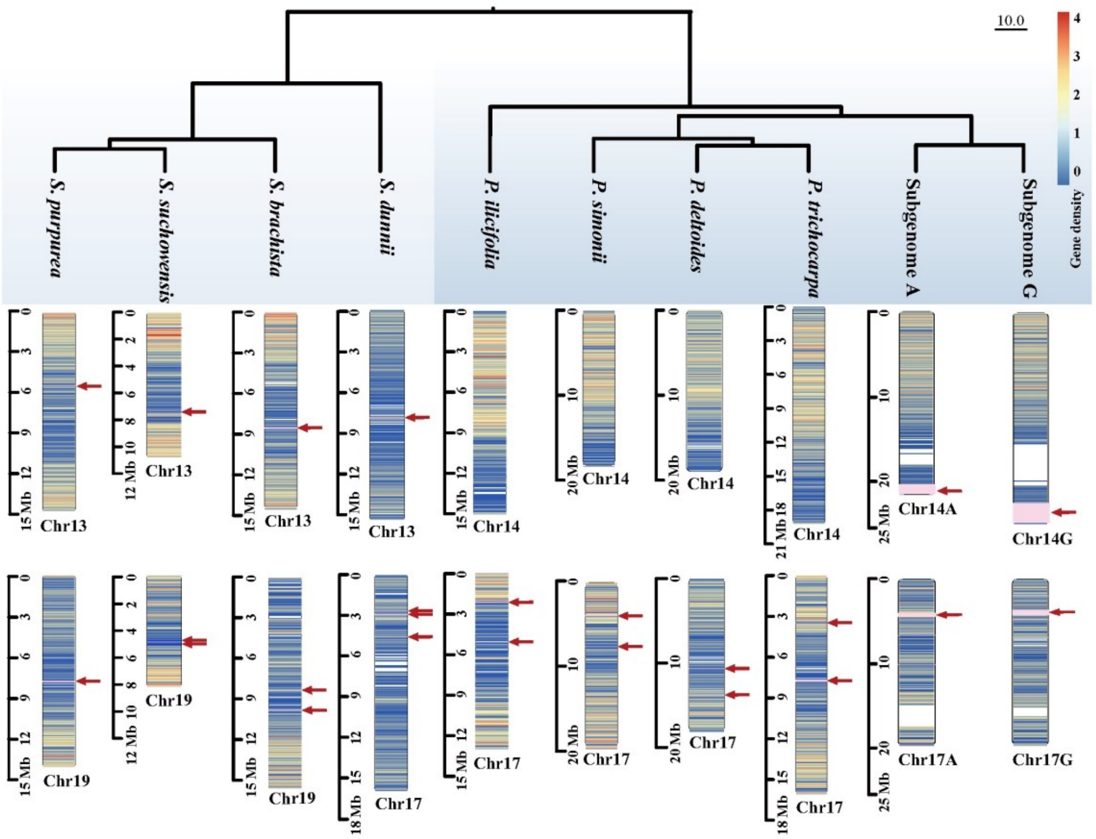

Supplemental Figure S11. Distribution of rDNA on chromosomes of Salicaceae species. The pink areas pointed with the red arrows indicate the locations of 5S rDNA cluster. The dark pink areas pointed with blue arrows indicate the locations of 45S rDNA clusters.

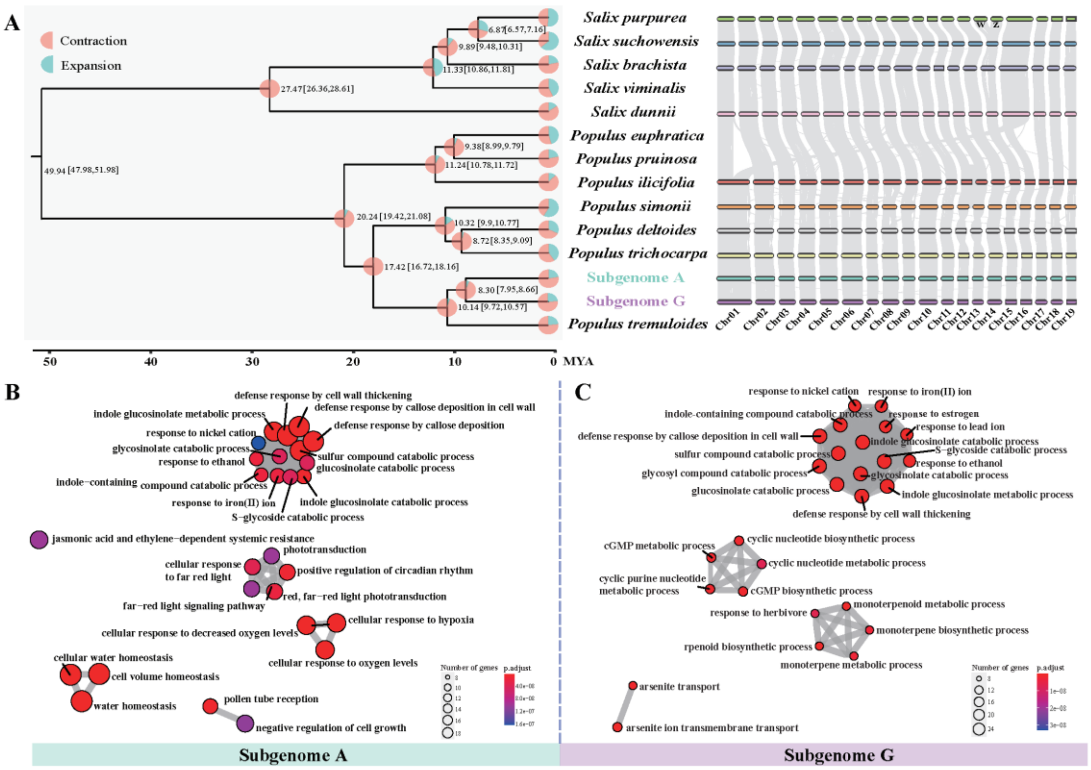

Supplemental Figure S12. Gene family evolution and collinearity analyses among Salicaceae species. A Genome evolutionary history and collinearity analyses of Salicaceae species. Chronogram showing the divergence times in Salicaceae, with node age and the 95% confidence intervals labeled. Pie charts show the proportions of gene families that underwent expansion or contraction. **B** Gene ontology (GO) enrichment analysis of expanded gene families in subgenome A. The size of the circles represents the number of genes in a GO term. The enriched GO terms with corrected *P* value < 0.005 are presented. The gray lines represent the shared genes between two GO terms. **C** Gene ontology (GO) enrichment analysis of expanded gene families in subgenome G. The enriched GO terms with corrected *P* value < 0.005 are presented. 'P-adjust' is the Benjamini-Hochberg false discovery rate (FDR) adjusted *P* value. The gray lines represent the shared genes between two GO terms.

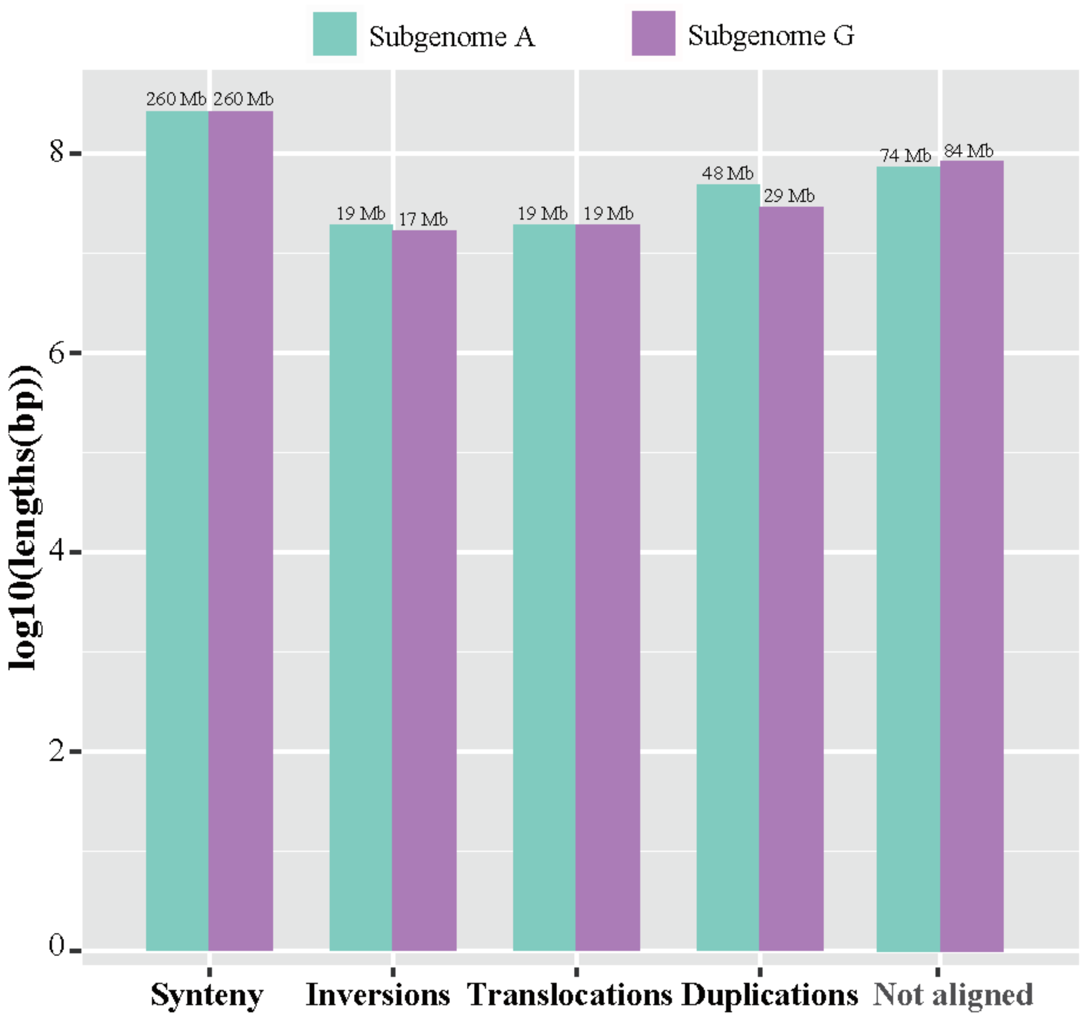

Supplemental Figure S13. Length of structural variation and local sequence differences between the subgenomes A and G (subgenome G for the assembly of *P. tremula* var. *glandulosa* and subgenome A for the assembly of *P. alba*).

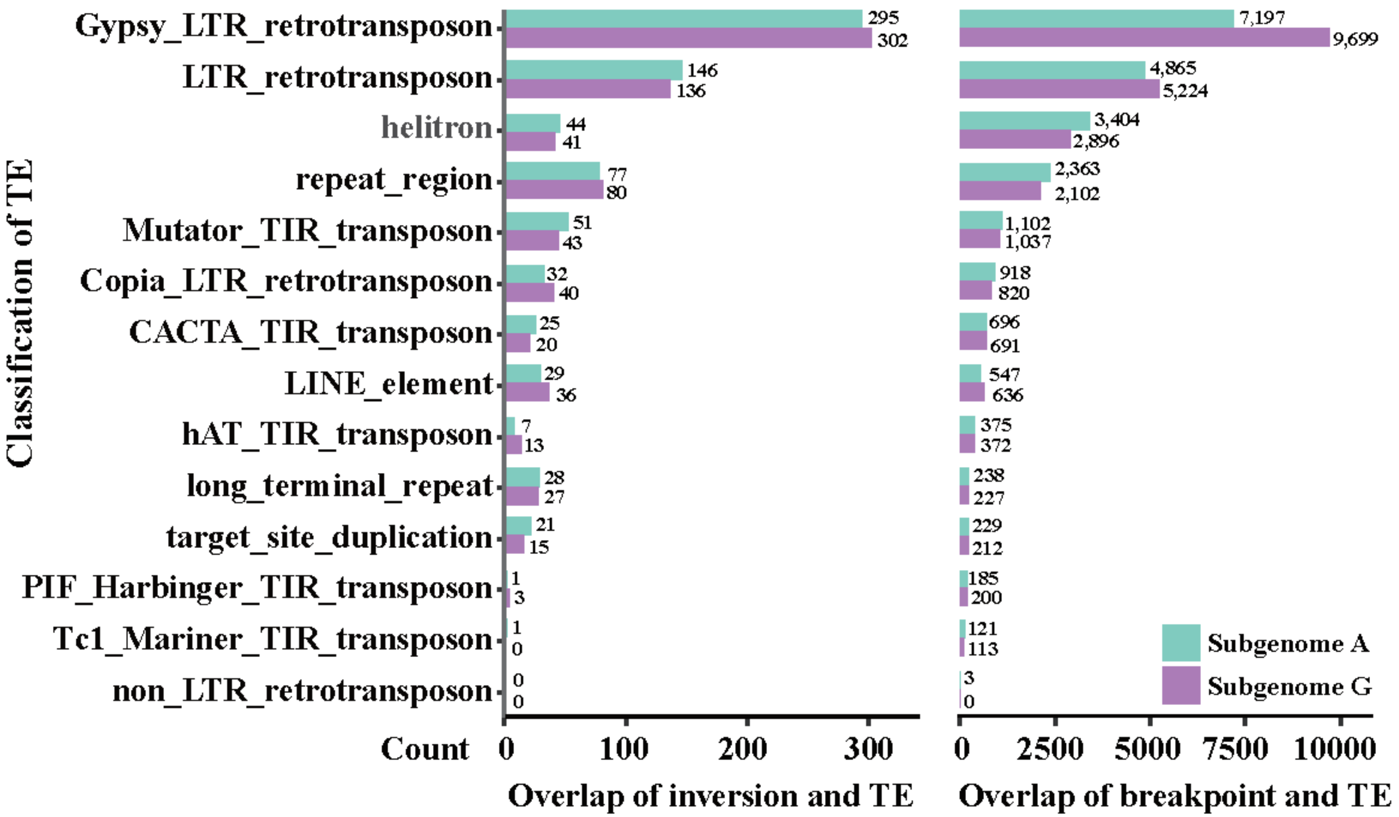

Supplemental Figure S14. Statistics on overlaps between the inversion regions and different TE types (left panel) and between breakpoint region of inversion and different TE types (right panel) in the two subgenomes (G for the assembly of *P. tremula* var. *glandulosa* and A for the assembly of *P. alba*). The inversion breakpoint regions were defined as the flanking region of 150 bp in the upstream and downstream of an inversion breakpoint. LTR: Long Terminal Repeat; TIR: Terminal Inverted Repeats; LINE: Long Interspersed Repetitive Element; hAT: hobo, Activator, Tam3; PIF: P Instability Factor.

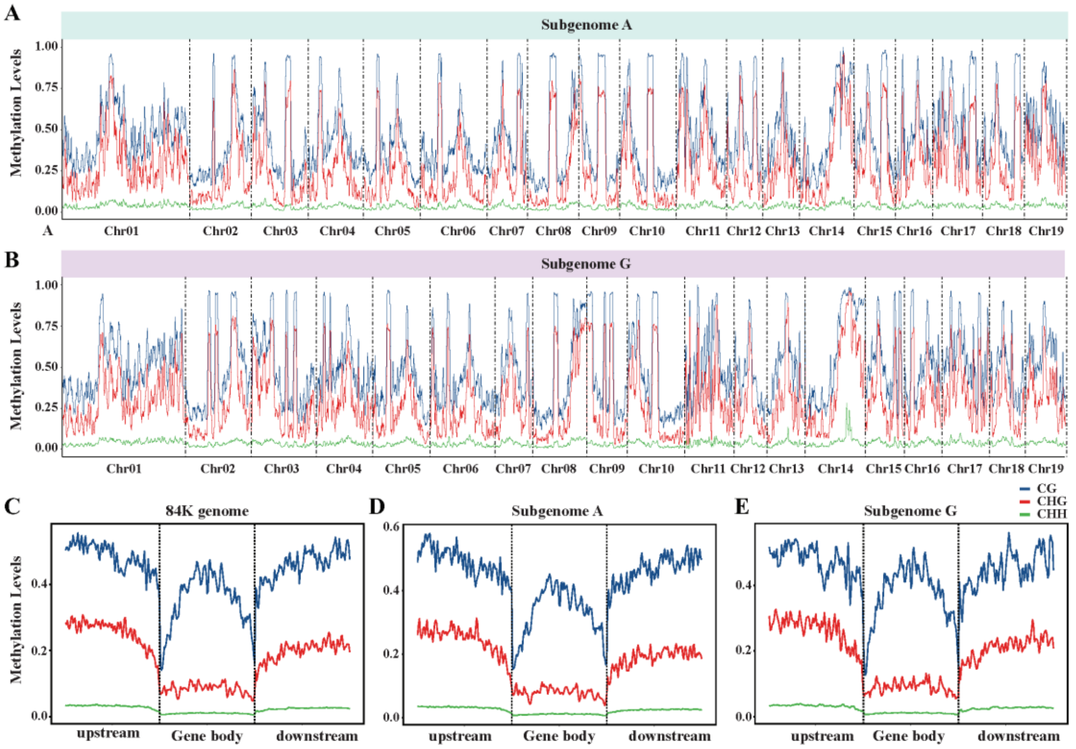

Supplemental Figure S15. DNA methylation patterns. A Distribution of CG, CHG, and CHH DNA methylation levels along the chromosomes of *P. alba* genome (the subgenome A). DNA methylation level indicates the average methylation level, i.e., the number of methylated reads coverage/all reads coverage (the sum of methylated reads coverage and unmethylated reads coverage) within the region. **B** Distribution of CG, CHG, and CHH DNA methylation levels along the chromosomes of *P. tremula* var. *glandulosa* genome (the subgenome G). **C-E** Distribution of CG, CHG, and CHH DNA methylation levels among gene features, including gene body and upstream and downstream regions. "Subgenome A" represents one of the parents *Populus alba*. "Subgenome G" represents the other parent *Populus tremula* var. *glandulosa*. The "84K genome" represents the average of two subgenomes.

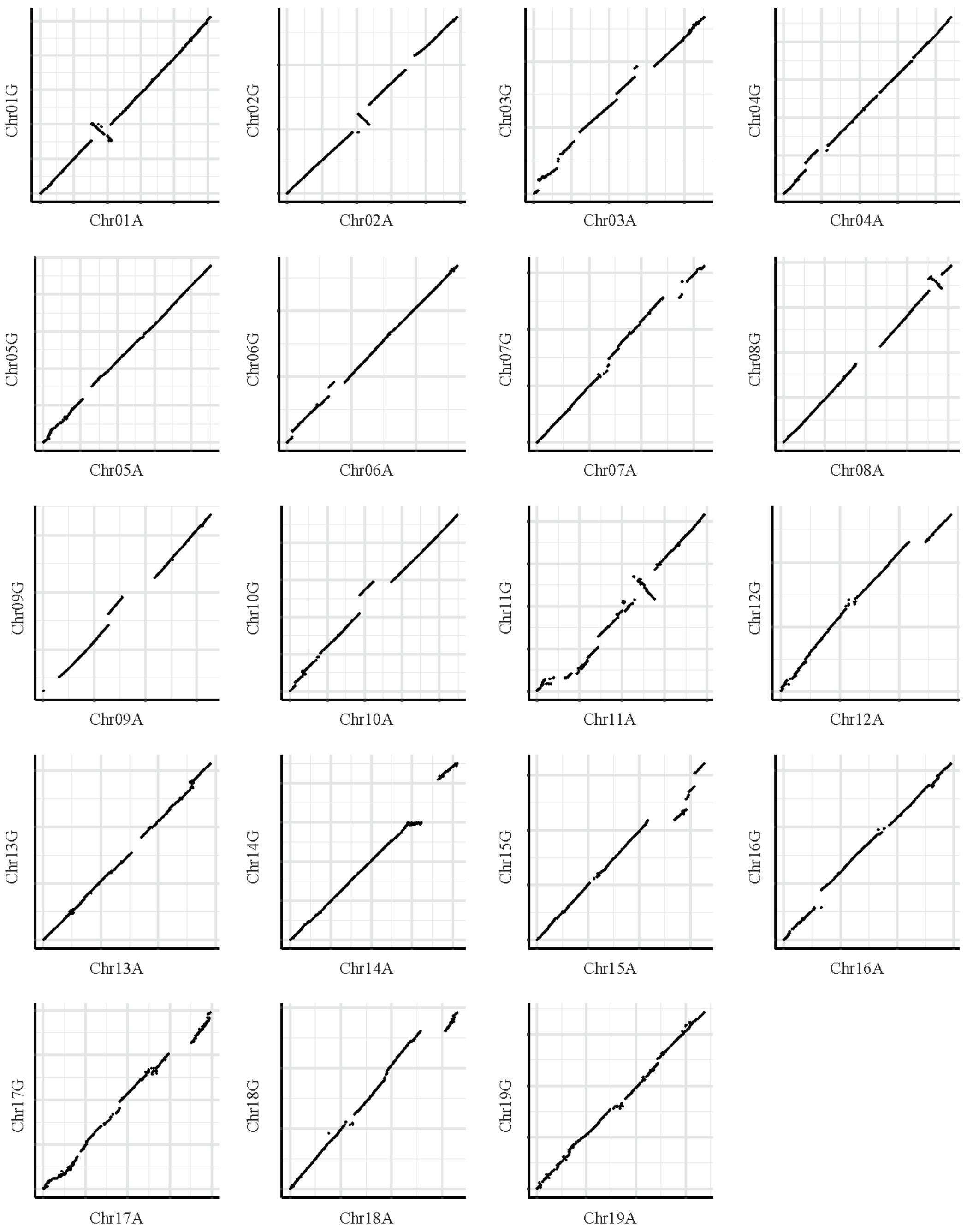

Supplemental Figure S16. Collinearity of a pair of alleles on two parental genomes. Chromosome names ending with "A" denotes they are of the subgenome A, that is for the nearly assembly of *P. alba*; and those ending with "G" are of subgenome G, that is the assembly of *P. tremula* var. *glandulosa*.

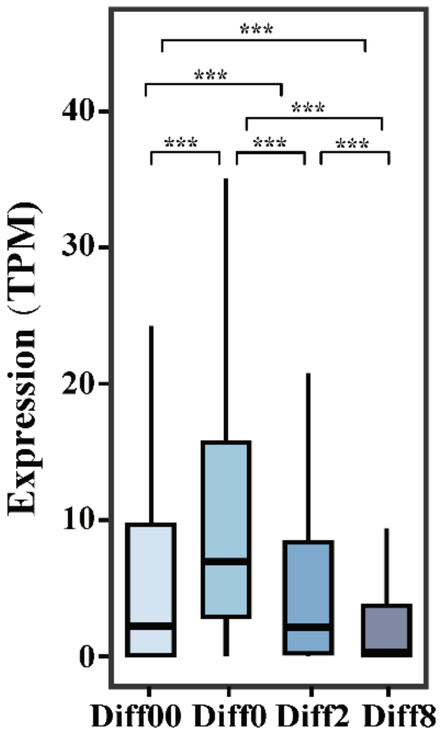

Supplemental Figure S17. Absolute TPM expression abundance for Diff00, Diff0, Diff2, Diff8. Mann-Whitney-Wilcoxon test. *p < 0.05; **p < 0.01; ***p < 0.001. Diff00: non-significant difference between a pair of alleles with p-adjust > 0.05; Diff0: significant difference between a pair of alleles with p-adjust ≤ 0.05 and fold change (FC) ≤ |2|; Diff2: significant difference between a pair of alleles with p-adjust ≤ 0.05 and |2| < FC < |8|; Diff8: significant difference between a pair of alleles with p-adjust ≤ 0.05 and FC ≥ |8|. TPM: Transcripts Per Million. Boxplots were made using the expression levels of alleles contained in the four groups (Diff00, Diff0, Diff2 and Diff8). In boxplots, the center line in the box indicates the median value, and the box height indicates the 25th to 75th percentiles of the total data. Whiskers indicate the 1.5× interquartile range.

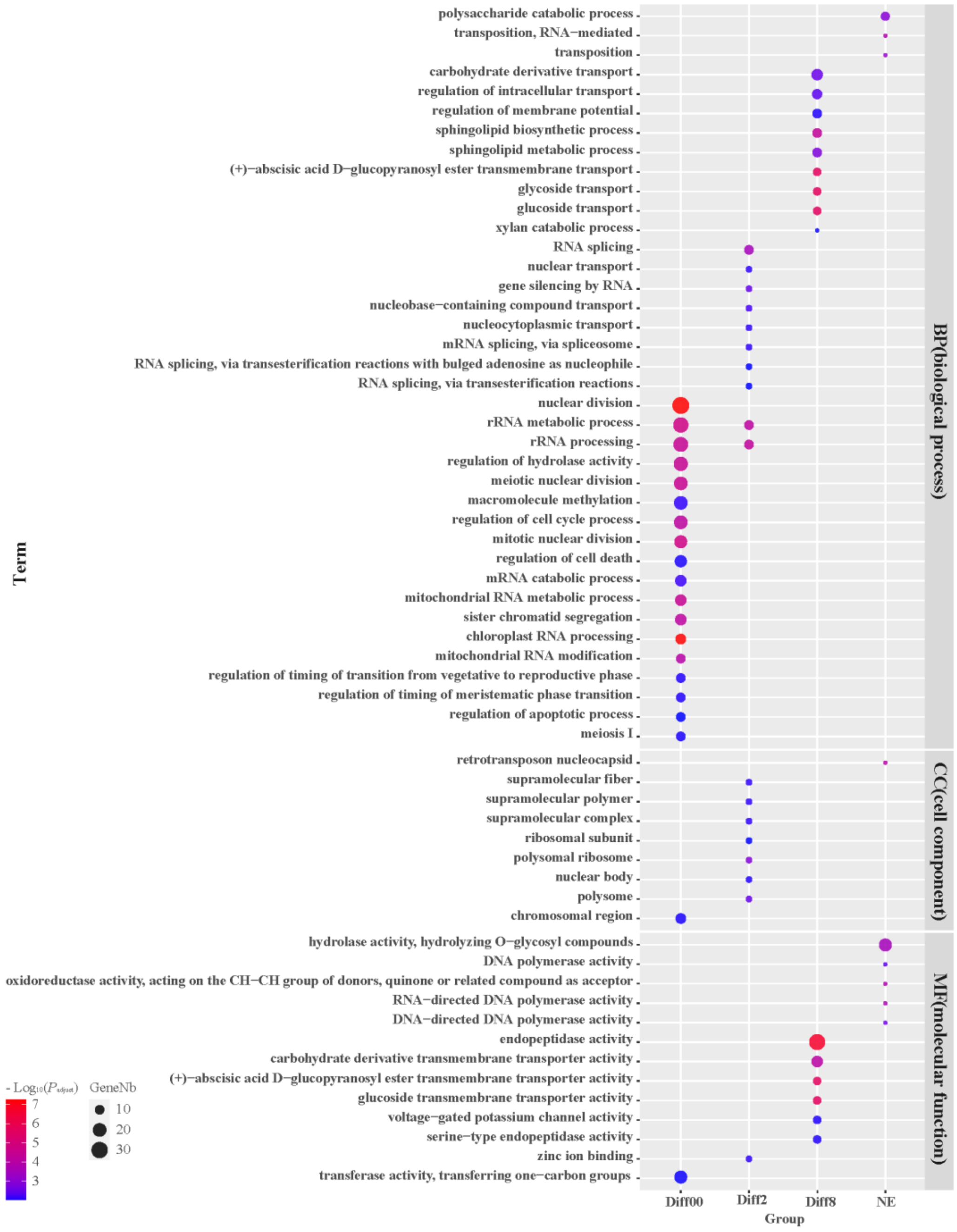

Supplemental Figure S18. Gene ontology (GO) enrichment analysis of five categories of allelic expression bias. The enriched GO terms with corrected *P* value <0.005 are presented. The color of circles represents the statistical significance of enriched GO terms. The size of the circles represents the number of genes in a GO term. For all annotated genes their GO terms are provided as background information. 'P-adjust' is the Benjamini-Hochberg false discovery rate (FDR) adjusted P value. NE**:** non-expression (both alleles are not expressed)**;** Diff00: non-significant difference between a pair of alleles with *p*-adjust > 0.05; Diff0: significant difference between a pair of alleles with *p*-adjust ≤ 0.05 and fold change (FC) ≤ |2|; Diff2: significant difference between a pair of alleles with *p*-adjust ≤ 0.05 and |2| < FC < |8|; Diff8: significant difference between a pair of alleles with *p*-adjust ≤ 0.05 and FC ≥ |8|. The p-value is obtained from the Fisher exact test. The q-value is obtained by using the BH (Benjamini-Hochberg) method to control the FDR.

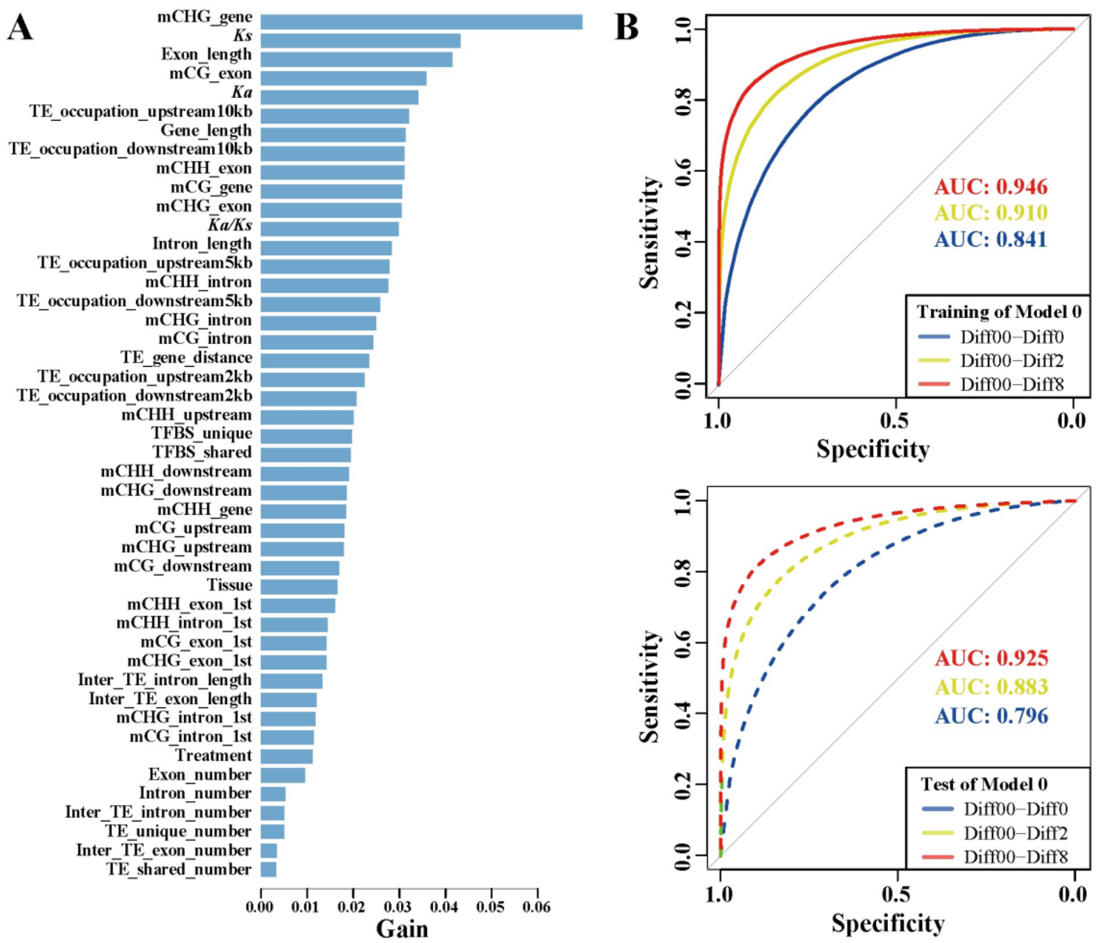

Supplemental Figure S19. Importance ranking and ROC (receiver operating characteristic) curves of Model 0 (with 46 predictors/features). A Ranking of the importance of 46 features predicting allele-specific expression (ASE) in the Model 0. Model 0 is constructed with all 46 features as predictors to predicate one response with four ASE groups (Diff00, Diff0, Diff2 and Diff8). Diff00: non-significant difference between a pair of alleles with *p*-adjust > 0.05; Diff0: significant difference between a pair of alleles with *p*-adjust ≤ 0.05 and fold change (FC) ≤ |2|; Diff2: significant difference between a pair of alleles with *p*-adjust ≤ 0.05 and |2| < FC < |8|; Diff8: significant difference between a pair of alleles with *p*-adjust ≤ 0.05 and FC ≥ |8|. See Supplementary Note 1 and Figure 4 for details on each feature. **B** ROC (Receiver Operating Characteristic) curves and AUC (Area Under the Curve) values of the XGBoost model (Model 0). TE: transposable elements. TFBS: transcription factor binding site. *Ks*: number of substitutions per synonymous site. *Ka*: number of substitutions per nonsynonymous site. *Ka*/*Ks* values were estimated for alleles generated based on the Yang-Nielsen model (YN).

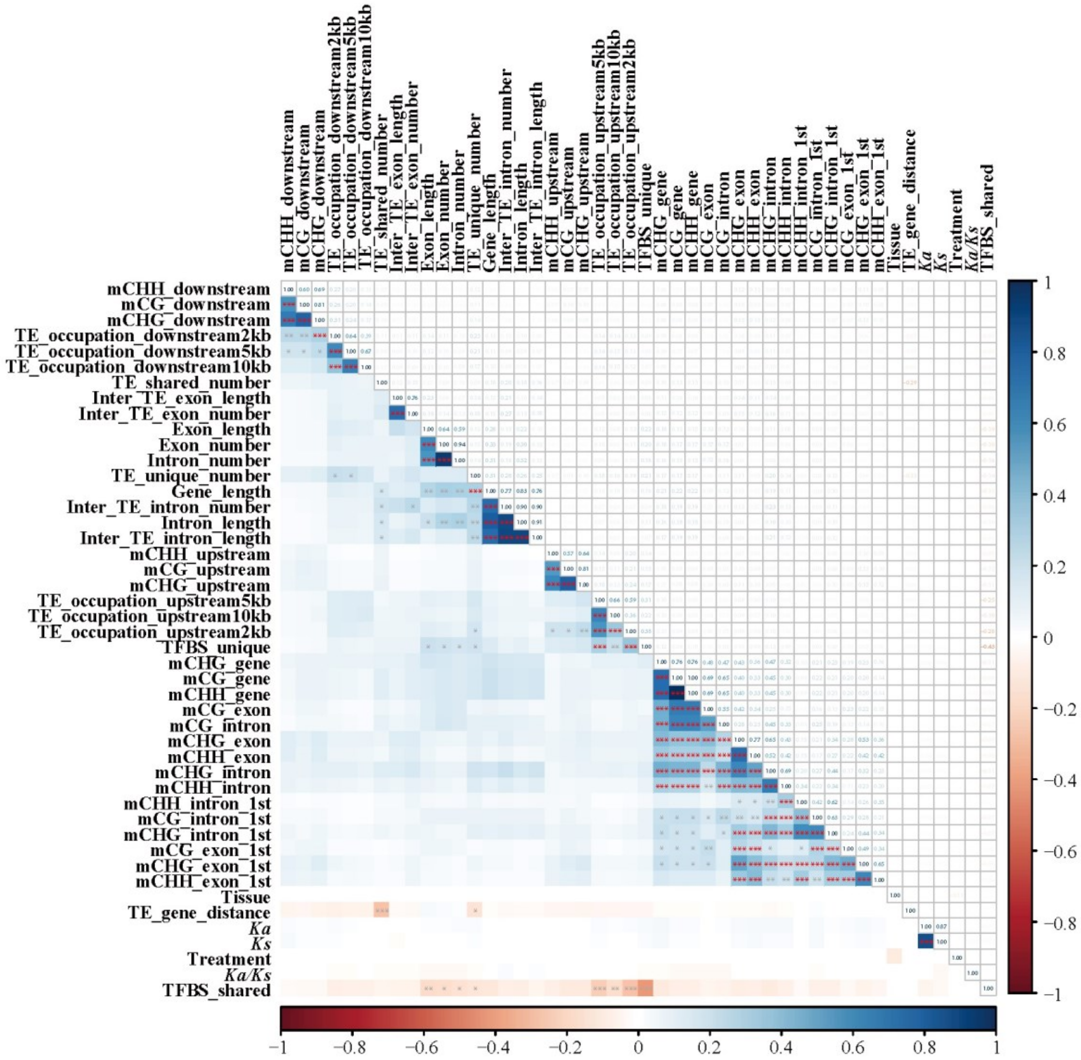

Supplemental Figure S20. Pair-wise correlation among 46 predictors (features) used in modeling (Model 0). Correlation analysis (Pearson correlation, two-sided test) using the "cor" function in R. *p < 0.05, **p < 0.01, ***p < 0.001. The bars represent the values of the correlation coefficients between different variables. The blue color variation indicates positive correlation, while the red color variation indicates negative correlation. TE: transposable elements. TFBS: transcription factor binding site. *Ks*: number of substitutions per synonymous site. *Ka*: number of substitutions per nonsynonymous site. *Ka*/*Ks* values were estimated for alleles generated based on the Yang-Nielsen model (YN).

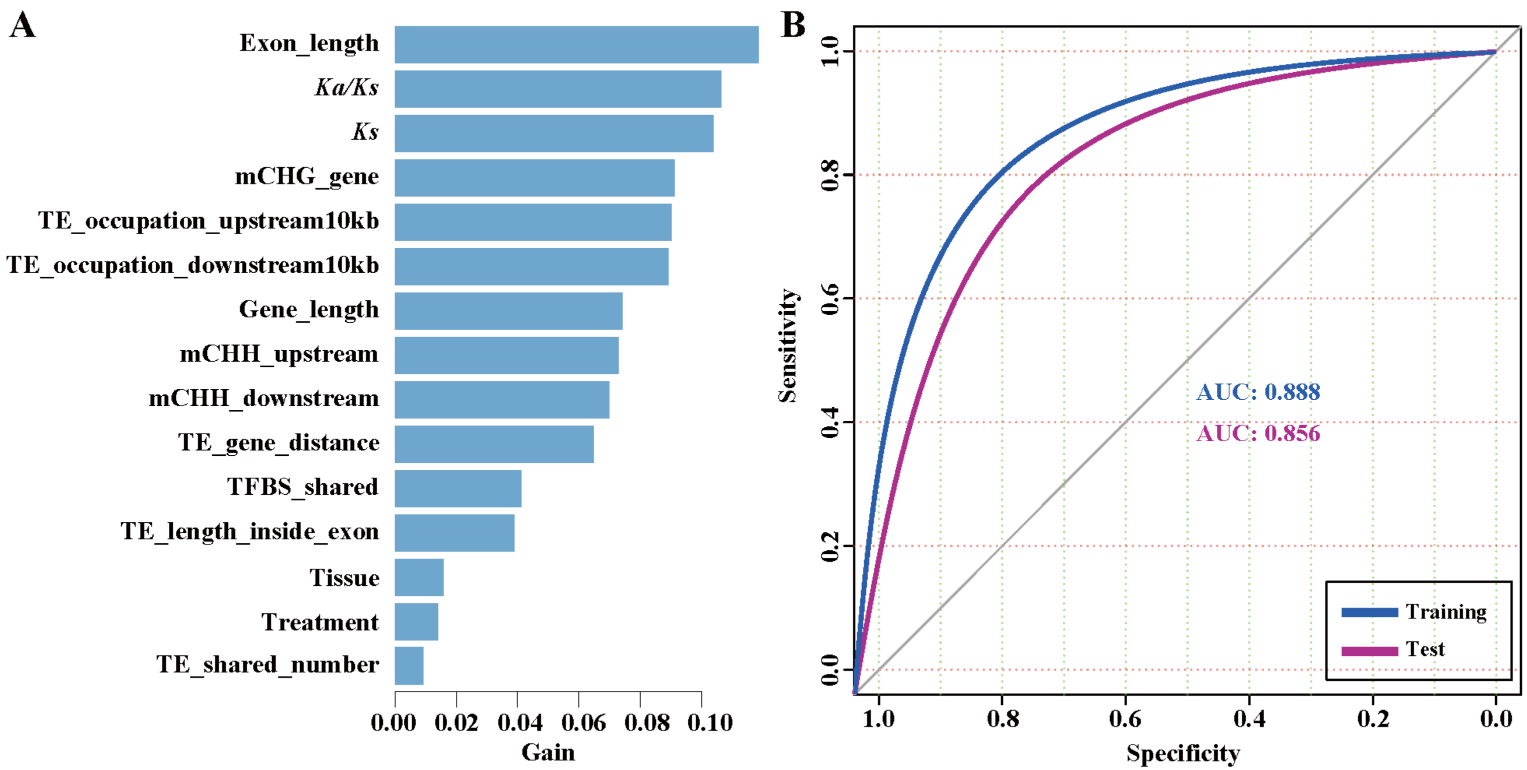

Supplemental Figure S21. Ranking of the 15 features in the XGBoost model (Model 2) and the model assessment. A Ranking of the 15 features in the XGBoost model (Model 2). Model 2: A XGBoost classification model with 15 predictors and one response with two groups (group 1 with: Diff0, Diff2 and Diff8; group 2 with: Diff00). Diff00: non-significant difference between a pair of alleles with p-adjust > 0.05; Diff0: significant difference between a pair of alleles with p-adjust $\leq$ 0.05 and fold change (FC) $\leq$ |2|; Diff2: significant difference between a pair of alleles with p-adjust $\leq$ 0.05 and |2| < FC < |8|; Diff8: significant difference between a pair of alleles with p-adjust $\leq$ 0.05 and FC $\geq$ |8|. **B** ROC (Receiver Operating Characteristic) curves and AUC (Area Under the Curve) values of the XGBoost model (Model 2). TE: transposable elements. *Ks*: number of substitutions per synonymous site. *Ka*: number of substitutions per nonsynonymous site. *Ka*/*Ks* values were estimated for alleles generated based on the Yang-Nielsen model (YN). TFBS: transcription factor binding site.

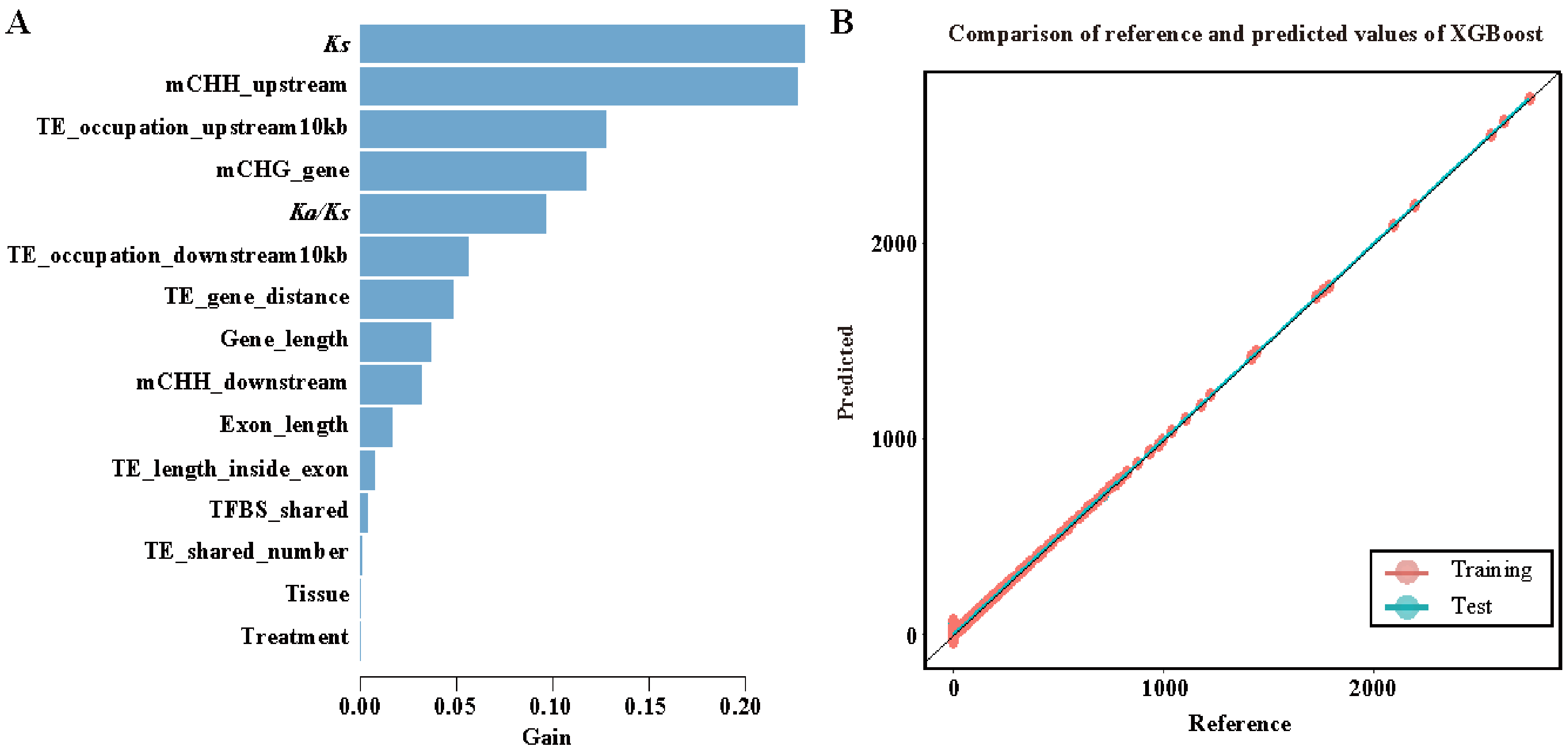

Supplemental Figure S22. Ranking of the 15 features in the XGBoost model (Model 3) and the model assessment. A Ranking of the 15 features in the XGBoost model (Model 3). **B** Comparison of reference and predicted values of XGBoost. Model 3: A XGBoost regression model with 15 predictors and the difference in expression as response (in transcripts per million, TPM) of ASE. Diff00: non-significant difference between a pair of alleles with *p*-adjust > 0.05; Diff0: significant difference between a pair of alleles with *p*-adjust ≤ 0.05 and fold change (FC) ≤ |2|; Diff2: significant difference between a pair of alleles with *p*-adjust ≤ 0.05 and |2| < FC < |8|; Diff8: significant difference between a pair of alleles with *p*-adjust ≤ 0.05 and FC ≥ |8|. TE: transposable elements. TFBS: transcription factor binding site. *Ks*: number of substitutions per synonymous site. *Ka*: number of substitutions per nonsynonymous site. *Ka*/*Ks* values were estimated for alleles generated based on the Yang-Nielsen model (YN).

Supplemental Tables

Supplemental Table S1: Statistics of whole genome sequencing data.

Technologies	Features	Statistics
**HiFi sequencing**	Total bases (bp)	70,664,880,148
	GC content	35.70%
	A	22,716,563,991 (32.15%)
	T	22,723,016,538 (32.16%)
	G	12,622,049,161 (17.86%)
	C	12,603,250,458 (17.84%)
	Total reads	6,642,258
	Max. (bp)	39,591
	Min. (bp)	45
	Mean (bp)	10,638
	Median (bp)	10,114
	N10 (bp)	14,871
	N50 (bp)	10,457
	N90 (bp)	8,644
	L10	443,929
	L50	2,800,531
	L90	5,777,520
**ONT sequencing**	Total bases	80,815,876,125
	GC content	34.91%
	A	26,219,402,451 (32.44%)
	T	26,380,015,791 (32.64%)
	G	13,721,347,620 (16.98%)
	C	14,495,110,263 (17.94%)
	Total reads	6,331,056
	Max. (bp)	626,925
	Min. (bp)	1
	Mean (bp)	12,764
	Median (bp)	5,736
	N10 (bp)	69,631
	N50 (bp)	28,188
	N90 (bp)	5,791
	L10	90,232
	L50	876,401
	L90	3,149,514
**Hi-C**	Reads (Mb)	679.801
	Bases (Gb)	101.97
	Q20 (Gb)	98.829 (96.9%)
	Q30 (Gb)	93.763 (92.0%)
	Average Length (bp)	150

Supplemental Table S2: Summary of the Illumina reads for the genome assembly of "84K".

Species	Sample	Raw Reads (Mb)	Raw Bases (Gb)	Raw Q20 (Gb)	Raw Q30 (Gb)
**84K**	SRR9831374	61.968	9.295	8.949(96.27%)	8.544(91.92%)
	SRR9831375	12.973	1.946	1.880(96.59%)	1.805(92.77%)
	SRR9831376	63.188	9.478	9.130(96.32%)	8.741(92.22%)
	SRR9831377	185.719	27.858	26.415(94.82%)	25.005(89.76%)
P. alba	SRR11587873	204.868	30.73	29.242(95.16%)	27.118(88.24%)
P. tremula* var. *glandulosa	SRR11853653	307.979	46.505	44.555(95.81%)	41.819(89.92%)

Species	Clean Reads (Mb)	Clean Bases (Gb)	Clean Q20 (Gb)	Clean Q30 (Gb)	Average Length (bp)
**84K**	60.192(97.13%)	8.979(96.60%)	8.751(97.46%)	8.544(93.61%)	149.2
	12.643(97.45%)	1.886(96.93%)	1.843(97.69%)	1.805(94.32%)	149.2
	61.375(97.13%)	9.153(96.57%)	8.926(97.53%)	8.741(93.93%)	149.1
	175.848(94.68%)	26.122(93.77%)	25.284(96.79%)	25.005(92.53%)	148.5
P. alba	202.105(98.65%)	30.216(98.33%)	28.878(95.57%)	27.118(88.76%)	149.5
P. tremula* var. *glandulosa	303.110(98.42%)	45.403(97.63%)	43.845(96.57%)	41.819(90.86%)	149.8

Supplemental Table S3: Statistics of the different versions of genome assembly.

Versions of assembly	Strategy	Assemblied genome size (Mb)	Sequence number	N50 of contigs (Mb)	N50 of scaffolds (Mb)	Gene completeness (%)
**v0.1**	hifiasm + purge_dups	928.25	3,527	12.7	-	96.10%
**v1.0**	v0.1 + 3d-dna + gapclose + Optimization + polish + Redundans	816.95	50	22.36	22.36	96.50%

Supplemental Table S4: Statistics of the genome quality for the final assembly.

	Number			Length (bp)			Percent (%)		
	84K	Subgenome A	Subgenome G	84K	Subgenome A	Subgenome G	84K	Subgenome A	Subgenome G
**chromosome-scale scaffold**	38	19	19	815,587,126	-	-	99.83%	-	-
**mitochondrial**	1	-	-	838,650	-	-	0.10%	-	-
**chloroplast**	1	-	-	156,505	-	-	0.02%	-	-
**contig-scale scaffold**	10	-	-	366,730	-	-	0.04%	-	-
**genome size**	-	-	-	816,949,011	400,191,317	416,757,694	-	-	-
**genome size without N**	-	-	-	816,948,811	400,191,117	416,757,694	-	-	-
**GC content**	-	-	-	-	-	-	34.87	34.69	35.05
**A**	265,970,022	130,856,957	135,113,065	-	-	-	32.56	32.70	32.42
**T**	266,074,001	130,518,025	135,555,976	-	-	-	32.57	32.61	32.53
**C**	142,307,931	69,324,863	72,983,068	-	-	-	17.42	17.32	17.51
**G**	142,596,857	69,491,272	73,105,585	-	-	-	17.45	17.36	17.54
**N**	200	200	0	-	-	-	0	0	0
**contig**	52	25	27	-	-	-	-	-	-
**contig Max**	-	-	-	51,099,886	50,794,782	51,099,886	-	-	-
**contig Mean**	-	-	-	15,710,554	16,007,644	15,435,470	-	-	-
**contig N10**	-	-	-	50,794,782	50,794,782	51,099,886	-	-	-
**contig N50**	-	-	-	22,356,518	21,760,028	23,408,591	-	-	-
**contig N90**	-	-	-	14,770,136	14,562,659	15,622,777	-	-	-
**contig Min**	-	-	-	18,503	39,269	18,503	-	-	-
**contig Median**	-	-	-	16,337,227	16,512,846	16,088,989	-	-	-
**contig L10**	2	1	1	-	-	-	-	-	-
**contig L50**	15	8	8	-	-	-	-	-	-

Type	Number			Length (bp)			Percent (%)		
	84K	Subgenome A	Subgenome G	84K	Subgenome A	Subgenome G	84K	Subgenome A	Subgenome G
**contig L90**	33	17	17	-	-	-	-	-	-
**scaffold**	50	23	27	-	-	-	-	-	-
**scaffold Max**	-	-	-	51,099,886	50,794,782	51,099,886	-	-	-
**scaffold Mean**	-	-	-	16,338,980	17,399,622	15,435,470	-	-	-
**scaffold N10**	-	-	-	50,794,782	50,794,782	51,099,886	-	-	-
**scaffold N50**	-	-	-	22,356,518	21,760,028	23,408,591	-	-	-
**scaffold N90**	-	-	-	15,622,777	14,770,136	15,622,777	-	-	-
**scaffold Min**	-	-	-	18,503	39,269	18,503	-	-	-
**scaffold Median**	-	-	-	16,512,846	16,882,408	16,088,989	-	-	-
**scaffold L10**	2	1	1	-	-	-	-	-	-
**scaffold L50**	15	8	8	-	-	-	-	-	-
**scaffold L90**	33	17	17	-	-	-	-	-	-
**gap**	2	2	0	-	-	-	-	-	-
**gap Max**	-	-	-	100	-	-	-	-	-
**gap Mean**	-	-	-	100	-	-	-	-	-
**gap Min**	-	-	-	100	-	-	-	-	-
**gap Median**	-	-	-	100	-	-	-	-	-

Note:

-: data not available.

Supplemental Table S5: Mapping rates of Illumina reads, HiFi reads, and ONT reads to the present genome assembly of “84K”.

Data set	Reads mapped	Properly paired	Bases mapped	>=1×	>=5×	>=10×	>=20×
**Illumina**	96.60%	94.80%	96.60%	98.50%	98.30%	98.10%	96.90%
**HiFi**	97.80%	-	97.70%	99.93%	99.90%	99.80%	98.80%
**ONT**	99.60%	-	99.90%	99.99%	99.94%	99.80%	99.00%

Note:

-: data not available.

Supplemental Table S6: Summary of BUSCO evaluation for genome assembly and gene prediction.

	Genome assembly					
	BUSCO groups			Percentage		
	84K	Subgenome A	Subgenome G	84K	Subgenome A	Subgenome G
Complete BUSCOs	1,389	1,392	1,392	96.50%	96.67%	96.67%
Complete and single-copy BUSCOs	76	1,197	1,200	5.28%	83.13%	83.33%
Complete and duplicated BUSCOs	1,313	195	192	91.18%	13.54%	13.33%
Fragmented BUSCOs	5	10	10	0.35%	0.69%	0.69%
Missing BUSCOs	46	38	38	3.19%	2.64%	2.64%
Total BUSCOs groups searched	1,440	1,440	1,440	100%	100%	100%

	Protein-coding genes					
	BUSCO groups			Percentage		
	84K	Subgenome A	Subgenome G	84K	Subgenome A	Subgenome G
Complete BUSCOs	1,409	1,388	1,388	96.39%	96.39%	96.39%
Complete and single-copy BUSCOs	58	679	678	4.03%	47.15%	47.08%
Complete and duplicated BUSCOs	1,351	709	710	49.24%	49.24%	49.31%
Fragmented BUSCOs	13	21	25	0.90%	1.46%	1.74%
Missing BUSCOs	18	31	27	1.25%	2.15%	1.88%
Total BUSCOs groups searched	1,440	1,440	1,440	100%	100%	100%

Supplemental Table S7: Summary statistics of the gene annotation of the "84K" genome.

Feature	Number			Minimum length (bp)			Maximum length (bp)		
	84K	Subgenome A	Subgenome G	84K	Subgenome A	Subgenome G	84K	Subgenome A	Subgenome G
**Protein-coding gene**	66,336	33,166	33,170	153	153	153	317,794	317,794	264,153
**mRNA**	125,265	33,166	33,170	153	153	153	25,568	25,568	18,846
**CDS**	125,265	33,166	33,170	153	153	153	15,519	15,519	15,456
**Exon**	1,001,400	190,591	189,717	1	1	1	21,285	21,285	8,277
**Intron**	876,135	157,425	156,547	21	21	21	190,967	190,967	148,812
**Exons/mRNA**	125,265	33,166	33,170	1	1	1	92	92	87
**AED**	124,568	62,200	62,368	0	0	0	1	1	1

Feature	Median of length (bp)			Mean of length (bp)		
	84K	Subgenome A	Subgenome G	84K	Subgenome A	Subgenome G
**Protein-coding gene**	2,741	2,738	2,744	3994.5	4,027	3,962
**mRNA**	1,828	1,380	1,372	2,107	1,632	1,626
**CDS**	1,077	984	978	1,327	1,217	1,211
**Exon**	136	148	149	263.6	284	284
**Intron**	182	185	186	443.6	453	449
**Exons/mRNA**	6	4	4	8	5.7	5.7
**AED**	0.31	0.31	0.32	0.3	0.3	0.3

Supplemental Table S8: Summary of functional annotation of predicted genes.

Source	Databases	Count	Percentage
**Protein-coding genes**	All	66,336	100.00%
	Annotated	61,456	92.64%
	Unannotated	4,880	7.36%
**interProScan**	PANTHER	53,509	80.66%
	Interpro	48,244	72.73%
	SUPERFAMILY	36,146	54.49%
	Pfam	45,103	67.99%
	Gene3D	38,595	58.18%
	MobiDBLite	25,606	38.60%
	CDD	18,564	27.98%
	Phobius	25,120	37.87%
	SMART	17,503	26.39%
	TMHMM	16,138	24.33%
	Coils	9,185	13.85%
	PRINTS	7,627	11.50%
	TIGRFAM	5,239	7.90%
	PIRSF	3,074	4.63%
**Blat**	Swiss_Prot	42,068	63.42%
	TrEMBL	57,345	86.45%
	NR	57,160	86.17%
	eggNOG	51,574	77.75%
	GO	28,245	42.58%
	A.thaliana	50,286	75.80%
	COG	55,545	83.73%
	KEGG_KO	26,143	39.41%
	KEGG_Pathway	16,107	24.28%
	EC	11,555	17.42%

Supplemental Table S9: Summary of the annotated RNA genes.

Source	Gene Category	Gene Number	Transcript Number
Maker	mRNA	66,336	125,265
Barrnap	rRNA	2,591	2,591
tRNAScan-SE	tRNA	1,243	1,243
Rfam	ncRNA	4,429	4,429

Supplemental Table S10: Summary of the repeat elements annotated in the "84K".

Order	Superfamily	Number			Length (bp)			Percent (%)			Mean length (bp)		
		84K	Subgenome A	Subgenome G	84K	Subgenome A	Subgenome G	84K	Subgenome A	Subgenome G	84K	Subgenome A	Subgenome G
**Classification**													
**Class I (retrotransposons)**													
LTR	Copia	60,558	29,881	30,677	33,006,027	15,900,161	17,105,866	4.040	3.973	4.105	545.032	532.116	557.612
LTR	Gypsy	159,937	74,744	85,193	93,609,375	42,913,575	50,695,800	11.458	10.723	12.164	585.289	574.141	595.070
LTR	unknown	192,211	94,198	98,013	61,425,123	28,780,504	32,644,619	7.519	7.192	7.833	319.571	305.532	333.064
nonLTR	LINE_element	4,525	2,171	2,354	3,529,221	1,704,073	1,825,148	0.432	0.426	0.438	779.938	784.925	775.339
nonLTR	pararetrovirus	115	59	56	89,220	46,659	42,561	0.011	0.012	0.010	775.826	790.831	760.018
**Class II (DNA transposons) - Subclass 1**													
DNA	DTA	17,772	9,081	8,691	10,343,550	3,471,345	6,872,205	1.266	1.717	1.649	582.014	382.265	790.727
DNA	DTC	39,914	19,768	20,146	15,696,100	7,807,815	7,888,285	1.921	1.971	1.893	393.248	394.972	391.556
DNA	DTH	10,061	5,036	5,025	3,219,093	1,595,802	1,623,291	0.394	0.406	0.390	319.958	316.879	323.043
DNA	DTM	42,767	21,318	21,449	72,998,041	38,156,882	34,841,159	8.935	8.706	8.360	1706.878	1789.890	1624.372
DNA	DTT	5,144	2,583	2,561	1,632,179	814,125	818,054	0.200	0.204	0.196	317.298	315.186	319.428
DNA	Helitron	247,042	128,014	119,028	69,049,820	35,541,525	33,508,295	8.452	8.373	8.040	279.506	277.638	281.516
TIR	EnSpm_CACTA	320	173	147	81,296	43,726	37,570	0.010	0.009	0.009	254.050	252.751	255.578
TIR	MuDR_Mutator	787	408	379	212,671	110,300	102,371	0.026	0.026	0.025	270.230	270.343	270.108
TIR	PIF-Harbinger	477	234	243	465,616	214,463	251,153	0.057	0.063	0.060	976.134	916.509	1033.551
TIR	hAT	564	274	290	287,068	135,355	151,713	0.035	0.038	0.036	508.986	493.996	523.148
MITE	DTA	3,381	1,718	1,663	615,462	306,718	308,744	0.075	0.077	0.074	182.035	178.532	185.655

MITE	DTC	856	426	430	217,614	108,660	108,954	0.027	0.027	0.026	254.222	255.070	253.381
MITE	DTH	857	441	416	206,546	108,454	98,092	0.025	0.025	0.024	241.011	245.927	235.798
MITE	DTM	1,535	772	763	280,266	143,585	136,681	0.034	0.034	0.033	182.584	185.991	179.136
MITE	DTT	93	50	43	24,008	12,342	11,666	0.003	0.003	0.003	258.151	246.840	271.302
**Repeat elements**													
Simple repeat	Simple repeat	265,100	134,015	131,085	10,984,971	5,512,115	5,472,856	1.345	1.377	1.313	41.437	41.131	41.750
Low complexity	Low complexity	56,880	28,367	28,513	2,795,375	1,389,601	1,405,774	0.342	0.347	0.337	49.145	48.987	49.303
Unknown	Unknown	91,278	44,785	46,493	29,637,512	13,964,748	15,672,764	3.628	3.490	3.761	324.695	311.818	337.099
**Total**	**-**	**1,202,174**	**598,516**	**603,658**	**410,406,154**	**198,782,533**	**211,623,621**	**50.236**	**49.672**	**50.779**	**341.387**	**332.126**	**350.569**

Supplemental Table S11: Annotated transcription factors (TF) gene families in the “84K” genome.

List	TF	Number		
		84K	Subgenome A	Subgenome G
1	MYB	341	172	169
2	bHLH	325	163	162
3	ERF	311	155	156
4	NAC	281	141	140
5	C2H2	244	120	124
6	MYB_related	182	92	90
7	WRKY	175	87	88
8	GRAS	154	75	79
9	bZIP	146	72	74
10	B3	143	78	65
11	HD-ZIP	112	56	56
12	LBD	103	51	52
13	Trihelix	100	50	50
14	C3H	99	51	48
15	G2-like	95	46	49
16	M-type_MADS	84	47	37
17	Dof	80	41	39
18	FAR1	77	39	38
19	GATA	73	36	37
20	MIKC_MADS	65	30	35
21	ARF	61	31	30
22	TCP	56	28	28
23	HSF	54	26	28
24	TALE	52	25	27
25	SBP	51	27	24
26	AP2	43	21	22
27	DBB	35	18	17
28	WOX	35	18	17
29	ZF-HD	34	18	16
30	HB-other	29	13	16
31	Nin-like	29	15	14
32	GRF	27	15	12
33	NF-YC	26	13	13
34	CO-like	22	12	10
35	CPP	22	11	11
36	ARR-B	21	11	10
37	NF-YA	21	11	10
38	NF-YB	21	10	11
39	BES1	20	10	10
40	BBR-BPC	19	10	9

List	TF	Number		
		84K	Subgenome A	Subgenome G
**41**	E2F/DP	18	9	9
**42**	SRS	18	9	9
**43**	YABBY	18	9	9
**44**	CAMTA	12	6	6
**45**	GeBP	12	6	6
**46**	LSD	10	5	5
**47**	VOZ	8	4	4
**48**	EIL	7	4	3
**49**	HB-PHD	7	4	3
**50**	RAV	7	3	4
**51**	NF-X1	6	3	3
**52**	Whirly	6	3	3
**53**	STAT	3	2	1
**54**	HRT-like	2	1	1
**55**	LFY	2	1	1
**56**	NZZ/SPL	2	1	1
**57**	SAP	2	1	1
**58**	S1Fa-like	1	1	0
	Total	141	72	69

Supplemental Table S12: Summary of gene family expansion and contraction in the "84K" genome.

Node	Expansions	Contractions	Rapidly evolving families	Reference
Populus euphratica	2,831	3,369	66	(Ma et al., 2013)
Populus simonii	4,482	3,248	176	(Wu et al., 2020)
Populus trichocarpa	1,568	2,308	148	(Hofmeister et al., 2020)
Populus pruinosa	1,159	4,172	59	(Yang et al., 2017)
Populus ilicifolia	772	5,576	28	(Chen et al., 2020)
Populus deltoides	2,002	4,219	198	(Bai et al., 2021)
Populus tremuloides	2,180	7,147	116	(Lin et al., 2018)
Salix dunnii	1,130	6,260	37	(He et al., 2021)
Salix brachista	1,694	6,076	143	(Chen et al., 2019)
Salix viminalis	3,305	4,321	179	(Almeida et al., 2020)
Salix purpurea	1,843	1,742	199	(Zhou et al., 2020)
Salix suchowensis	3,365	1,929	123	(Dai et al., 2014)
SubgenomeA	937	3,315	57	This study
SubgenomeG	1,043	3,491	65	This study

References:

Ma, T., et al. (2013). Genomic insights into salt adaptation in a desert poplar. *Nature Communications, 4*, 2797. doi:10.1038/ncomms3797

Wu, H., et al. (2020). *De novo* genome asembly of *Populus simonii* further supports that *Populus simonii* and *Populus trichocarpa* belong to different sections. *G3 (Bethesda), 10*, 455-466.

Hofmeister, B. T., et al. (2020). A genome assembly and the somatic genetic and epigenetic mutation rate in a wild long-lived perennial *Populus trichocarpa*. *Genome Biology, 21*, 259.

Yang, W., et al. (2017). The draft genome sequence of a desert tree *Populus pruinosa*. *Gigascience, 6*, 1-7.

Chen, Z., et al. (2020). Survival in the Tropics despite isolation, inbreeding and asexual reproduction: insights from the genome of the world's southernmost poplar (*Populus ilicifolia*). *The Plant Journal, 103*, 430-442.

Bai, S., et al. (2021). Genome Assembly of Salicaceae *Populus deltoides* (Eastern Cottonwood) *I-69* Based on Nanopore Sequencing and Hi-C Technologies. *Journal of Heredity, 112*, 303-310.

Lin, Y. C., et al. (2018). Functional and evolutionary genomic inferences in *Populus* through genome and population sequencing of American and European aspen. *Proceedings of the National Academy of Sciences, 115*, E10970-e10978.
He, L., et al. (2021). Chromosome-scale assembly of the genome of *Salix dunnii* reveals a male-heterogametic sex determination system on chromosome 7. *Molecular Ecology Resources, 21*, 1966-1982.
Chen, J. H., et al. (2019). Genome-wide analysis of Cushion willow provides insights into alpine plant divergence in a biodiversity hotspot. *Nature Communications, 10*, 5230.
Almeida, P., et al. (2020). Genome assembly of the basket willow, *Salix viminalis*, reveals earliest stages of sex chromosome expansion. *BMC Biology, 18*, 78.
Zhou, R., et al. (2020). A willow sex chromosome reveals convergent evolution of complex palindromic repeats. *Genome Biology, 21*, 38.
Dai, X., et al. (2014). The willow genome and divergent evolution from poplar after the common genome duplication. *Cell Research, 24*, 1274-1277.

Supplemental Table S13: Summary of identified structural variations (SVs) between two parental genomes.

Structural annotations			
**Variation_type**	**Count**	**Length (subgenome A)**	**Length (subgenome G)**
**Syntenic regions**	11,433	260,943,737	260,073,721
**Inversions**	244	19,243,972	17,026,775
**Translocations**	6,281	19,148,627	19,227,826
**Duplications (reference)**	16,564	48,311,553	0
**Duplications (query)**	13,082	0	29,153,961
**Not aligned (reference)**	20,748	74,186,898	0
**Not aligned (query)**	24,177	0	83,976,123

Sequence annotations			
**Variation_type**	**Count**	**Length (subgenome A)**	**Length (subgenome G)**
**SNPs**	8,224,933	8,224,933	8,224,933
**Insertions**	792,058	0	6,336,818
**Deletions**	857,031	6,722,805	0
**Copygains**	1,569	0	3,748,478
**Copylosses**	1,383	4,103,830	0
**Highly diverged**	17,690	49,303,304	47,097,008
**Tandem repeats**	136	240,852	273,618

Note:
The subgenome A as reference genome. SNP, single nucleotide polymorphism.
Subgenome A, *P. alba* genome. Subgenome G, *P. tremula* var. *glandulosa* genome.

Supplemental Table S14: Summary of the percentage of methylation sites of CG, CHG and CHH in DNA methylation.

	Group	Type	Number of methylated reads	Number of all reads (methylated reads + unmethylated reads)	Content
84K genome	replicate1	CG	28,451,831	55,854,496	50.94%
		CHG	20,345,067	86,913,855	23.41%
		CHH	14,113,875	451,554,054	3.13%
	replicate2	CG	34,320,914	73,728,708	46.55%
		CHG	27,629,100	128,141,644	21.56%
		CHH	17,878,952	515,441,262	3.47%
	All	CG	62,772,745	129,583,204	48.44%
		CHG	47,974,167	215,055,499	22.31%
		CHH	31,992,827	966,995,316	3.31%
Subgenome A	replicate1	CG	14,173,840	27,991,872	50.64%
		CHG	9,784,514	43,422,767	22.53%
		CHH	6,953,897	224,537,329	3.10%
	replicate2	CG	7,988,950	17,425,810	45.85%
		CHG	6,428,342	31,241,178	20.58%
		CHH	6,036,970	169,453,660	3.56%
	All	CG	22,162,790	45,417,682	48.80%
		CHG	16,212,856	74,663,945	21.71%
		CHH	12,990,867	393,990,989	3.30%
Subgenome G	replicate1	CG	14,277,991	27,862,624	51.24%
		CHG	10,560,553	43,491,088	24.28%
		CHH	7,159,978	227,016,725	3.15%
	replicate2	CG	10,978,973	22,438,719	48.93%
		CHG	8,318,017	37,282,343	22.31%
		CHH	7,334,754	199,093,529	3.68%
	All	CG	25,256,964	50,301,343	50.21%
		CHG	18,878,570	80,773,431	23.37%
		CHH	14,494,732	426,110,254	3.40%

Note:

The two replicates in ONT methylation sequencing were counted separately.

Supplemental Table S15: Categories and number of allelic expression biases between two parental genomes.

	Group	DEG numbers	Dominant expression in subgenome A	Percent	Dominant expression in subgenome G	Percent
**Non-expressing alleles**	-	1,911	-	-	-	-
**Non-differentially expressed genes**	*p*-adjust > 0.05	8,960	-	-	-	-
	All	8,960	-	-	-	-
**Differentially expressed genes**	FC ≤ |2| (*p*-adjust ≤ 0.05)	9,518	4,772	50.14%	4,746	49.86%
	|2| < FC < |8| (*p*-adjust ≤ 0.05)	5,034	2,542	50.50%	2,492	49.50%
	FC ≥ |8| (*p*-adjust ≤ 0.05)	2,607	1,358	52.09%	1,249	47.91%
	All	17,159	8,672	50.54%	8,487	49.46%

Note:

-: data not available.

Supplemental Table S16: 46 features used in the XGBoost machine-learning modeling of allele-specific gene expression (ASE).

Group	Variables
**Methylation**	mCG_gene
	mCHG_gene
	mCHH_gene
	mCG_intron
	mCHG_intron
	mCHH_intron
	mCG_exon
	mCHG_exon
	mCHH_exon
	mCHH_upstream
	mCG_upstream
	mCHG_upstream
	mCG_downstream
	mCHG_downstream
	mCHH_downstream
	mCG_exon_1st,
	mCHG_exon_1st,
	mCHH_exon_1st,
	mCG_intron_1st
	mCHG_intron_1st
	mCHH_intron_1st
**TE**	TE_gene_distance
	TE_unique_number
	TE_shared_number
	TE_occupation_upstream2kb
	TE_occupation_upstream5kb
	TE_occupation_upstream10kb
	TE_occupation_downstream2kb
	TE_occupation_downstream5kb
	TE_occupation_downstream10kb
**Gene sequence**	Gene_length
	Exon_length
	Intron_length
	Ka
	Ks
	Ka/Ks
	TFBS_shared
	TFBS_unique

Group	Variables
**Gene structure**	Exon_number<br>Intron_number<br>TE_length_inside_exon<br>TE_number_inside_exon<br>TE_length_inside_intron<br>TE_number_inside_intron
**Tissue**	Tissue
**Treatment**	Treatment

Supplemental Table S17: Ranking of the 46 features in the XGBoost model (Model 0).

Features	Model 0		
	Gain	Cover	Frequency
mCHG_gene	0.0696	0.0207	0.0207
Ks	0.0432	0.0326	0.0311
Exon_length	0.0415	0.0274	0.0269
mCG_exon	0.0359	0.0313	0.0311
Ka	0.0340	0.0317	0.0305
TE_occupation_upstream10kb	0.0321	0.0379	0.0375
Gene_length	0.0312	0.0244	0.0254
TE_occupation_downstream10kb	0.0310	0.0362	0.0363
mCHH_exon	0.0310	0.0339	0.0333
mCG_gene	0.0306	0.0294	0.0297
mCHG_exon	0.0304	0.0319	0.0313
Ka/Ks	0.0298	0.0278	0.0288
Intron_length	0.0283	0.0229	0.0223
TE_occupation_upstream5kb	0.0278	0.0327	0.0328
mCHH_intron	0.0276	0.0303	0.0294
TE_occupation_downstream5kb	0.0258	0.0293	0.0299
mCHG_intron	0.0249	0.0253	0.0250
mCG_intron	0.0243	0.0248	0.0247
TE_gene_distance	0.0234	0.0291	0.0276
TE_occupation_up2kb	0.0224	0.0275	0.0270
TE_occupation_downstream2kb	0.0206	0.0239	0.0244
mCHH_upstream	0.0200	0.0247	0.0234
TFBS_unique	0.0197	0.0225	0.0235
TFBS_shared	0.0194	0.0225	0.0228
mCHH_downstream	0.0191	0.0238	0.0226
mCHG_downstream	0.0185	0.0225	0.0213
mCHH_gene	0.0184	0.0180	0.0176
mCG_upstream	0.0181	0.0204	0.0196
mCHG_upstream	0.0179	0.0217	0.0207
mCG_downstream	0.0169	0.0200	0.0196
Tissue	0.0165	0.0062	0.0197
mCHH_exon_1st	0.0160	0.0209	0.0192
mCHH_intron_1st	0.0144	0.0196	0.0178
mCG_exon_1st	0.0142	0.0177	0.0167
mCHG_exon_1st	0.0142	0.0187	0.0171
TE_length_inside_intron	0.0133	0.0175	0.0164
TE_length_inside_exon	0.0120	0.0178	0.0162
mCHG_intron_1st	0.0118	0.0174	0.0151
mCG_intron_1st	0.0114	0.0157	0.0142
Treatment	0.0111	0.0040	0.0133

Features	Model 0		
	Gain	Cover	Frequency
Exon_number	0.0095	0.0072	0.0072

Intron_number	0.0053	0.0068	0.0066
TE_number_inside_intron	0.0050	0.0080	0.0073
TE_unique_number	0.0050	0.0051	0.0065
TE_number_inside_exon	0.0034	0.0058	0.0052
TE_shared_number	0.0033	0.0042	0.0048

Note:

Model 0: A XGBoost classification model with 46 predictors (features) and one response with four groups (Diff00, Diff0, Diff2 and Diff8).

Diff00: non-significant difference between a pair of alleles with *p*-adjust > 0.05; Diff0: significant difference between a pair of alleles with *p*-adjust ≤ 0.05 and fold change (FC) ≤ |2|; Diff2: significant difference between a pair of alleles with *p*-adjust ≤ 0.05 and |2| < FC < |8|; Diff8: significant difference between a pair of alleles with *p*-adjust ≤ 0.05 and FC ≥ |8|.

Supplemental Table S18: Ranking of the 15 features in the XGBoost models (Model 1, Model 2 and Model 3).

Features	Model 1			Features	Model 2			Features	Model 3		
	**Gain**	**Cover**	**Frequency**		**Gain**	**Cover**	**Frequency**		**Gain**	**Cover**	**Frequency**
mCHG_gene	0.1267	0.0731	0.0749	Exon_length	0.1203	0.0811	0.0795	*Ks*	0.2306	0.1214	0.1197
Ks	0.1061	0.0948	0.0924	*Ka/Ks*	0.1053	0.0836	0.0869	mCHH_upstream	0.2271	0.0525	0.0574
Ka/Ks	0.0949	0.0831	0.0844	*Ks*	0.1049	0.0945	0.0947	TE_occupation_upstream10kb	0.1273	0.1202	0.1218
TE_occupation_upstream10kb	0.0897	0.1055	0.1017	mCHG_gene	0.0920	0.0697	0.0713	mCHG_gene	0.1171	0.0758	0.0819
TE_occupation_downstream10kb	0.0888	0.1016	0.0994	TE_occupation_upstream10kb	0.0892	0.1046	0.1008	*Ka/Ks*	0.0967	0.1167	0.1159
Exon_length	0.0874	0.0783	0.0770	TE_occupation_downstream10kb	0.0882	0.1035	0.1005	TE_occupation_downstream10kb	0.0564	0.1095	0.1102
Gene_length	0.0837	0.0814	0.0811	Gene_length	0.0747	0.0823	0.0822	TE_gene_distance	0.0482	0.0744	0.0747
mCHH_downstream	0.0679	0.0746	0.0730	mCHH_upstream	0.0734	0.0769	0.0751	Gene_length	0.0366	0.0809	0.0814
mCHH_upstream	0.0676	0.0748	0.0724	mCHH_downstream	0.0701	0.0767	0.0751	mCHH_downstream	0.0320	0.0545	0.0584
TE_gene_distance	0.0635	0.0786	0.0757	TE_gene_distance	0.0637	0.0778	0.0750	Exon_length	0.0168	0.0688	0.0687
TFBS_shared	0.0450	0.0629	0.0598	TFBS_shared	0.0404	0.0600	0.0572	Inter_TE_exon_length	0.0073	0.0284	0.0277
TE_length_inside_exon	0.0386	0.0554	0.0510	Inter_TE_exon_length	0.0383	0.0555	0.0505	TFBS_shared	0.0036	0.0715	0.0605
Tissue	0.0171	0.0110	0.0235	Tissue	0.0163	0.0097	0.0201	TE_shared_number	0.0006	0.0068	0.0086
Treatment	0.0132	0.0101	0.0187	Treatment	0.0141	0.0094	0.0164	Tissue	0.00003	0.0084	0.0060
TE_shared_number	0.0095	0.0150	0.0150	TE_shared_number	0.0093	0.0147	0.0145	Treatment	0.00001	0.0103	0.0071

Notes:

Model 1: A XGBoost classification model with 15 predictors (features) and one response with four groups (Diff00, Diff0, Diff2 and Diff8).

Model 2: A XGBoost classification model with 15 predictors and one response with two groups (group 1 with: Diff0, Diff2 and Diff8; group 2 with: Diff00).

Model 3: A XGBoost regression model with 15 predictors and the difference in expression as the response (in transcripts per million, TPM) of

ASE.

Diff00: non-significant difference between a pair of alleles with *p*-adjust > 0.05; Diff0: significant difference between a pair of alleles with *p*-adjust ≤ 0.05 and fold change (FC) ≤ |2|; Diff2: significant difference between a pair of alleles with *p*-adjust ≤ 0.05 and |2| < FC < |8|; Diff8: significant difference between a pair of alleles with *p*-adjust ≤ 0.05 and FC ≥ |8|.

Supplemental Table S19: Evaluation of the classification XGBoost models (Model 0, Model 1 and Model 2).

Group	Statistics	Model 0	Model 1	Model 2
Training	**Accuracy**	0.7691	0.7665	0.8059
	**Kappa**	0.5857	0.5787	0.6020
	**Mean_F1**	0.6913	0.6859	0.7696
	**Mean_Sensitivity**	0.6619	0.6551	0.7835
	**Mean_Specificity**	0.8886	0.8861	0.8217
	**Mean_Pos_Pred_Value**	0.7392	0.7386	0.7562
	**Mean_Neg_Pred_Value**	0.9110	0.9105	0.8432
	**Mean_Precision**	0.7392	0.7386	0.7562
	**Mean_Recall**	0.6619	0.6551	0.7835
	**Mean_Detection_Rate**	0.1923	0.1916	0.3242
	**Mean_Balanced_Accuracy**	0.7752	0.7706	0.8026
Test	**Accuracy**	0.7416	0.7393	0.7803
	**Kappa**	0.5356	0.5286	0.5489
	**Mean_F1**	0.6525	0.6468	0.7382
	**Mean_Sensitivity**	0.6261	0.6192	0.7481
	**Mean_Specificity**	0.8768	0.8744	0.8030
	**Mean_Pos_Pred_Value**	0.6966	0.6957	0.7284
	**Mean_Neg_Pred_Value**	0.8984	0.8980	0.8186
	**Mean_Precision**	0.6966	0.6957	0.7284
	**Mean_Recall**	0.6261	0.6192	0.7481
	**Mean_Detection_Rate**	0.1854	0.1848	0.3097
	**Mean_Balanced_Accuracy**	0.7514	0.7468	0.7756

Notes:

Model 0: A XGBoost classification model with 46 predictors (features) and one response with four groups (Diff00, Diff0, Diff2 and Diff8)

Model 1: A XGBoost classification model with 15 predictors and one response with four groups (Diff00, Diff0, Diff2 and Diff8)

Model 2: A XGBoost classification model with 15 predictors and one response with two groups (group 1 with: Diff0, Diff2 and Diff8; group 2 with: Diff00).

Diff00: non-significant difference between a pair of alleles with p-adjust > 0.05; Diff0: significant difference between a pair of alleles with p-adjust ≤ 0.05 and fold change (FC) $\leq |2|$; Diff2: significant difference between a pair of alleles with p-adjust ≤ 0.05 and $|2| < FC < |8|$; Diff8: significant difference between a pair of alleles with p-adjust ≤ 0.05 and FC $\geq |8|$.

Supplemental Table S20: Evaluation of the regression XGBoost model (Model 3).

Group	Statistics	Model 3
	RMSE	0.1900
**Training**	R-squared	1.0000
	MAE	0.0830
	RMSE	0.4265
**Test**	R-squared	0.9999
	MAE	0.0933

Note:
Model 3: A XGBoost regression model with 15 predictors and the difference in expression as the response (in transcripts per million, TPM) of ASE.

Supplemental Table S21: Statistics of transcriptome assembly by different methods.

	Genes_number	Genes_total_length (bp)	Genes_mean_length (bp)	Transcript_number
**Trinity.fasta**	122,224	98,958,356	810	324,575
**Trinity-GG.fasta**	196,860	201,249,265	1,022	285,483
**Stringtie.gtf.fa**	75,552	138,877,482	1,838	127,037
**ALL_est.clust.fa**	182,130	245,341,402	1,347	334,176

	Transcript_total_length (bp)	Transcript_mean_length (bp)	Transcript_N50	BUSCO
**Trinity.fasta**	394,889,542	1,217	1,983	C:80.9%[S:26.9%,D:54.0%],F:13.0%,M:6.1%,n:1440
**Trinity-GG.fasta**	395,996,610	1,387	2,456	C:98.2%[S:4.9%,D:93.3%],F:0.6%,M:1.2%,n:1440
**Stringtie.gtf.fa**	252,624,952	1,989	2,477	C:97.7%[S:5.3%,D:92.4%],F:0.7%,M:1.6%,n:1440
**ALL_est.clust.fa**	549,502,968	1,644	2,582	C:97.8%[S:24.0%,D:73.8%],F:1.1%,M:1.1%,n:1440

Supplemental Notes

Supplemental Note S1: 46 features used in the XGBoost machine-learning modeling of allele-specific gene expression (ASE).

**Sequence divergence**	
Gene_length	Difference in gene length between a pair of alleles.
Exon_length	Difference in exon length between a pair of alleles.
Intron_length	Difference in intron length between a pair of alleles.
Ka	Nonsynonymous substitution rate of a pair of alleles.
Ks	Synonymous substitution rate of a pair of alleles.
Ka/Ks	*Ka/Ks* of a pair of alleles.
TFBS_shared	The number of shared TFBS types within 2 kb upstream of a pair of alleles.
TFBS_unique	The number of unique (specific to one allele) TFBS types within 2 kb upstream of a pair of alleles.
**Structural divergence**	
Exon_number	Difference in exon number of a pair of alleles.
Intron_number	Difference in intron number of a pair of alleles.
TE _length_inside_exon	Difference in the total length of TE inserted into exon of a pair of alleles.
TE _number_inside_exon	Difference in the total number of TE inserted into exon of a pair of alleles.
TE _length_inside_intron	Difference in the total length of TE inserted into intron of a pair of alleles.
TE _number_inside_intron	Difference in the total number of TE inserted into intron of a pair of alleles.
**Methylation difference**	
mCG_gene	Difference of CG methylation frequency in the gene body of a pair of alleles.
mCHG_gene	Difference of CHG methylation frequency in the gene body of a pair of alleles.
mCHH_gene	Difference of CHH methylation frequency in the gene body of a pair of alleles.
mCG_exon	Difference of CG methylation frequency in the gene body of a pair of alleles.
mCHG_exon	Difference of CHG methylation frequency in exon of a pair of alleles.
mCHH_exon	Difference of CHH methylation frequency in exon of a pair of alleles.
mCG_intron	Difference of intron CG methylation frequency in intron of a pair of alleles.
mCHG_intron	Difference of CHG methylation frequency in intron of a pair of alleles.
mCHH_intron	Difference of CHH methylation frequency in intron of a pair of alleles.
mCG_upstream	Difference of CG methylation frequency in the upstream 2kb of a pair of alleles.
mCHG_upstream	Difference in CHG methylation frequency in the upstream 2kb of a pair of alleles.
mCHH_upstream	Difference in CHH methylation frequency in the upstream 2kb of a pair of alleles.
mCG_downstream	Difference in CG methylation frequency in the downstream 2kb of a pair of alleles.
mCHG_downstream	Difference in CHG methylation frequency in the downstream 2kb of a pair of alleles.

mCHH_downstream	Difference in CHH methylation frequency in the downstream 2kb of a pair of alleles.
mCG_exon_1st	Difference in CG methylation frequency at the first exon of a pair of alleles.
mCHG_exon_1st	Difference in CHG methylation frequency at the first exon of a pair of alleles.
mCHH_exon_1st	Difference in CHH methylation frequency at the first exon of a pair of alleles.
mCG_intron_1st	Difference in CG methylation frequency at the first intron of a pair of alleles.
mCHG_intron_1st	Difference in CHG methylation frequency at the first intron of a pair of alleles.
mCHH_intron_1st	Difference in CHG methylation frequency at the first intron of a pair of alleles.
**TE occupancy and affinity**	
TE_gene_distance	Difference between the distance to the nearest TE of a pair of alleles.
TE_shared_number	The number of same TE types of a pair of alleles.
TE_unique_number	The number of unique TE types of a pair of alleles.
TE_occupation_upstream2kb	The difference in the length of TE occupation within 2kb upstream of a pair of alleles.
TE_occupation_upstream5kb	The difference in the length of TE occupation within 5kb upstream of a pair of alleles.
TE_occupation_upstream10kb	The difference in the length of TE occupation within 10kb upstream of a pair of alleles.
TE_occupation_downstream2kb	The difference in the length of TE occupation within 2kb downstream of a pair of alleles.
TE_occupation_downstream5kb	The difference in the length of TE occupation within 5kb downstream of a pair of alleles.
TE_occupation_downstream10kb	The difference in the length of TE occupation within 10kb downstream of a pair of alleles.
**Tissue**	
Tissue	The type of tissue to which the sample belongs to.
**Treatment**	
Treatment	Experimental treatment.

Note:

The weighted methylation levels are used to represent the regional methylation levels. Weighted methylation level: total number of reads for all methylated C sites in the region/total coverage in the region. And the illustration on the origin of genetic and epigenetic features can be seen in Figure 4 **(Figure 4)**.

Supplemental Note S2: Library construction and sequencing.

For HiFi sequencing, single-molecule real-time circular consensus sequencing (CCS: https://github.com/PacificBiosciences/ccs) library preparation was performed according to the manufacturer protocols. Briefly, high-quality genomic DNA was extracted from the "84K" leaves using the DNeasy Plant Mini Kit (QIAGEN) and checked for integrity using the Agilent 4200 Bioanalyzer. Subsequently, 8 μg of genomic DNA was sheared using g-TUBEs (Covaris) and purified using AMPure PB magnetic beads. Sequencing libraries were prepared using Pacific Biosciences SMRTbell Template Prep Kit 2.0 according to manufacturer's guidelines and subjected to fragment size selection (fragment molecules ≥ 11 kb) prior to HiFi sequencing on the PacBio Sequel II platform. The generated libraries were sequenced on three SMRT cells.

For ONT sequencing, leaves of the same "84K" individual were collected to extract high-quality genomic DNA using the cetyltrimethylammonium bromide (CTAB) method (Doyle and Doyle, 1987). After purification and quantification, adaptors were ligated using the Genomic DNA Ligation Reaction Kit (SQK-LSK109, Oxford Nanopore Technologies, UK). Then, sequencing libraries with ~20 kb DNA inserts were prepared and sequenced on two flow cells on the Nanopore PromethION platform.

For Hi-C library preparation, we followed a standard procedure (Xie et al., 2015). After the leaf tissues were fixed with formaldehyde and lysed, the cross-linked DNA was digested with MboI restriction enzyme and the digested fragments were biotinylated at the 5' overhangs. The blunt-end fragments were ligated to generate chimeric junctions, which were further purified, physically sheared, and enriched for biotin-containing fragments. Subsequently, the resulting biotinylated DNA fragments were subjected to end-repair, adaptor ligation and polymerase chain reaction, and paired-end sequencing libraries were constructed. These libraries were then sequenced using an Illumina Novaseq 6000 machine with 350 bp fragment.

Supplemental Note S3: Genome assembly and quality assessment.

De novo genome assembly involved the following: primary assembly, Hi-C scaffolding, gap-filling, and optimization (**Supplemental Figure S2**). First, HiFi reads obtained by sequencing in PacBio CCS mode were directly assembled with hifiasm v0.13-r308 (-o hifiasm -t32 -1 mat.yak -2 pat.yak -l 1 ccs.fa) (Cheng et al., 2021). We used a haplotype-switching resolution method, trio-binning which used short reads generated from

parents to bin long reads generated from offspring prior to assembly (Koren et al., 2018). The method has been recommended for development of telomere-to-telomere (T2T) or gapless genome assembly efforts (Nurk et al., 2022).

We then used purge_dups v1.2.5 (default parameters) (Guan et al., 2020) and performed manual controls to ensure that only reads with very shallow depth, very short/long length, high similarity, and a depth of approximately 1/2 the normal depth were removed. The assembly described above yielded the v0.1 version of the "84K" genome (**Supplemental Table S3**). The Hi-C reads were mapped to the v0.1 "84K" genome assembly using Juicer v1.7.6 (https://github.com/aidenlab/juicer) (Durand et al., 2016). We used an automated process to correct, order, and orientation errors by using the 3D-DNA scaffolding pipeline (Dudchenko et al., 2017). Juicebox v11.08 (Durand et al., 2016) was used to fine-tune the assembled scaffolds in a graphical and inter-active fashion through manual adjustments. To further improve the accuracy of the assembly, each chromosome was individually re-scaffolded with 3D-DNA and manually adjusted using Juicebox, including boundary adjustment, removal of incorrect insertions, and alignment adjustment, etc. Subsequently, 38 chromosomes and 10 contigs were created. Gaps were identified and their length was set to 100 bp. Then, the gaps were filled with ONT contigs or ONT ultra-long reads. ONT reads were assembled as contigs using NextDenovo v2.3.0 (genome_size = 450m) (https://github.com/Nextomics/NextDenovo). The obtained contigs were compared with the assembled sequences using unimap (https://github.com/lh3/unimap) to fill the gaps. This step reduced the number of gaps from 55 to 11. The ONT ultra-long reads were further used for the gap filling using tgs-gapcloser v1.1.1 (--minmap_arg '-x map-ont') (Xu et al., 2020b), and the 38 chromosomes with only two gaps were obtained. The two previous "84K" genome assemblies did not cover these gaps (Liu et al., 2019; Qiu et al., 2019). Finally, we further optimized the genome assembly in two ways. First, we polished the assembly a second time using NextPolish v1.1.0 (Hu et al., 2020) with HiFi reads or Illumina short reads. Second, we compared the unplaced contig sequences with chromosome and organelle genome sequences using Redundans v0.14a (Pryszcz and Gabaldon, 2016) to identify redundancies in the unplaced sequences.

In addition, the chloroplast genome (Pt) was assembled based on Illumina short reads using GetOrganelle v1.6.0 (Jin et al., 2020). The complete mitochondrial genome (Mt) was obtained in three steps. First, mitochondria-derived contigs were obtained

from Illumina data using GetOrganelle. Then, HiFi reads were compared with Minimap2 v2.17 (Li, 2018) to obtain mitochondria HiFi reads. Finally, the complete cyclic molecule was assembled with SMARTdenovo (Liu et al., 2021a).

The accuracy and structural completeness of the genome assembly was assessed by the ratio of genome collinearity with other *Populus* species using minimap2. BUSCO (Benchmarking Universal Single-Copy Orthologs) v2.0.1 (lineage dataset: embryophyta_odb9) (Simao et al., 2015) was used to assess genome completeness. In addition, PacBio long reads and Illumina reads were mapped to the genome assembly using Minimap2 v2.17 (Li, 2018) and bwa v0.7.17 (https://github.com/lh3/bwa) (Li, 2013), respectively. Additionally, the transcriptome assembled in the current study was also mapped to the genome assembly using HiSat2 v2.1.0 (https://github.com/infphilo/hisat2) (Kim et al., 2015).

Supplemental Note S4: Gene prediction and functional annotation.

First, protein sequences extracted from *Arabidopsis thaliana* and and 17 Salicaceae species (*Salix brachista*, *S. dunnii*, *S. purpurea*, *S. suchowensis*, *S. viminalis*, *Populus alba*, *P. alba*_var_*pyramidalis*, *P. alba*_x_*P. glandulosa*, *P. davidiana*_x_*P. alba*_var_*pyramidalis*, *P. deltoides*, *P. euphratica*, *P. ilicifolia*, *P. pruinosa*, *P. simonii*, *P. tremula*, *P. tremuloides*, and *P. trichocarpa*) were merged, followed by redundancy removal using CD-HIT v4.6. Subsequently, the assembled transcriptome assembly and the protein sequences were aligned to the repeat-masked reference genome assembly using BLASTn and tBLASTx from BLAST v2.2.28+ (Boratyn et al., 2012), and we further optimized the alignment using Exonerate v2.4.0 (Slater and Birney, 2005). The complete genes identified by BUSCO were used for *ab initio* gene prediction using AUGUSTUS v3.2.3 (Stanke et al., 2008). The gene model prediction using the MAKER v2.31.9 (Cantarel et al., 2008) was finalized using AUGUSTUS v3.2.3 (Stanke et al., 2008). The quality of gene prediction was assessed using the annotation edit distance (AED) for each of the predicted genes as part of MAKER. Non-coding RNA (ncRNA) prediction was performed using specific databases and packages, i.e. barrnap v0.9 (https://github.com/tseemann/barrnap), tRNAscan-SE v2.0 (Lowe and Eddy, 1997), and Rfam database (version 9.1) (http://eggnogdb.embl.de/). In addition, the genomes of the two parent genomes (subgenomes A and G) and 8 Salicaceae species (*Salix purpurea*, *S. suchowensis*, *S. brachista*, *S. dunnii*, *Populus*

ilicifolia, *P. simonii*, *P. deltoides* and *P. trichocarpa*) were used for rDNA annotation with barrnap (--kingdom euk --threads 8). The distribution of rDNAs was mapped using TBtools v1.098769 (Chen et al., 2020a).

Functional annotation of protein-coding genes was performed using three strategies. First, annotation was performed using eggNOG-mapper v2.0.5 (Huerta-Cepas et al., 2017). Protein-coding genes were compared to the eggNOG homologous gene database to annotate gene functions (Jensen et al., 2008). Second, a sequence similarity search was performed for functional annotation. The predicted gene models were aligned with the protein archived in the SwissProt database (Bairoch and Apweiler, 2000), the Translated European Molecular Biology Laboratory (TrEMBL) database (Bairoch and Apweiler, 2000), the NCBI nonredundant protein database (NR), and the Arabidopsis databases using Diamond (Buchfink et al., 2015) to determine the best matching alignments based on E < 1e5 and identity > 30% criteria. Third, domain similarity searches were also performed. Using InterProScan v5.27-66.0 (Jones et al., 2014), motifs and functional domains were determined by searching protein databases, such as PRINTS, Pfam, SMART, PANTHER, and CDD.

Supplemental Note S5: Phylogenetics and gene collinearity in the Salicaceae.

The protein-coding sequences of the two parental genomes (subgenomes A and G) and 12 Salicaceae species (*Salix purpurea*, *S. suchowensis*, *S. brachista*, *S. viminalis*, *S. dunnii*, *Populus euphratica*, *P. pruinosa*, *P. ilicifolia*, *P. simonii*, *P. deltoides*, *P. trichocarpa,* and *P. tremuloides*) were used for comparative genomics **(Supplemental Table S12)**. OrthoFinder2 v2.3.12 was used to construct the orthogroups (Emms and Kelly, 2019). Based on 2,106 single-copy orthologs, we used IQTREE v1.6.7 (Nguyen et al., 2015) to construct a maximum likelihood (ML) phylogenetic tree using the best-fit model (JTT+ F + R4). MAFFT v7.407 (Katoh and Standley, 2013) was used to align homeologs before converting the aligned protein sequences to codon alignment. Concatenated amino acid sequences were trimmed using trimAL v1.4 (Capella-Gutierrez et al., 2009) with -gt 0.8 -st 0.001 -cons 60. The MCMCTree program from PAML v4.9j (Yang, 2007) was used to estimate divergence times under independent substitution rate (clock = 1) setting, with the following details: K80 substitution model, 5e5 iterations, and discarded 1e5 iterations as burn-in, and a fossil date time of 48–52 MYA split time between *Populus* and *Salix* (Chen et al., 2019). CAFÉ v4.2.1 (Han et al., 2013) was then used to infer gene family expansion and contraction based on the

chronogram of the 14 species analyzed.

Gene collinearity analysis on chromosome level in eight species, four willows, four poplars (*Salix purpurea*, *S. suchowensis*, *S. brachista*, *S. dunnii*, *Populus ilicifolia*, *P. simonii*, *P. deltoides,* and *P. trichocarpa*) and the parental subgenomes A and G was performed using MCScanX (Wang et al., 2012).

Supplemental Note S6: Variation between the two parental genomes.

The nucmer alignment tool from the MUMmer toolbox v4.0.0 (Marcais et al., 2018; Zhao et al., 2018) was used to perform whole-genome alignment with the parameter settings "–maxmatch -c 100 -b 500 -l 50 -g 90". Alignment results were filtered by identity (>90) and alignment length (>100). Finally, syntenic regions, structural rearrangements (inversions, translocations, and duplications), and sequence differences (SNPs, indels, etc.) between the two parental genomes were identified using SyRI v1.3 (Goel et al., 2019). The genome of the *P. alba* parent (subgenome A) was used as the reference and subgenome G (from the other parent, *P. tremula* var. *glandulosa*) as the query.

The distance between the inversion region on the chromosome and the nearest TE was also calculated with BEDtools v2.29.2 (Quinlan, 2014). Here, TEs include both randomly generated and observed TEs. Random data were generated using the random function in BEDtools v2.29.2 (Quinlan, 2014). To analyze the effects of structural variants (SVs) on gene expression levels, we calculated the average expression (in TPM) for each gene in the syntenic and the different SV regions across all samples to represent the gene expression levels. The two-sided Wilcoxon test was used to determine the significant differences in gene expression levels between the syntenic and SV regions.

Supplemental Note S7: RNA-seq data and allelic gene expression.

We collected a total of 156 RNA-seq samples of the "84K" clone from different tissues and treatments, each with three independent biological replicates. Low-quality reads and adapters were removed and the remaining reads were quantified for gene expression estimation using Salmon v1.6.0 (--validateMappings --numBootstraps 100) (Patro et al., 2017), through which we obtained count values and normalized TPM values for each sample (**Supplemental Dataset S2**). If the TPM expression value of a gene/allele in TPM exceeded 0.5 in any sample, we considered it to be expressed. Differentially expressed alleles were identified using the DESeq2 package (Love et al., 2014). The

following ranges of fold change (FC) were used as criteria to determine differential expression: 1) no-expression (both alleles were not expressed) (NE); 2) no-significant difference between a pair of alleles with *p*-adjust > 0.05 (Diff00), and 3) significant difference between a pair of alleles with *p*-adjust ≤ 0.05, ASE. The ASE group was further divided into different classes based on the FC in expression: (1) Diff0, when FC ≤ |2| (Diff0); (2) Diff2, when |2| < FC < |8| (Diff2); and (3) Diff8, when FC ≥ |8| (Diff8).

Supplemental Note S8: DNA methylation quantification from ONT long reads.

To quantify DNA methylation (CG, CHG, and CHH) of the "84K" clone, we used DeepSignal-plant v.0.1.4 (Ni et al., 2021), which detects DNA 5mC methylation using a deep learning approach with bidirectional recurrent neural networks (BRNN) and long short-term memory (LSTM) units. In total, we used two replicates of 20× raw ONT read data. First, the raw nanopore were preprocessed by conversion to base sequences using Guppy v5.0.16 (guppy_basecaller, --config dna_r9.4.1_450bps_hac_prom.cfg). The signal data (fast5 format) can be successfully converted into base sequences (fastq format). Then, tombo v1.5.1 (Stoiber et al., 2016) was used to manipulate re-squiggle (Raw Signal Genomic Alignment). Briefly, the re-squiggle algorithm aligned the raw signal (electric current nanopore measurements) to the "84K" genome assembly (tombo resquiggle). Once the data were processed, methylations for the CG, CHG, and CHH contexts were called using DeepSignal-plant under the default reference models (model.dp2.CNN.arabnrice2-1_120m_R9.4plus_tem.bn13_sn16.both_bilstm.epoch6.ckpt). Then, the methylation frequencies of CG, CHG, and CHH sites were generated separately using scripts in the DeepSignal-plant pipeline (https://github.com/PengNi/deepsignal-plant) (**Supplemental Table S14**).

We used the methylation information for each genomic position obtained from DeepSignal-plant to calculate and visualize the average methylation levels of the different regions. At least five reads covering each cytosine methylation site were retained. To map the distribution of methylation levels along chromosomes, a 500 kb sliding window with a step size of 100 kb was defined using the makewindows function in BEDtools v2.29.2 (Quinlan, 2014), and the average methylation level within the window was calculated using methyGff in BatMeth2 (Lim et al., 2012). In addition, the gene body and 2 kb upstream and downstream regions were divided into 100 bins each.

Supplemental Note S9: Feature extraction for machine-learning modeling.

To build the machine-learning (ML) model, a feature dataset was first created. Each column of this dataset represented a feature, and each row represented a pair of alleles in comparison. A total of 46 features were created in six categories (**Supplemental Table S16** and **Supplemental Note S1**). The feature categories were described as follows: (1) Methylation features, including the difference of a pair of alleles in the average methylation frequency (three types: CG, CHG, CHH) of the gene body (mCG_gene, mCHG_gene, mCHH_gene), exons (mCG_exon, mCHG_exon, mCHH_exon), introns (mCG_intron, mCHG_intron, mCHH_intron), sequences from upstream 2 kb (mCG_upstream, mCHG_upstream, mCHH_upstream), sequences of downstream 2 kb (mCG_downstream, mCHG_downstream, mCHH_downstream), first exon (mCG_exon_1st, mCHG_exon_1st, mCHH_exon_1st) and first intron (mCG_intron_1st, mCHG_intron_1st, mCHH_intron_1st). (2) TE occupancy and affinity, a factor of great interest in the study of gene expression. This category includes the distance difference of the closest TE insertion to an allele pair (TE_gene_distance), the number of TE insertion shared by an allele pair (TE_shared_number), the number of unique TE insertion in an allele pair (TE_unique_number), the difference of TE occupancy in upstream 2/5/10 kb of an allele pair (TE_occupation_upstream2kb, TE_occupation_upstream5kb, TE_occupation_upstream10kb), the difference of TE occupancy in downstream 2/5/10 kb of an allele pair (TE_occupation_downstream2kb, TE_occupation_downstream5kb, TE_occupation_ downstream10kb). (3) Sequence divergence in an allele pair, including that of gene length (Gene_length), exon length (Exon_length), intron length (Intron_length), *Ka* (number of substitutions per non-synonymous site), *Ks* (number of substitutions per synonymous site), *Ka*/*Ks*, the number of transcription factor binding sites (TFBS) shared in the upstream 2 kb between alleles (TFBS_shared), the number of TFBS unique in the upstream 2 kb between alleles (TFBS_unique). (4) Structural divergence in a pair of alleles, including the number of exon (Exon_number), the intron number (Exon_number), the number of exons with TE insertion (TE_number_inside_exon), the length of exon with TE insertion (TE_length_inside_exon), the number of introns with TE insertion (TE_number_inside_intron), the length of intron with TE insertion (TE_length_inside_intron). (5) Tissue, the tissue from which the RNA-seq was done. (6) Treatment, the plant's treatment from which the RNA-seq was done.

Supplemental Note S10: Model construction.

Here, XGBoost modeling was used to understand whether allele-specific expression (ASE) was predictable from different genetic, epigenetic features, or experimental designs, and then which factors represented were the most important associations with the observed ASE. Our XGBoost modeling were implemented with R package, xgboost (Chen et al., 2015).

First, XGBoost modeling was performed on a dataset containing 46 features (predictor variables) with the following settings, eta =0.3, gamma = 0.001, max_depth =2, nrounds = 100000, print_every_n = 100, early_stopping_rounds = 200 and default values for other parameters. Our dataset contained 1,220,274 cases in our dataset, 70% of which were used as training, and 30% as test set. Also, we used the same dataset for correlation analysis between features. We kept the features with significant correlation of less than 0.001 ($p < 0.001$). In addition, of the interrelated features, we retained the one that ranked highest in model importance (**Supplemental Figure S19** and **Supplemental Table S17**).

As a primary step, XGBoost modeling (Model 0) was performed with all 46 features as predictors to predict four groups of ASEs (as defined above), which was used to rank the features. After feature selection, a XGBoost model (Model 1) was constructed to predict the four groups of ASEs with 15 selected features. In addition, another XGBoost classification model (Model 2) was created to predict two ASE groups (no ASE; ASE). Another XGBoost regression model (Model 3) was built to predict the difference in expression (in transcripts per million, TPM) of ASE.

To assess the predictability of each classification model, we calculated ROC curves (Receiver Operating characteristic Curves) and AUC (Area Under the Curve) values (Robin et al., 2021). In addition, the modeling results of this purely data-driven approach could be explained using SHapley Additive exPlanations (SHAP) to better interpret the model (default values for parameters) (Lundberg and Lee, 2017). Here, we used SHAP to explain the influence of the five highest-ranking features on the final prediction of ASE.

References:

Bairoch A, Apweiler R (2000) The SWISS-PROT protein sequence database and its supplement TrEMBL in 2000. Nucleic Acids Research **28:** 45-48

Boratyn GM, Schäffer AA, Agarwala R, Altschul SF, Lipman DJ, Madden TL (2012) Domain enhanced lookup time accelerated BLAST. Biology Direct **7:** 1-14

Buchfink B, Xie C, Huson DH (2015) Fast and sensitive protein alignment using DIAMOND. Nature Methods **12:** 59-60

Cantarel BL, Korf I, Robb SM, Parra G, Ross E, Moore B, Holt C, Sanchez Alvarado A, Yandell M (2008) MAKER: an easy-to-use annotation pipeline designed for emerging model organism genomes. Genome Research **18:** 188-196

Capella-Gutierrez S, Silla-Martinez JM, Gabaldon T (2009) trimAl: a tool for automated alignment trimming in large-scale phylogenetic analyses. Bioinformatics **25:** 1972-1973

Chen C, Chen H, Zhang Y, Thomas HR, Frank MH, He Y, Xia R (2020a) TBtools: an integrative toolkit developed for interactive analyses of big biological data. Molecular Plant **13:** 1194-1202

Chen JH, Huang Y, Brachi B, Yun QZ, Zhang W, Lu W, Li HN, Li WQ, Sun XD, Wang GY, He J, Zhou Z, Chen KY, Ji YH, Shi MM, Sun WG, Yang YP, Zhang RG, Abbott RJ, Sun H (2019) Genome-wide analysis of Cushion willow provides insights into alpine plant divergence in a biodiversity hotspot. Nature Communications **10:** 5230

Chen T, He T, Benesty M, Khotilovich V, Tang Y, Cho H, Chen K, Mitchell R, Cano I, Zhou TJRpv- (2015) Xgboost: extreme gradient boosting. R package version 0.4-2 **1:** 1-4

Cheng H, Concepcion GT, Feng X, Zhang H, Li H (2021) Haplotype-resolved *de novo* assembly using phased assembly graphs with hifiasm. Nature Methods **18:** 170-175

Doyle J, Doyle JL (1987) Genomic plant DNA preparation from fresh tissue-CTAB method. Phytochemical Bulletin **19:** 11-15

Dudchenko O, Batra SS, Omer AD, Nyquist SK, Hoeger M, Durand NC, Shamim MS, Machol I, Lander ES, Aiden AP, Aiden EL (2017) *De novo* assembly of the *Aedes aegypti* genome using Hi-C yields chromosome-length scaffolds. Science **356:** 92-95

Durand NC, Robinson JT, Shamim MS, Machol I, Mesirov JP, Lander ES, Aiden EL (2016) Juicebox provides a visualization system for Hi-C contact maps with unlimited zoom. Cell Systems **3:** 99-101

Emms DM, Kelly S (2019) OrthoFinder: phylogenetic orthology inference for comparative genomics. Genome Biology **20:** 238

Goel M, Sun H, Jiao WB, Schneeberger K (2019) SyRI: finding genomic rearrangements and local sequence differences from whole-genome assemblies. Genome Biology **20:** 277

Guan D, McCarthy SA, Wood J, Howe K, Wang Y, Durbin R (2020) Identifying and removing haplotypic duplication in primary genome assemblies. Bioinformatics **36:** 2896-2898

Han MV, Thomas GW, Lugo-Martinez J, Hahn MW (2013) Estimating gene gain and loss rates in the presence of error in genome assembly and annotation using CAFE 3. Molecular Biology and Evolution **30:** 1987-1997

Hu J, Fan J, Sun Z, Liu S (2020) NextPolish: a fast and efficient genome polishing tool for long-read assembly. Bioinformatics **36:** 2253-2255

Huerta-Cepas J, Forslund K, Coelho LP, Szklarczyk D, Jensen LJ, von Mering C, Bork P (2017) Fast genome-wide functional annotation through orthology assignment by eggNOG-Mapper. Molecular Biology and Evolution **34:** 2115-2122

Jensen LJ, Julien P, Kuhn M, von Mering C, Muller J, Doerks T, Bork P (2008) eggNOG: automated construction and annotation of orthologous groups of genes. Nucleic Acids Research **36:** D250-254

Jin JJ, Yu WB, Yang JB, Song Y, dePamphilis CW, Yi TS, Li DZ (2020) GetOrganelle: a fast and versatile toolkit for accurate *de novo* assembly of organelle genomes. Genome Biology **21:** 241

Jones P, Binns D, Chang HY, Fraser M, Li W, McAnulla C, McWilliam H, Maslen J, Mitchell A, Nuka G, Pesseat S, Quinn AF, Sangrador-Vegas A, Scheremetjew M, Yong SY, Lopez R, Hunter S (2014) InterProScan 5: genome-scale protein function classification. Bioinformatics **30:** 1236-1240

Katoh K, Standley DM (2013) MAFFT multiple sequence alignment software version 7: improvements in performance and usability. Molecular Biology and Evolution **30:** 772-780

Kim D, Langmead B, Salzberg SL (2015) HISAT: a fast spliced aligner with low memory requirements. Nature Methods **12:** 357-360

Koren S, Rhie A, Walenz BP, Dilthey AT, Bickhart DM, Kingan SB, Hiendleder S, Williams JL, Smith TPL, Phillippy AM (2018) *De novo* assembly of haplotype-resolved genomes with trio binning. Nature Biotechnology **36:** 1174–1182

Li H (2013) Aligning sequence reads, clone sequences and assembly contigs with BWA-MEM. arXiv:1303.3997

Li H (2018) Minimap2: pairwise alignment for nucleotide sequences. Bioinformatics **34:** 3094-3100

Lim JQ, Tennakoon C, Li G, Wong E, Ruan Y, Wei CL, Sung WK (2012) BatMeth: improved mapper for bisulfite sequencing reads on DNA methylation. Genome Biology **13:** R82

Liu H, Wu S, Li A, Ruan J (2021a) SMARTdenovo: a *de novo* assembler using long noisy reads. GigaByte **2021:** 1-9

Liu YJ, Wang XR, Zeng QY (2019) *De novo* assembly of white poplar genome and genetic diversity of white poplar population in Irtysh River basin in China. Science China Life Sciences **62:** 609-618

Love MI, Huber W, Anders S (2014) Moderated estimation of fold change and dispersion for RNA-seq data with DESeq2. Genome Biology **15:** 550

Lowe TM, Eddy SR (1997) tRNAscan-SE: a program for improved detection of transfer RNA genes in genomic sequence. Nucleic Acids Research **25:** 955-964

Lundberg SM, Lee SI (2017) A unified approach to interpreting model predictions. In Proc. 31st Int. Conf. Neural Information Processing Systems **vol. 30:** 4768–4777

Marcais G, Delcher AL, Phillippy AM, Coston R, Salzberg SL, Zimin A (2018) MUMmer4: A fast and versatile genome alignment system. PLoS Computational Biology **14:** e1005944

Nguyen LT, Schmidt HA, von Haeseler A, Minh BQ (2015) IQ-TREE: a fast and effective stochastic algorithm for estimating maximum-likelihood phylogenies. Molecular Biology and Evolution **32:** 268-274

Ni P, Huang N, Nie F, Zhang J, Zhang Z, Wu B, Bai L, Liu W, Xiao CL, Luo F, Wang J (2021) Genome-wide detection of cytosine methylations in plant from Nanopore data using deep learning. Nature Communications **12:** 5976

Nurk S, Koren S, Rhie A, Rautiainen M, Bzikadze AV, Mikheenko A, Vollger MR, Altemose N, Uralsky L, Gershman A, Aganezov S, Hoyt SJ, Diekhans M, Logsdon GA, Alonge M, Antonarakis SE, Borchers M, Bouffard GG, Brooks SY, Caldas GV, Chen NC, Cheng H, Chin CS, Chow W, de Lima LG, Dishuck PC, Durbin R, Dvorkina T, Fiddes IT, Formenti G, Fulton RS, Fungtammasan A, Garrison E, Grady PGS, Graves-Lindsay TA, Hall IM, Hansen NF, Hartley GA, Haukness M, Howe K, Hunkapiller MW, Jain C, Jain M, Jarvis ED, Kerpedjiev P, Kirsche M, Kolmogorov M, Korlach J, Kremitzki M, Li H, Maduro VV, Marschall T, McCartney AM, McDaniel J, Miller DE, Mullikin JC, Myers EW, Olson ND, Paten B, Peluso P, Pevzner PA, Porubsky D, Potapova T, Rogaev EI, Rosenfeld JA, Salzberg SL, Schneider VA, Sedlazeck FJ, Shafin K, Shew CJ, Shumate A, Sims Y, Smit AFA, Soto DC, Sović I, Storer JM, Streets A, Sullivan BA, Thibaud-Nissen F, Torrance J, Wagner J, Walenz BP, Wenger A, Wood JMD, Xiao C, Yan SM, Young AC, Zarate S, Surti U, McCoy RC, Dennis MY, Alexandrov IA, Gerton JL, O'Neill RJ, Timp W, Zook JM, Schatz MC, Eichler EE, Miga KH, Phillippy AM (2022) The complete sequence of a human genome. Science **376:** 44-53

Patro R, Duggal G, Love MI, Irizarry RA, Kingsford C (2017) Salmon provides fast and bias-aware quantification of transcript expression. Nature Methods **14:** 417-419

Pryszcz LP, Gabaldon T (2016) Redundans: an assembly pipeline for highly heterozygous genomes. Nucleic Acids Research **44:** e113

Qiu D, Bai S, Ma J, Zhang L, Shao F, Zhang K, Yang Y, Sun T, Huang J, Zhou Y, Galbraith DW, Wang Z, Sun G (2019) The genome of *Populus alba* x *Populus tremula* var. *glandulosa* clone 84K. DNA Research **26:** 423-431

Quinlan AR (2014) BEDTools: the Swiss-army tool for genome feature analysis. Current Protocols in Bioinformatics **47:** 11.12.11–11.12.34

Robin X, Turck N, Hainard A, Tiberti N, Lisacek F, Sanchez J-C, Müller M, Siegert S, Doering M, Billings Z (2021) Package 'pROC'. *In*. 2012-09-10 09: 34

Simao FA, Waterhouse RM, Ioannidis P, Kriventseva EV, Zdobnov EM (2015) BUSCO: assessing genome assembly and annotation completeness with single-copy orthologs. Bioinformatics **31:** 3210-3212

Slater GS, Birney E (2005) Automated generation of heuristics for biological sequence comparison. BMC Bioinformatics **6:** 31

Stanke M, Diekhans M, Baertsch R, Haussler D (2008) Using native and syntenically mapped cDNA alignments to improve *de novo* gene finding. Bioinformatics **24:** 637-644

Stoiber M, Quick J, Egan R, Lee JE, Celniker S, Neely RK, Loman N, Pennacchio LA, Brown J (2016) *De novo* identification of DNA modifications enabled by genome-guided nanopore signal processing. BioRxiv**:** 094672

Wang Y, Tang H, Debarry JD, Tan X, Li J, Wang X, Lee TH, Jin H, Marler B, Guo H, Kissinger JC, Paterson AH (2012) MCScanX: a toolkit for detection and evolutionary analysis of gene synteny and collinearity. Nucleic Acids Research **40:** e49

Xie T, Zheng JF, Liu S, Peng C, Zhou YM, Yang QY, Zhang HY (2015) *De novo* plant genome assembly based on chromatin interactions: a case study of *Arabidopsis thaliana*. Molecular

Plant **8:** 489-492

Xu M, Guo L, Gu S, Wang O, Zhang R, Peters BA, Fan G, Liu X, Xu X, Deng L, Zhang Y (2020b) TGS-GapCloser: a fast and accurate gap closer for large genomes with low coverage of error-prone long reads. Gigascience **9:** giaa094

Yang Z (2007) PAML 4: phylogenetic analysis by maximum likelihood. Molecular Biology and Evolution **24:** 1586-1591

Zhao H, Wang S, Wang J, Chen C, Hao S, Chen L, Fei B, Han K, Li R, Shi C, Sun H, Wang S, Xu H, Yang K, Xu X, Shan X, Shi J, Feng A, Fan G, Liu X, Zhao S, Zhang C, Gao Q, Gao Z, Jiang Z (2018) The chromosome-level genome assemblies of two rattans (*Calamus simplicifolius* and *Daemonorops jenkinsiana*). Gigascience **7:** 1-11
