# Supplementary material for: High-quality genome assembly enables prediction of allele-specific gene expression in hybrid poplar
Source: Plant Physiol. 2024 Feb 27;195(1):652–70. doi: 10.1093/plphys/kiae078 (PMC11060683; doi:10.1093/plphys/kiae078)
Supplement: kiae078_Supplementary_Data [file pp.01090.2023-s04.pdf › pp.01090.2023-s04.pdf]

Plant Physiology®

Accurate prediction of allele-specific gene expression in hybrid poplar facilitated by gapless genome assembly

TianLe Shi, Kai-Hua Jia, YuTao Bao, Shuai Nie, XueChan Tian, XueMei Yan, ZhaoYang Chen, ZhiChao Li, Shiwei Zhao, HaiYao Ma, Ye Zhao, Xiang Li, Rengang Zhang, Jing Guo, Wei Zhao, Yousry El-Kassaby, Niels Müller, Yves Van de Peer, Xiao-Ru Wang, Nathaniel Street, Ilga Porth, Xinmin An, and Jian-Feng Mao

Review Timeline:

Submission Date:	20-Jul-2023
Editorial Decision:	13-Sep-2023
Revision Received:	13-Dec-2023
Editorial Decision:	30-Dec-2023
Revision Received:	08-Jan-2024
Accepted:	09-Jan-2024

12-Sep-2023

Mr. Jian-Feng Mao
Umea Universitet
Department of Plant Physiology
Umea 90736
China

RE: Accurate prediction of allele-specific gene expression in hybrid poplar facilitated by gapless genome assembly

Dear Dr. Mao:

We have received reviews of your manuscript, which you may view at the end of this letter. Based on the advice received and my evaluation, I believe that, pending revision, this article may ultimately be acceptable for publication in Plant Physiology.

First, as noted by Reviewer 1, RNA-seq of the 84K parents would be necessary to support any claims related to allele-specific expression. Second, the abstract and result sections should plainly and explicitly state the model's accuracy. Thirdly, please follow the request by Reviewer 2 to remove the word "gapless" from the title and ensure that the presence of gaps is clearly described in the results section. Lastly, please proofread the document again and discuss the references suggested by Reviewer 1 and any other recently published poplar genome assemblies.

When you are ready to submit your revision, click the below link and find the red arrow under your Author Tasks heading for this revision submission.

If you want to visit this version of the manuscript, you may click the link below and then click on the folder that reads "Post Decision Manuscripts".

Link Not Available

When uploading the revised version of this article, please be sure to include as complete an answer as possible to the referees' criticisms during the first round of reviews in the "response to reviewers" section.

Plant Physiology considers a revision for up to 60 days after a manuscript has been provisionally accepted; limited extension of this timeframe is at the discretion of the Monitoring Editor. A reminder notice will be sent to you 2 weeks before the deadline, at which time you may choose to contact the editorial office to request an extension; if so, please provide a justification for your request. After the expiration of this deadline, the article would need to be submitted as a resubmission and would be handled as such.

Thank you very much for giving us an opportunity to review this work. I look forward to receiving the next version.

Sincerely,

Plant Physiology Board of Editors

Want to add this revision deadline to your calendar? Click below!

-- Reviewer comments:
Reviewer #1 (Comments for the Author):

The manuscript entitled "Accurate prediction of allele-specific expression (ASE) in hybrid poplar facilitated by a gapless genome assembly" submitted by Shi et al. used various in silico methods to identify genomic features attributable to ASE in the hybrid poplar clone "84K". The authors sequenced and annotated haplotypes of the hybrid genome, and compared four ASE categories, which the authors defined based on magnitude, across tissues. The authors found that "gene body methylation" was

the most important contributing factor to ASE. However, RNA of "84K" parents was not sequenced to test for differential expression, thus regulatory effects (cis, trans, etc.) were not performed since it is not possible to infer from "84K" expression data alone. Nevertheless, the authors performed sound bioinformatic practices using industry-standard software and described their methods well. Overall, the manuscript is well-written and will likely garner broad interest from the plant genetics research community.

Comments:

P4 L85: The authors reference poplar articles here but note reports of heterosis in both poplar and willow species hybrids. Below is a list of relevant papers on interspecific heterosis in willow:
• Serapiglia et al. (2014) Ploidy-level affects important biomass traits of novel shrub willow (Salix) hybrids. https://doi.org/10.1007/s12155-014-9521-x
• Fabio et al. (2016) Genotype × environment interaction analysis of North American shrub willow yield trials confirms superior performance of triploid hybrids. https://doi.org/10.1111/gcbb.12344
• Carlson et al. (2017) Dominance and sexual dimorphism pervade the Salix purpurea L. transcriptome. https://doi.org/10.1093/gbe/evx174
• Carlson and Smart (2021) Heterosis for biomass-related traits in novel triploid hybrids of willow (Salix spp.). https://doi.org/10.1007/s12155-021-10305-0
• Carlson et al. (2022) Nonadditive gene expression is correlated with nonadditive phenotypic expression in interspecific triploid hybrids of willow (Salix spp.). https://doi.org/10.1093/g3journal/jkab436

P4 L90. "...fused hybrid..." is redundant - omit "fused".

P4 L95. As in P4 L85, there have been relevant papers in the Salicaceae on ASE, for instance, the Carlson et al. (2022) G3 paper also uses regression/ML approaches to predict heterosis from ASE and DE, whereas in the present manuscript, the authors focus on gene "features", so it would be worth introducing here or later in the discussion, especially since both studies identified structural variation between the parents using whole genome data (notably, willow chromosome 9 as in this study).

P12 L325. Regarding ASE groups, why were fold-changes of <2, >2 and <8, and >8 considered instead of significance values (p>0.05, p<0.05, p<0.005, etc.)? These groups are categorical, correct? Also, when reporting p-values, did the authors use an adjusted p-value (i.e. q-value) to define these groups (this is noted in: P26 L773)?

P14 L 401. Expound upon "complex relationship" related to effects of TE occupancy - in what way (if known or not, a hypothesis)? Additional discussion would help the reader here since TE occupancy was considered an important feature in ASE.

P14 L411. Please expand upon "...[ROC] values confirmed the good performance...". Statistically, more effort could be made to interpret the prediction results.

P16 L448. "...synteny regions" [sic] "syntenic regions"

P16 L450. Abbreviation of "structural variants (SV)" earlier (i.e. P5 L116) is fine, repeating is not necessary. Check this for other abbreviations throughout.

P16 L469. This was also discussed (ASE/heterosis) in willow hybrids, including GO enrichment of "stress response" for genes with strong ASE, see ref #5 in first comment.

P17 L495. Since only one hybrid was investigated and poplar is highly heterozygous, do the authors expect there to be substantial differences in ASE results among >1 F1 progeny from the same cross (differential inheritance of SV's etc.)?

P27 L795. The genome is not publicly available. After visiting the Genome Warehouse link, I searched for PRJCA010836 but was led to a page with the following: "The genome assembly you tried to retrieve has not been released. Release date: 2025-06-10." It is generally good to have all data as publicly accessible prior to publication. Is there a reason the authors want the genome embargoed until 2025-06-10? Check with Editor for best practices.

Reviewer #2 (Comments for the Author):

Overall:
Carefully comb through the document for grammatical errors. There are several sentences which could be rewritten more concisely, however, the overall document is well-written and easy to read.

Line 66: It seems the sentence was truncated.
Current: "No transcription bias at the subgenome level was uncovered, but extensive expression level differences between alleles."

Suggested: "No transcription bias at the subgenome level was uncovered, but extensive expression level differences between alleles were detected." or similar wording to finish the sentence.

Line 178: "Gap-less" is used to describe the haplotype P. alba assembly which contains two gaps in Chr9. To me this implies the assembly contains no gaps, so I would consider removing the term gap-less. "...and a haplotype assembly of P. alba containing only two small gaps" would work fine.

In Supp. Table 4, there are columns titled "Percent" which contain no values other than asterisks indicating the values are unavailable. Do these columns show something or could they be removed?

The genome assembled with long-read data does not increase in BUSCO score from previous short read genomes, however, it may be worth using a quality metric for the repeat space of the assembly, such as the LTR Assembly Index (LAI), because the long read genome could potentially improve assembly of highly repetitive regions. LAI can be calculated using the EDTA pipeline which was already used in this project.

Line 444: Are you able to confirm the existence of the inversions in the predicted centromeres?

Lines 455-458: "We hypothesize that the accumulation of TEs may lead to chromosome inversions, and that these regions promoted methylation modifications, which in turn affected gene expression levels to some extent." Could you recommend a method to validate or explore this hypothesis in the future?

Line 552: "We used an automated process to correct, order, and orientation errors by using the 3D-DNA scaffolding pipeline (Dudchenko et al., 2017)." Is this supposed to say "We used an automated process to correct and order orientation errors by using the 3D-DNA scaffolding pipeline (Dudchenko et al., 2017)."?

Line 636: Period missing between phylogenetic tree and MAAFT.

Supp. Table 21: Title has a typo. Mechine learning instead of Machine learning. Carefully scan through all documents for such typos.

Line 742: "70% of which were used as training, and 30% as test set." Considering revising to, 70% of which were used for training and 30% for testing.

It may improve the interpretability for researchers who are less familiar with ML to quantify the accuracy of the prediction model in more plain language. "Our model achieved X% accuracy, correctly classifying ASE in Y examples".

##

Reviewer #1 (Comments for the Author):

The manuscript entitled "Accurate prediction of allele-specific expression (ASE) in hybrid poplar facilitated by a gapless genome assembly" submitted by Shi et al. used various in silico methods to identify genomic features attributable to ASE in the hybrid poplar clone "84K". The authors sequenced and annotated haplotypes of the hybrid genome, and compared four ASE categories, which the authors defined based on magnitude, across tissues. The authors found that "gene body methylation" was the most important contributing factor to ASE. However, RNA of "84K" parents was not sequenced to test for differential expression, thus regulatory effects (cis, trans, etc.) were not performed since it is not possible to infer from "84K" expression data alone. Nevertheless, the authors performed sound bioinformatic practices using industry-standard software and described their methods well. Overall, the manuscript is well-written and will likely garner broad interest from the plant genetics research community.

Response: We would like to express our gratitude to the reviewers for their careful evaluation of our manuscript. We appreciate the thoughtful comments and feedback provided.

We acknowledge the reviewer's recognition of the various in silico methods we employed to identify genomic features associated with ASE in the hybrid poplar clone "84K". Our aim was to shed light on the complexities of allele-specific expression and its underlying genomic attributes in this specific system. We are pleased that the reviewer found our methodology well-described and our use of industry-standard software sound.

The reviewer astutely points out that while we identified "gene body methylation" as a significant contributing factor to ASE, we did not perform regulatory effect analyses, such as *cis* and *trans* effects, due to the absence of the parent lines, and the unavailability of RNA sequencing data from the "84K" parents. We appreciate this observation, and it is indeed a limitation of our study. We acknowledge that regulatory effects are crucial in understanding the mechanisms underlying ASE fully.

Once again, we extend our sincere thanks to the reviewers for their time and thoughtful assessment of our manuscript. We will consider their feedback as we continue to refine and enhance our research.

Comments:

1. P4 L85: The authors reference poplar articles here but note reports of heterosis in both poplar and willow species hybrids. Below is a list of relevant papers on interspecific heterosis in willow:
• Serapiglia et al. (2014) Ploidy-level affects important biomass traits of novel shrub willow (Salix) hybrids. https://doi.org/10.1007/s12155-014-9521-x
• Fabio et al. (2016) Genotype × environment interaction analysis of North American shrub willow yield trials confirms superior performance of triploid hybrids.

https://doi.org/10.1111/gcbb.12344

• Carlson et al. (2017) Dominance and sexual dimorphism pervade the Salix purpurea L. transcriptome. https://doi.org/10.1093/gbe/evx174

• Carlson and Smart (2021) Heterosis for biomass-related traits in novel triploid hybrids of willow (Salix spp.). https://doi.org/10.1007/s12155-021-10305-0

• Carlson et al. (2022) Nonadditive gene expression is correlated with nonadditive phenotypic expression in interspecific triploid hybrids of willow (Salix spp.). https://doi.org/10.1093/g3journal/jkab436

Response: We appreciate the reviewer's valuable input and the list of relevant papers on interspecific heterosis in willow hybrids. These references have enriched our understanding of heterosis in related plant species and have contributed to the depth of our research. In response to the reviewer's recommendations, we have carefully read and cited the suggested articles in this revised manuscript (lines 84 to 88, 475-478 and 519-522), ensuring proper acknowledgment of the relevant literature. We are grateful for the reviewer's contribution to improving our manuscript and believe that including these additional references strengthens the context and relevance of our research within the broader scientific community.

2. P4 L90. "...fused hybrid..." is redundant - omit "fused".

Response: Thank you for your suggestion. We have replaced "...fused hybrid..." with "...hybrid...", in line 93 in this revised manuscript. All changes are highlighted.

3. P4 L95. As in P4 L85, there have been relevant papers in the Salicaceae on ASE, for instance, the Carlson et al. (2022) G3 paper also uses regression/ML approaches to predict heterosis from ASE and DE, whereas in the present manuscript, the authors focus on gene "features", so it would be worth introducing here or later in the discussion, especially since both studies identified structural variation between the parents using whole genome data (notably, willow chromosome 9 as in this study).

Response: Thank you for your review comments on our manuscript. We have provided additional notes in the Introduction (lines 84-85) and Discussion section (lines 475-478 and 519-522) of the revised manuscript based on your suggestions.

We have emphasized that the use of regression and machine learning in willow has demonstrated that the differential expression genes of parents are effective in predicting heterosis, providing theoretical support for hybrid selection based on genetic expression (lines 519-522). We believe that this supplement will better demonstrate the links and differences with previous studies and provide readers with more comprehensive background information. Thank you again for your valuable comments.

4、P12 L325. Regarding ASE groups, why were fold-changes of <2, >2 and <8, and >8 considered instead of significance values (p>0.05, p<0.05, p<0.005, etc.)? These groups are categorical, correct? Also, when reporting p-values, did the authors use an adjusted p-value (i.e. q-value) to define these groups (this is noted in: P26 L773)?

Response:

(1) In our study, the choice of using fold-changes as categories (i.e., <2, >2 and <8, >8) instead of significance values (such as p>0.05, p<0.05, p<0.005, etc.) is based on the primary objective of our research. We aimed to explore the expression patterns of alleles with different expression fold changes. In the analysis of Allele-Specific Expression (ASE), the use of fold-changes (such as <2, >2, <8, and >8) is a common method in gene expression research. This is because they provide a clear benchmark to identify varying degrees of gene expressional differences, and they offer a more intuitive and biologically meaningful interpretation of the data. In the context of ASE, they facilitate the identification of differentially expressed alleles between parental species in hybrid offspring. In addition, when analyzing differentially expressed alleles, we not only consider the fold-changes, but also use p-adjust to ensure the highlighting of genes with substantial and statistically significant expression changes.

(2) Yes, these groups (<2, >2 and <8, and >8) are indeed categorical. They were established to facilitate a clearer interpretation of the data by separating the genes into categories according to the magnitude of the observed change in gene expression.

(3) We would like to clarify that in our gene differential expression computation with DESeq2, we used the adjusted *p*-value (also known as *q*-value), actually. In the revised manuscript, we replace "*p*" in the original manuscript with "*p*-adjust" (adjusted *p*-value). This adjustment is important to control the false discovery rate and to account for multiple testing. We apologize for any previous confusion, and thank you for pointing out the clarification needed.

5. P14 L 401. Expound upon "complex relationship" related to effects of TE occupancy - in what way (if known or not, a hypothesis)? Additional discussion would help the reader here since TE occupancy was considered an important feature in ASE.

Response:

(1) Based on Figure 5B, we have identified that TE occupancy in the upstream/downstream 10 kb region has a specific and significant impact on ASE. Subsequently, we aimed to investigate further clues on whether these TEs activate or suppress the expression of these allele pairs. However, we did not observe a clear linear correlation between these two, in other words, TE occupancy does not simply relate to the expression of genes categorized as high or low in the allele pairs from Figure 5E. This indicates that TE occupancy's effect on differential expression of allele pairs is complex.

As discussed in the Introduction section (lines 102-121, ASE is regulated by genetic factors (such as TEs and *cis*-regulatory elements) as well as epigenetic factors (DNA methylation modification and histone modification).

(2) In addition, we have further discussed the relationship between TE occupancy and ASE in lines 507-516 of the manuscript, hoping to provide more insights to the reader.

We believe that the true relationship between TE occupancy and ASE is more complex than we imagined. TEs can carry regulatory sequences which can influence the expression of nearby genes. TEs can cause insertions, deletions, inversions, and other structural variations in the genome. These events can lead to disruption of regulatory elements (both *cis*- and *trans*- elements), potentially causing ASE. The exact mechanism by which TEs influence ASE can be highly context-dependent, varying with the type of TE, its location in the genome, the gene in question, and the broader genomic and cellular environment. Because of this complexity, it is often challenging to predict precisely how TEs will affect ASE without detailed empirical studies. Our study identified revealed an interesting open question for further molecular genetic investigation.

6. P14 L411. Please expand upon "...[ROC] values confirmed the good performance...". Statistically, more effort could be made to interpret the prediction results.

Response: Thank you for your suggestion. We have added descriptions of the accuracy and mean F1 values of the training and test sets for Model 1 and Model 2 in lines 411-415 of the revised manuscript to explain the predictive results. All changes are highlighted.

7. P16 L448. "...synteny regions" [sic] "syntenic regions"

Response: Thank you for your suggestion. We have modified this sentence according to your suggestion, in line 457 in this revised manuscript. The change is highlighted.

8. P16 L450. Abbreviation of "structural variants (SV)" earlier (i.e. P5 L116) is fine, repeating is not necessary. Check this for other abbreviations throughout.

Response: Thank you for your suggestion. We have conducted a comprehensive review of the manuscript and have addressed the issue of repetitive abbreviation usage. We have ensured consistency in abbreviation usage throughout the manuscript, and as a result, the abbreviation "SV" for "structural variants" has been appropriately introduced earlier and consistently applied throughout the text (The legend has not been changed now). All changes are highlighted.

9. P16 L469. This was also discussed (ASE/heterosis) in willow hybrids, including GO enrichment of "stress response" for genes with strong ASE, see ref #5 in first comment.

Response: We have added relevant discussion on ASE/heterosis by referring to the study on willow hybrids (Carlson et al. 2022), in lines 475 to 478 in this revised manuscript. We emphasize the strong allelic-specific expression (ASE) of genes enriched in the "response to stimulus" gene ontology (GO) term identified in

Carlson's paper (Carlson et al. 2022), indicating their potential key roles in heterosis. All changes are highlighted.

10. P17 L495. Since only one hybrid was investigated and poplar is highly heterozygous, do the authors expect there to be substantial differences in ASE results among >1 F1 progeny from the same cross (differential inheritance of SV's etc.)?

Response: Here, we conducted our study specifically on the "84K" hybrid poplar. This specific hybrid, "84K", it exhibits outstanding phenotypic traits such as growth vigor and straightness, and its superiorities as a model clone for genetic manipulations. This "84K" was introduced to China in 1984 and we have no other offsprings from the same or similar cross reachable for this study. Therefore, our study was focused solely on this individual.

Poplar is mostly dioecious (rarely monoecious), and high heterozygosity is expected for most individuals. We will expect to see substantial differences among offsprings from interspecific hybridization. In agreement with this comment from the reviewer, we believe comparison with other progeny is an excellent research strategy.

11. P27 L795. The genome is not publicly available. After visiting the Genome Warehouse link, I searched for PRJCA010836 but was led to a page with the following: "The genome assembly you tried to retrieve has not been released. Release date: 2025-06-10." It is generally good to have all data as publicly accessible prior to publication. Is there a reason the authors want the genome embargoed until 2025-06-10? Check with Editor for best practices.

Response: We made all related datasets (raw reads, assembly, annotation, features for modeling) and codes (modeling, optimization, and evaluation) publicly available in the revision.

##

Reviewer #2 (Comments for the Author):

Overall:

Carefully comb through the document for grammatical errors. There are several sentences which could be rewritten more concisely, however, the overall document is well-written and easy to read.

Response: Thank you for your feedback. We have carefully reviewed the document and made improvements to address grammatical errors and ensure concise writing where possible. We appreciate your positive assessment of the overall readability of the manuscript.

1、Line 66: It seems the sentence was truncated.

Current: “No transcription bias at the subgenome level was uncovered, but extensive expression level differences between alleles.” Suggested: “No transcription bias at the subgenome level was uncovered, but extensive expression level differences between alleles were detected.” or similar wording to finish the sentence.

Response: Thank you for pointing out the sentence truncation issue. We have addressed this by making the necessary modifications to the sentence as suggested, in lines 63-64 of this revised manuscript. All changes are highlighted.

2、Line 178: “Gap-less” is used to describe the haplotype *P. alba* assembly which contains two gaps in Chr9. To me this implies the assembly contains no gaps, so I would consider removing the term gap-less. “...and a haplotype assembly of *P. alba* containing only two small gaps” would work fine.

Response: To eliminate any ambiguity, we have replaced the term “gap-less” in the title with “high-quality”. Additionally, in accordance with your suggestion, we have removed the term “gap-less” from lines 179-180 of the revised manuscript to describe the haplotype assembly of *P. alba*. The revised sentence now reads, “...and a haplotype assembly of *P. alba* containing only two gaps”. Thank you for your suggestion, and we have made these adjustments accordingly. All changes are highlighted.

3. In Supp. Table 4, there are columns titled “Percent” which contain no values other than asterisks indicating the values are unavailable. Do these columns show something or could they be removed?

Response: Thank you for your suggestion. In response to your feedback, we will replace the asterisks in the original table with “-” to indicate the absence of values in the “Percent” columns. This change will provide clarity in the table.

4. The genome assembled with long-read data does not increase in BUSCO score from previous short read genomes, however, it may be worth using a quality metric for the repeat space of the assembly, such as the LTR Assembly Index (LAI), because the long read genome could potentially improve assembly of highly repetitive regions. LAI can be calculated using the EDTA pipeline which was already used in this project.

Response: Yes, we incorporated LAI in this revision, accordingly. However, we have to clarify here that LTR Assembly Index (LAI) is heavily dependent on the quantity of intact LTRs in evaluating genome assembly quality (Raw LAI = (Intact LTR retrotransposon length / Total LTR sequence length) * 100%). Theoretically, as the quality of genome assembly improves, more intact LTRs are assembled, leading to higher LAI scores. However, two factors limit the LAI score. First, as the genome assembly quality enhances, the Total LTR sequence length (the denominator in the formula) also increases. Secondly, if the species has not recently experienced a significant expansion of LTRs, then the number of intact LTRs (the numerator in the formula) in the genome is inherently low. To illustrate, let's consider Subgenome A and Subgenome G as examples: Subgenome G has a raw LAI score of 14.86, whereas Subgenome A has a score of 10.99, indicating a lower LAI for Subgenome A. Upon identifying subgenome-specific *k*-mer sequences (most of which are LTR sequences, as in wheat, where approximately 98.7% of sequences are LTR sequences (Jia et al., 2022)), we observed that Subgenome A possesses fewer subgenome-specific *k*-mer sequences compared to Subgenome G (Figure 1), consistent with its lower LAI scores. Therefore, LAI's effectiveness in assessing genome quality greatly depends on recent LTR expansions.

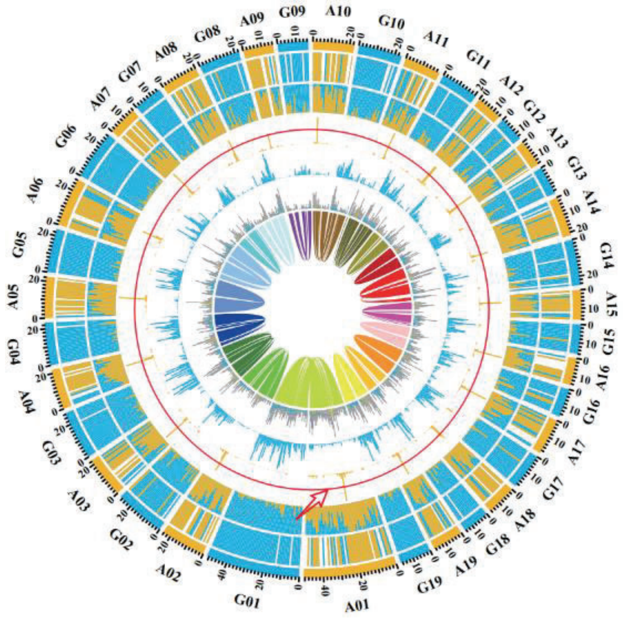

Figure 1. Chromosomal characteristics. From outer to inner circles (1-8): (1) subgenome assignments based on *k*-means algorithm; (2) significant enrichment of subgenome-specific *k*-mers - the same color as the subgenome indicates significant enrichment for those subgenome-specific *k*-mers; white areas are not significantly enriched; (3) normalized proportion (relative) of subgenome-specific *k*-mers; (4-5) count (absolute) of each subgenomespecific *k*-mers set; (6) density of long terminal repeat retrotransposons (LTR-RTs) - if the color is consistent with the subgenome, it indicates that LTR-RTs are significantly enriched to those subgenome-specific *k*-mers; gray indicates nonspecific LTR-RTs; (7) homoeologous blocks. All statistics (2-6) are computed in sliding windows of 1 Mb.

Jia KH, Wang ZX, Wang L …, Zhang RG, Mao JF. SubPhaser: A robust allopolyploid subgenome phasing method based on subgenome-specific *k*-mers. *New Phytologist*, 2022, 235: 801-809

5. Line 444: Are you able to confirm the existence of the inversions in the predicted centromeres?

Response: We went to Hi-C for further support/verification for the existence of inversions. Based on the results of our existing analyses (Figure 2) and the results of Hi-C (Figure 3), it is indicated that there are inversions in the predicted centromeres (as indicated by the arrows). In addition, we compared it with a recently published patchouli genome study (Shen et al., 2022) in our discussion, and we found that this phenomenon is not only in "84K", but it is also present in patchouli. The conclusion is also further illustrated in lines 451-455 of our manuscript. All changes are highlighted.

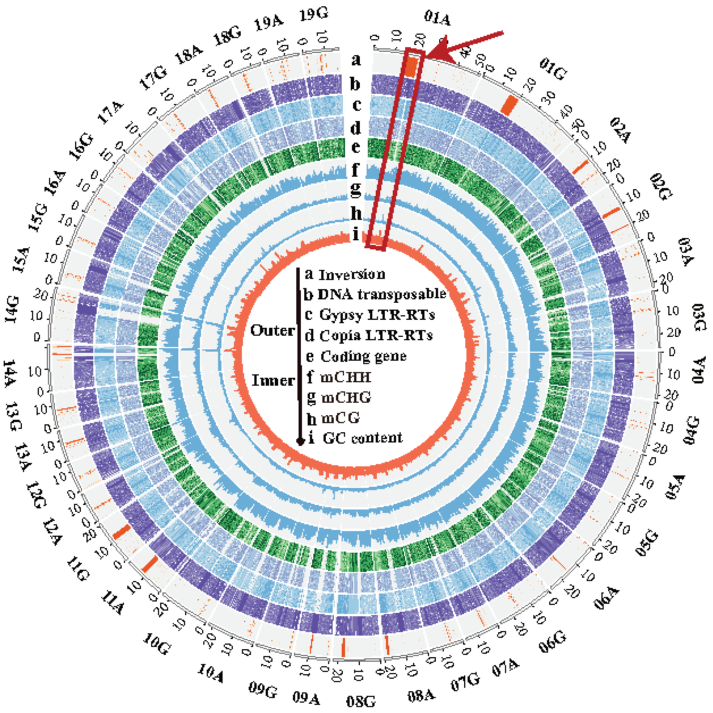

Figure 2. Synteny and distribution of genomic features of the poplar clone "84K". a, density of inversions (or inverted regions). b, distribution of DNA-like transposons. c, distribution of *Gypsy* LTR-RTs. d, distribution of *Copia* LTR-RTs. e, distribution of coding genes. f-h, average methylation levels of CHH, CHG, and CG, respectively. i, histogram of GC content in 50 kb non-overlap sliding windows.

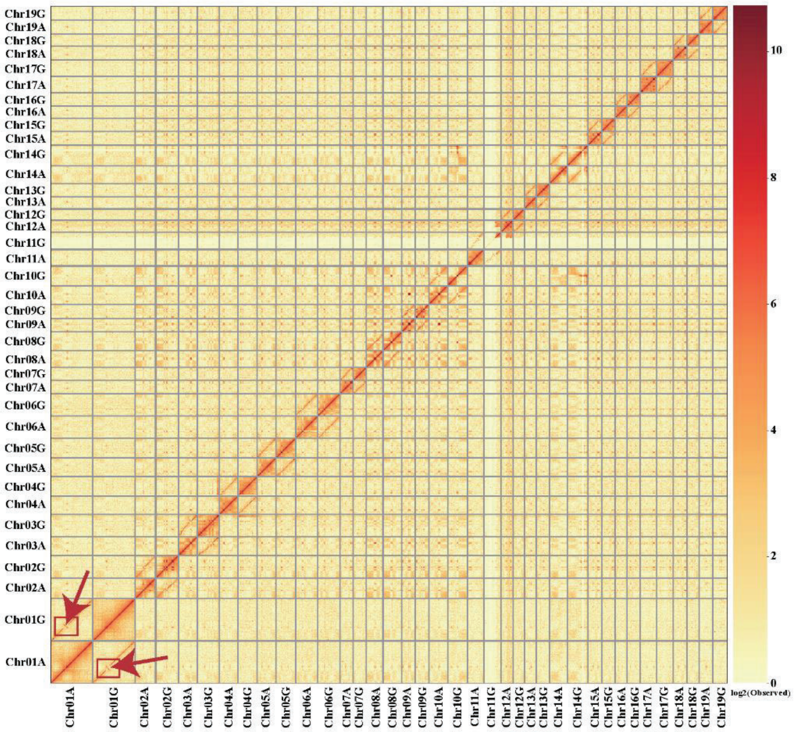

Figure 3. Genome-wide analysis of chromatin interactions in the genome based on Hi-C data.

Shen, Y., et al. (2022). Chromosome-level and haplotype-resolved genome provides insight into the tetraploid hybrid origin of patchouli. Nature Communications. 13, 1-15.

6. Lines 455-458: “We hypothesize that the accumulation of TEs may lead to chromosome inversions, and that these regions promoted methylation modifications, which in turn affected gene expression levels to some extent.” Could you recommend a method to validate or explore this hypothesis in the future?

Response: Yes, tons of thanks. This is a good suggestion. As we know, there are established methods and strategies to tackling the TEs’ involvement in structural variation (sequencing + comparative genomic analysis), and also in investigating methylation modification (several related sequencing techniques). Given the limited space in this article, it is not so easy to figure out a good methodological pathway by comparing different available choices. So, we did not make this recommendation in this revision.

7. Line 552: “We used an automated process to correct, order, and orientation errors by using the 3D-DNA scaffolding pipeline (Dudchenko et al., 2017).” Is this supposed to say “We used an automated process to correct and order orientation errors by using the 3D-DNA scaffolding pipeline (Dudchenko et al., 2017).”?

Response: Yes. We have made modifications to this sentence based on your

suggestion, which are in lines 575 to 576 in the revision manuscript. The revised text is “We used an automated process to correct and order orientation errors by using the 3D-DNA scaffolding pipeline”. All changes are highlighted.

8. Line 636: Period missing between phylogenetic tree and MAAFT.
Response: Done. And the change is highlighted in yellow in line 657.

9. Supp. Table 21: Title has a typo. Mechine learning instead of Machine learning. Carefully scan through all documents for such typos.
Response: Thank you for highlighting the typo in Supplementary Table 21. We have amended the title from “Mechine learning” to “Machine learning”. Additionally, we have conducted a thorough review of the entire manuscript to identify and correct any similar errors. Your attention to detail is greatly appreciated, and we strive to ensure the accuracy and professionalism of our work. And all changes are highlighted.

10. Line 742: “70% of which were used as training, and 30% as test set.” Considering revising to, 70% of which were used for training and 30% for testing.
Response: Done. The revised text is “Our dataset contained 1,220,274 cases, 70% of which were used for training and 30% for testing”， in lines 760-761 of this revised manuscript. And all changes are highlighted.

11. It may improve the interpretability for researchers who are less familiar with ML to quantify the accuracy of the prediction model in more plain language. “Our model achieved X% accuracy, correctly classifying ASE in Y examples”.
Response: Thank you for your suggestion. In response, we revised the manuscript to include primarily descriptions of accuracy and assessment of the overall performance of the model to present the model’s accuracy in a more straightforward manner. This clarification can now be found in the Summary and Results sections, specifically in lines 66 to 68 and 411 to 415 of the revised manuscript. We believe this adjustment will make our findings more accessible to a broader range of researchers. Thank you for helping us improve the clarity of our presentation. All changes are highlighted.

30-Dec-2023

Mr. Jian-Feng Mao
Umea Universitet
Department of Plant Physiology
Umea 90736
China

RE: Accurate prediction of allele-specific gene expression in hybrid poplar facilitated by gapless genome assembly

Dear Dr. Jian-Feng Mao:

We are pleased to accept your manuscript for publication in the Plant Physiology. This acceptance is contingent on revision based on the comments of our reviewers. In particular, please consider the following:

Thank you for making the genome data available and including the LAI as previously suggested by reviewer 1, and please note the broken link identified by reviewer 2 below. As you can see, minor clarifications are requested by reviewer 2 and should be addressed very briefly in your revision.

Please highlight all changes and include a detailed annotation to changes to the text, with line numbers, and noting your responses to the comments.

To submit your revised manuscript, click:

Link Not Available

If you cannot return the revised manuscript within 8 weeks of receipt, please let us know. Otherwise, we will assume that you have elected not to revise the manuscript and are withdrawing it.

Thank you for allowing us to review your work. We look forward to hearing from you soon.

Sincerely,

Plant Physiology

-- Reviewer comments:
Reviewer #2 (Comments for the Author):

Thank you for taking the time to respond to my previous comments. You have satisfied most of them but I have some further follow up.

Comment 4. Thank you for incorporating LAI. LAI was just a suggestion of an additional metric, others may also be useful too, to emphasize the improvements in your assembly over previous assemblies, especially given your use of long read seq which should improve the assembly of repeat regions, including LTRs. However, your points about LAI are valid.

Comment 6. If there is space, you could just suggest a few potential methods for future investigation at the end of the sentence. I don't think there is a need for a detailed comparison to support your suggestions.

Additional comments:

Do you provide your training/testing/validation datasets as a matrix or table? I think this would improve the transparency if you uploaded the datasets to your GitHub or another repository so others can use your models with your dataset.

P28 L836: Figshare link appears to be broken.

I see you took measures to reduce overfitting through early stopping. Was cross validation conducted?

How were hyperparameters chosen? I don't see any mention of tuning.

Was feature selection performed using the entire dataset or just the training set?

Why was xgboost chosen over other models? This could be briefly explained on P12 L351.

I am suspicious of the near perfect R^2 of your regression model (model 3). What steps were taken to ensure overfitting is not occurring?

P3 L60: "Almost the entire telomeres and centromeres were assembled for each haplotype subgenome, leaving only two small gaps for one chromosome". I think this language is a bit confusing and sounds like you believe you assembled the entirety of each telomere and centromere in each chromosome with the exception of two gaps in the centromeres or telomeres in a single chromosome. Maybe something like "Almost all chromosomes, including the telomeres and centromeres, were completely assembled for each haplotype subgenome, with the exception of two small gaps existing on a single chromosome".

##

Reviewer #2 (Comments for the Author):

Overall:

Response:

Thank you for your feedback and the additional follow-up comments from the reviewer. We have addressed most of the concerns raised.

Comment regarding the first round of review: We appreciate the reviewer's feedback and are glad that we were able to satisfy most of their concerns. We will continue to address any remaining issues in order to improve the quality of our manuscript.

Follow-up questions related to machine learning and data: We understand the importance of these questions and have provided detailed responses to each of them. By addressing these questions, we hope to enhance the credibility and reproducibility of our study.

We would like to express our gratitude to the reviewer for their valuable feedback and for providing us with an opportunity to further clarify and improve our manuscript.

Comment 4. Thank you for incorporating LAI. LAI was just a suggestion of an additional metric, others may also be useful too, to emphasize the improvements in your assembly over previous assemblies, especially given your use of long read seq which should improve the assembly of repeat regions, including LTRs. However, your points about LAI are valid.

Response: Thank you for reviewing our work and providing valuable suggestions on using the LAI metric to evaluate assembly improvements. We highly value your feedback and have further discussed it in our previous response. Additionally, the advantages of long-read sequencing in improving assembly of repeat regions are significant. In this study, we were able to almost resolve all telomere and centromere regions, which was not achieved in previous assemblies. Once again, thank you for your valuable feedback, which has greatly helped our research.

Comment 6. If there is space, you could just suggest a few potential methods for future investigation at the end of the sentence. I don't think there is a need for a detailed comparison to support your suggestions.

Response: We appreciate the reviewer's suggestions. In our study, we acknowledge the limitations in conducting experiments to validate the association between inversions and TE accumulation at a small scale. Furthermore, for systematic investigation on a large (the whole genome) scale, it is still challenging to perform experimental or alternative validation methods. You know, we are mostly working on genomics and computational studies, and have really limited experience in experimental and functional studies. Therefore, given these constraints, we are unable to provide specific methods to support our suggestions for future investigations.

Additional comments:

1. Do you provide your training/testing/validation datasets as a matrix or table? I think this would improve the transparency if you uploaded the datasets to your GitHub or another repository so others can use your models with your dataset.

Response: Thank you for your valuable feedback. Yes, we have already uploaded the data of our training set and test set in matrix form to GitHub (https://github.com/shitianle77/84K_genome) and Figshare (https://figshare.com/articles/dataset/Gap-free_genome_assembly_of_hybrid_poplar_84K_/24279211). Furthermore, we have also uploaded the relevant codes for building the models and provided links for readers to access. We equally prioritize the transparency of our research and believe that this contributes to the advancement and quality of scientific research.

2. P28 L836: Figshare link appears to be broken.

Response: Thank you very much for your careful review of our article. We have now re-uploaded the data and made it publicly available. Links to relevant datasets and codes have also been updated in lines 821-825 of the revised manuscript.

3. I see you took measures to reduce overfitting through early stopping. Was cross validation conducted?

Response: Thank you for your review. In our study, we did not conduct cross validation to address overfitting. Instead, we implemented early stopping as a measure to prevent overfitting during the training of our model. We utilized early stopping as but at the same time we set higher nrounds. This approach allowed us to stop the training process when the validation metric did not improve, saving time and computational resources, and preventing overfitting. We appreciate your suggestion regarding cross validation and will consider it for future investigations.

4. How were hyperparameters chosen? I don't see any mention of tuning.

Response: Thank you for your question. During the process of model construction, we adjusted some parameters (such as subsample, nrounds, early_stopping_rounds, print_every_n, and used default parameters for others) and selected the best-performing model from them. Due to a lack of systematic testing, we did not elaborate on this in the manuscript. Additionally, due to limitations in computational resources, we did not extensively explore hyperparameters.

5. Was feature selection performed using the entire dataset or just the training set?

Response: Feature selection was performed based on the entire dataset. And the 46 features in Model 0 were selected through literature-based feature selection related to the influence on allele-specific gene expression (ASE). Concerns about potential high correlations among these 46 features led to the filtering out of some highly correlated but less important features (for details, see the Materials and Methods section in lines

764-767 of the revision manuscript.). As a result, 15 features were ultimately chosen for the construction and analysis of Models 1, 2, and 3.

6. Why was xgboost chosen over other models? This could be briefly explained on P12 L351.

Response: XGBoost is widely applied in various fields, including medicine (Nguyen et al., 2019) and civil engineering (Inoue et al., 2020). It has also been applied in plant study (soybean) and found to have an affinity for genotype and phenotype prediction (Gill et al., 2022). Moreover, in addition, the XGBoost model not only has high accuracy in classification models, but also performs excellently when dealing with tabular data problems (Zamani Joharestani et al., 2019). In addition, we provide a brief explanation in lines 347-349 of the revision manuscript.

Gill, M., et al. (2022). Machine learning models outperform deep learning models, provide interpretation and facilitate feature selection for soybean trait prediction. BMC Plant Biology, 22, 180.

Inoue, T., et al. (2020). XGBoost, a Machine Learning Method, Predicts Neurological Recovery in Patients with Cervical Spinal Cord Injury. Neurotrauma Rep, 1, 8-16.

Nguyen, H., et al. (2019). Developing an XGBoost model to predict blast-induced peak particle velocity in an open-pit mine: a case study. Acta Geophysica, 67, 477-490.

Zamani Joharestani, M., et al. (2019). PM2.5 Prediction Based on Random Forest, XGBoost, and Deep Learning Using Multisource Remote Sensing Data. 10, 373

7. I am suspicious of the near perfect R^2 of your regression model (model 3). What steps were taken to ensure overfitting is not occurring?

Response: Thank you for your review and feedback. We understand your concerns regarding the high R^2 value in our regression model (model 3). To ensure overfitting is not occurring, we have taken several steps. Firstly, we avoided using a training dataset that is too small within a reasonable range. If the training dataset size is too small, the model may overemphasize the noise and outliers in the training data, leading to poor generalization performance on new data. Secondly, in order to prevent the issue of insufficient generalization ability in the model, we strived to simplify the complexity of the model. We filter out highly correlated features to avoid overfitting caused by an excessive number of features during model training. Lastly, we compared the performance of different models and selected the one with the best trade-off between bias and variance. We hope this information addresses your concern.

8. P3 L60: “Almost the entire telomeres and centromeres were assembled for each haplotype subgenome, leaving only two small gaps for one chromosome". I think this language is a bit confusing and sounds like you believe you assembled the entirety of each telomere and centromere in each chromosome with the exception of two gaps in

the centromeres or telomeres in a single chromosome. Maybe something like "Almost all chromosomes, including the telomeres and centromeres, were completely assembled for each haplotype subgenome, with the exception of two small gaps existing on a single chromosome”.

Response: Yes. We have made modifications to this sentence according to your suggestion, which are in lines 59 to 61 in the revision manuscript. All changes are highlighted.

09-Jan-2024
Mr. Jian-Feng Mao
Umea Universitet
Department of Plant Physiology
Umea 90736
China

MSID: PP2023-RA-01090R2
MS TITLE: Accurate prediction of allele-specific gene expression in hybrid poplar facilitated by gapless genome assembly

Dear Dr. Jian-Feng Mao:

I am pleased to inform you that your revised manuscript "Accurate prediction of allele-specific gene expression in hybrid poplar facilitated by gapless genome assembly" has been accepted for publication in Plant Physiology. Your manuscript will be checked for consistency with journal formatting and image standards by one of our Science Editors, who will contact you shortly. They may have a number of queries and suggestions for you. Please keep in mind that they work to increase the online visibility of your article and its attraction for readers from the widest of backgrounds. You will have the opportunity to review any revisions, and I hope you will work constructively with them to get the most out of your research. You will also have the opportunity to review all changes when you receive the author proofs for your article.

Once validated for publication, your article will be sent to a Science Editor for a final review of your manuscript and source files. After your final files are uploaded, your article will appear in the next available issue and the online Preview version of your article will be posted after your signed Copyright and Checklist forms and final source files are received.

Please be sure to include the following in your manuscript file:
Harvard style citations
List of all author contributions and funding information at the beginning of manuscript after the titles and author list.
List all supplemental materials associated with the article, with titles, at the end of the ms, before the references and acknowledgments section.
Complete Figure Legends for all figures appearing in the article listed before the Literature Cited.

Thank you and congratulations on your Plant Physiology paper!

Sincerely,
Plant Physiology Board of Editors

=====
FIRST AUTHOR PROFILES
=====

We recognize the hard work that goes into being First Author of a Plant Physiology paper by publishing a "First Author Feature" for each First Author alongside your paper.

If the first author(s) would like to submit information for a feature on Facebook and Twitter, fill out the form by clicking on the link below:

Plant Physiology First Author Feature Form: https://forms.office.com/r/ZBCGZfhm8F
If you cannot access the form through the link above, please contact Rachel at rbelsky@aspb.org.

To upload an image of yourself to accompany your profile, please put your photo in the correct journal folder at the link below. You must label your image: Author First Name Last Name Article Title.

First Author Profile Image Dropbox: https://www.dropbox.com/scl/fo/xz029p7qjr2jml2rp2kmq/h?dl=0&rlkey=6fkxcaqmxx35ap152b2osy1fz

=====

IMPORTANT REMINDER: PEER REVIEW REPORTS
=====

If you opted to publish a peer review report along with your article during the original submission process, it will be prepared by the editorial staff and publicly posted with your manuscript, inside the zip file that contains any other supplemental material. As a reminder, the peer review report is a public record of all comments from editors and reviewers, as well as your prior responses, as you received them in the decision letters for each draft of your manuscript. If you agreed to publish this report and have changed your mind, or are not sure if you selected this option, please contact the editorial office as soon as possible before signing the license agreement from our publisher.

-- Reviewer comments:
